# MINDY-1 Is a Member of an Evolutionarily Conserved and Structurally Distinct New Family of Deubiquitinating Enzymes

**DOI:** 10.1016/j.molcel.2016.05.009

**Published:** 2016-07-07

**Authors:** Syed Arif Abdul Rehman, Yosua Adi Kristariyanto, Soo-Youn Choi, Pedro Junior Nkosi, Simone Weidlich, Karim Labib, Kay Hofmann, Yogesh Kulathu

**Affiliations:** 1 MRC Protein Phosphorylation & Ubiquitylation Unit, School of Life Sciences, University of Dundee, Dow Street, Dundee DD1 5EH, UK; 2 Institute for Genetics, University of Cologne, Zülpicher Straße 47a, 50674 Cologne, Germany

## Abstract

Deubiquitinating enzymes (DUBs) remove ubiquitin (Ub) from Ub-conjugated substrates to regulate the functional outcome of ubiquitylation. Here we report the discovery of a new family of DUBs, which we have named MINDY (motif interacting with Ub-containing novel DUB family). Found in all eukaryotes, MINDY-family DUBs are highly selective at cleaving K48-linked polyUb, a signal that targets proteins for degradation. We identify the catalytic activity to be encoded within a previously unannotated domain, the crystal structure of which reveals a distinct protein fold with no homology to any of the known DUBs. The crystal structure of MINDY-1 (also known as FAM63A) in complex with propargylated Ub reveals conformational changes that realign the active site for catalysis. MINDY-1 prefers cleaving long polyUb chains and works by trimming chains from the distal end. Collectively, our results reveal a new family of DUBs that may have specialized roles in regulating proteostasis.

## Highlights

• MINDY is a new family of DUBs consisting of FAM63A, FAM63B, FAM188A, and FAM188B
• MINDY DUBs are highly selective at cleaving K48-linked polyubiquitin
• Catalytic domain of MINDY-1 adopts a distinct fold with no homology to any known DUB
• Human MINDY-1 trims ubiquitin chains from the distal end

## Graphical Abstract

**Figure:**
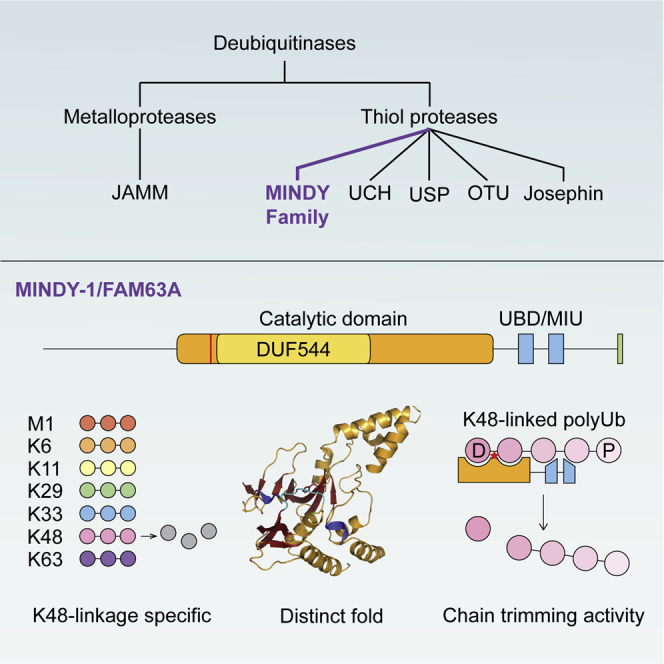

## Teaser

Abdul Rehman et al. discover a new family of deubiquitinating enzymes called MINDY. This structurally distinct family of DUBs is highly selective at cleaving K48-linked polyubiquitin chains.

## Introduction

Ubiquitylation is a post-translational modification (PTM) that regulates almost every facet of eukaryotic biology. The functional outcome of protein ubiquitylation is determined by the type of modification (monoubiquitin or polyubiquitin) and also the linkage type within the ubiquitin (Ub) chain (Kulathu and Komander, 2012). Ub binding domain (UBD)-containing proteins bind to different Ub modifications and link Ub signals to downstream signaling (Di Fiore et al., 2003). The outcome of Ub signals is regulated by deubiquitinating enzymes (DUBs), which are proteases that remove Ub from modified substrates (Reyes-Turcu et al., 2009). DUBs are therefore important regulators of the Ub system and regulate a plethora of cellular processes, including protein turnover, protein sorting, and trafficking (Clague et al., 2012, MacGurn et al., 2012). Indeed, deregulated DUB activity may promote human disease, and hence DUBs are being actively explored as potential drug targets (Heideker and Wertz, 2015, King and Finley, 2014). There are approximately 100 DUBs encoded in the human genome that can be classified into five families on the basis of the mechanism of catalysis (Clague et al., 2013, Nijman et al., 2005). Of the five families, four are thiol proteases, while the fifth includes metalloproteases (Reyes-Turcu et al., 2009).

One major function of ubiquitylation is in protein degradation, a precise proteolytic process essential for the turnover of many proteins and also for removing damaged proteins (Eletr and Wilkinson, 2014). Modification of proteins with K48-linked polyUb chains, the most abundant linkage type detected in cells, is the canonical signal that marks proteins for degradation by the 26S proteasome (Finley, 2009, Kulathu and Komander, 2012). Prior to degradation, proteasome-associated DUBs release Ub from substrates to recycle Ub (Inobe and Matouschek, 2014). Some DUBs can trim the K48 chains on ubiquitylated substrates to rescue them from degradation (Lam et al., 1997, Lee et al., 2010, Rumpf and Jentsch, 2006), highlighting the fine control that can be exerted by DUBs.

Here we make the surprising discovery that an uncharacterized protein, FAM63A, is a deubiquitinase that is highly selective at hydrolyzing K48-linked polyUb. We delineate this deubiquitinating activity to be encoded within a previously unannotated domain. Our structural analyses reveal that the catalytic domain of FAM63A is a distinct folding variant of the superfamily of cysteine proteases. FAM63A has no homology to any of the known DUBs, so we classify this newly discovered Ub protease to be a prototype of a new family of DUBs. Defining FAM63A as a DUB led to the identification of further members that form part of this family.

## Results and Discussion

**Figure 1:**
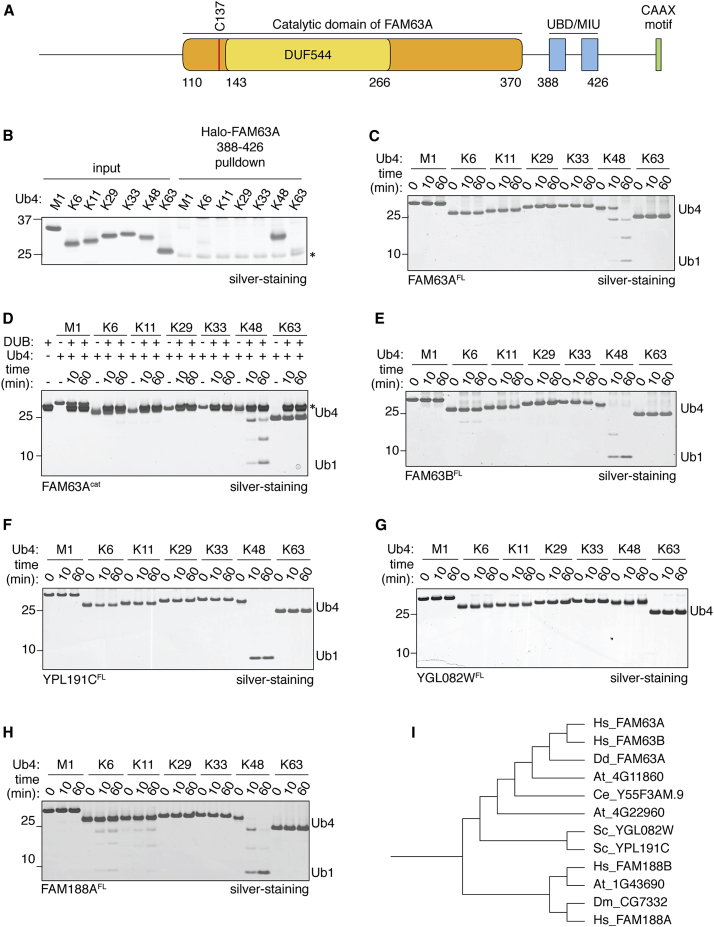
Identification of the MINDY Family of DUBs (A) Schematic representation of the domain structure of human FAM63A. (B) Halo-tagged FAM63A (388–426) coupled to HaloLink resin was incubated with tetraUb of the indicated linkage types. The captured materials were separated on 4%–12% SDS-PAGE gel and silver stained. (C–H) DUB assays testing activity and specificity of polyUb cleavage by FAM63A (C), putative catalytic domain of FAM63A (110–384) (D) (asterisk indicates FAM63A), full-length FAM63B (E), YPL191C/MIY1 (F), YGL082W (G), and full-length human FAM188A (H); 1.6 μM of DUBs were incubated with 2.2 μM of tetraUb chains for the indicated time. (I) Phylogenetic tree of MINDY family DUBs based on alignment of catalytic domains (Figure S2B). See also Figure S1.

In order to understand linkage selectivity in Ub signaling, we recently developed methods to assemble tetraUb chains of different linkage types that allow us to profile linkage selectivity of UBDs (Kristariyanto et al., 2015b). The motif interacting with Ub (MIU) is a small UBD consisting of a helical motif that binds to monoUb (Lee et al., 2006, Penengo et al., 2006). When investigating this class of UBDs, we identified FAM63A to contain MIU motifs with high selectivity for binding K48-linked polyUb (Figures 1A, 1B, and S1A). In addition to this K48-binding UBD, FAM63A contains a domain of unknown function (DUF544) (Figure 1A). Given the presence of a UBD, we posited a Ub-dependent function for the associated DUF domain (Marcotte et al., 1999). Sequence conservation within the FAM63 family suggested the presence of a cysteine protease active site, and therefore we hypothesized that it may be a Ub protease. To test this hypothesis, we performed DUB assays in which we monitored in vitro cleavage of different linkages of tetraUb chains, which shows that full-length FAM63A readily cleaves K48 chains (Figure 1C). In contrast, all other chain types are intact. This revealed that FAM63A is a DUB with unique selectivity for cleaving K48-linked polyUb. To identify the catalytic domain in FAM63A, we used multiple sequence alignment and secondary structure prediction (Kelley et al., 2015), on the basis of which we predicted the middle region of FAM63A (residues 110–384), which also includes DUF544, to be the catalytic domain (Figures 1A and S1C). Indeed, this predicted domain encodes catalytic activity that maintains high specificity for K48 linkages (Figures 1D, S1B, and S1D).

Human FAM63B is another DUF544-containing protein that shares sequence similarity and domain organization with FAM63A (Figure S2A). DUB assays reveal that FAM63B also cleaves polyUb chains (Figure 1E). Remarkably, both FAM63A and FAM63B are highly selective at hydrolyzing K48-linked polyUb and do not cleave any of the other linkage types tested. There are several human DUBs that contain additional UBDs, but to our knowledge this is the first instance of DUBs with MIU motifs (Clague et al., 2013). Therefore, FAM63 members are unannotated human DUBs that do not bear sequence similarity to any of the known DUBs. We classify these newly discovered enzymes to be a distinct family of DUBs, which we name MINDY (MIU-containing novel DUB family), with FAM63A as MINDY-1. Orthologs of FAM63 are also present in plants, budding yeast and *Dictyostelium*. Notably, the specificity toward cleaving K48-linked polyUb is conserved in yeast YPL191C (Figure 1F), hereafter named MIY1 (MINDY deubiquitinase in yeast). In contrast, the other yeast homolog, YGL082W, does not show any deubiquitinating activity despite the conserved catalytic residues being present (Figures 1G, S2B, and S2E).

Having identified MINDY-1/FAM63A as a new DUB, we wondered if there were other more distantly related proteins that also form part of the MINDY DUB family. Sequence analysis reveals that FAM188 members (FAM188A and FAM188B in humans) are related to FAM63 (Figure S2B). DUB assays of FAM188A against tetraUb reveal that FAM188A is also a DUB, with predicted catalytic residues C51 and H287 (Figures 1H and S2B). Interestingly, FAM188A is described as a caspase interacting pro-apoptotic protein and tumor suppressor (Liu et al., 2002, Shi et al., 2011) and has an EF hand motif inserted into the catalytic domain (Figure S2A). Despite being divergent from FAM63, FAM188A is also highly selective for the cleavage of K48-linked polyUb. Hence, we include FAM188 members in the highly conserved MINDY family of DUBs (Figure 1I).

**Figure 2:**
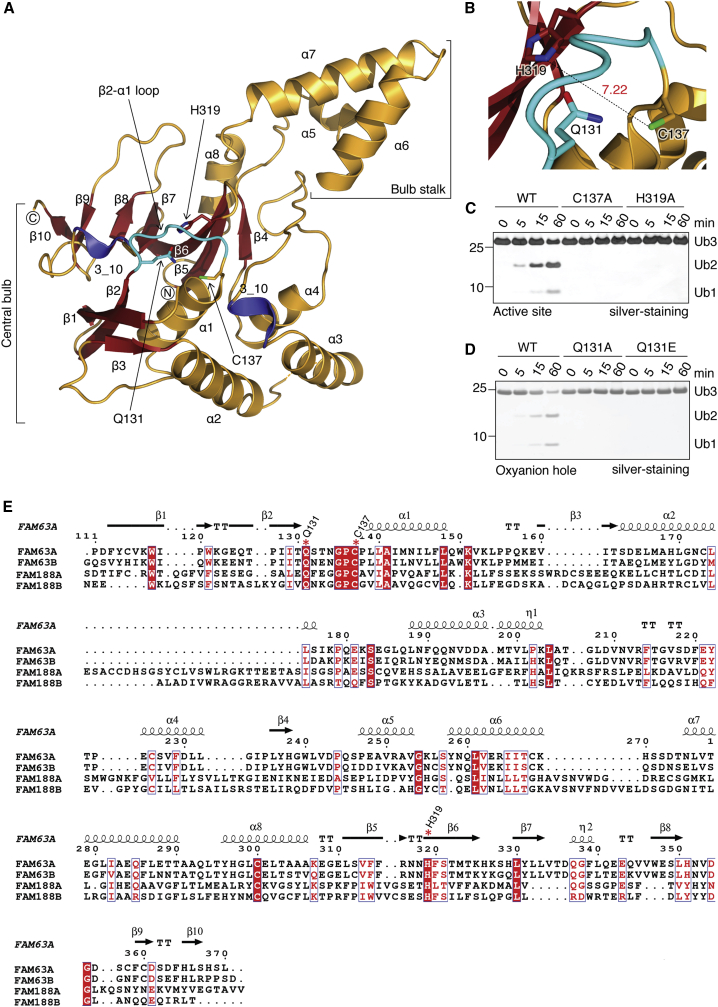
Crystal Structure of MINDY-1^cat^ (A) Structure of catalytic domain of FAM63A/MINDY-1^cat^ (110–370). The Cys loop (cyan) and the catalytic residues are indicated. β sheets are colored red and 3_10 helices blue. (B) A close-up image of the MINDY-1^cat^ catalytic site. Q131, C137, and H319 are shown. (C and D) Hydrolysis of 1.9 μM K48-linked triUb by 1.6 μM MINDY-1 wild-type (WT) and the indicated mutants of the active site residues (C) or Q131 that forms the oxyanion hole residue (D). (E) Sequence alignment of human FAM63A, FAM63B, FAM188A, and FAM188B. Secondary structure elements are shown for MINDY-1^cat^. The catalytic residues are highlighted with red asterisks. Residues 300–371 of FAM188A that form the EF hand domain have been omitted from the alignment. Fully conserved residues are shaded in red. See also Figures S2 and S3.

In order to determine the mechanism of this newly identified DUB, we crystallized the minimal catalytic domain of FAM63A/MINDY-1 spanning residues 110–384 (MINDY-1^cat^). The structure of MINDY-1^cat^ domain was determined at 3 Å resolution by X-ray crystallography (Table 1). The asymmetric unit (ASU) contains one molecule of MINDY-1^cat^ with discernible electron density for residues 110–370. The catalytic core domain of MINDY-1 with a dimension of 32 × 64 × 36 Å resembles a light bulb consisting of two subdomains, a central “bulb” subdomain that sits on a “stalk” subdomain, which resembles the base of the bulb (Figures 2A and S2C). The core of the central bulb subdomain contains a seven-stranded β sheet (β4–β10), where β7 is connected to β8 by a short 3_10_-helix (Figures S2D and S2E). The stalk subdomain, made up of three α helices (α5–α7), forms a long helical arm that protrudes away from the central domain. A Dali search against structures in the Protein Data Bank (PDB) did not identify any matches with significant *Z* scores or sequence identity (Holm and Rosenström, 2010). Given the low homology to known protein structures, we classify MINDY-1^cat^ as a new folding variant of the diverse superfamily of cysteine proteases (Figure S3). The structure of MINDY-1^cat^ does not bear close homology to any of the known DUBs, further supporting our classification of MINDY as a new family of DUBs.

Cysteine-based DUBs usually have a catalytic triad with a catalytic Cys, a nearby His that lowers the p*K*_a_ of the catalytic Cys for nucleophilic attack, and a third residue, usually an Asp or Asn, that stabilizes the catalytic His (Reyes-Turcu et al., 2009). We predict the conserved C137 at the N terminus of helix α1 and H319 on the adjacent β6 to be the catalytic residues and these are present in a C-H architecture typical of papain-like peptidases (Figures 2B and 2E). The third catalytic residue, which serves to polarize the catalytic His, is not obvious in the observed crystal structure. Mutation of either C137 or H319 to Ala completely abolishes catalytic activity of FAM63A toward K48-linked polyUb (Figure 2C). A conserved Gln (Q131) residue N-terminal of the catalytic Cys residue may form the oxyanion hole to stabilize the negative potential formed on the carbonyl oxygen atom of the scissile bond in the transition state. Indeed, mutation of Q131 to Ala or Glu completely abolishes catalytic activity (Figure 2D). However, in the structure of isolated MINDY-1^cat^, the active site is in an unproductive conformation and C137 is rotated out of hydrogen bond distance from the other catalytic residues (Figure 2B). Therefore, the observed structure is MINDY-1 in an inhibited state.

**Figure 3:**
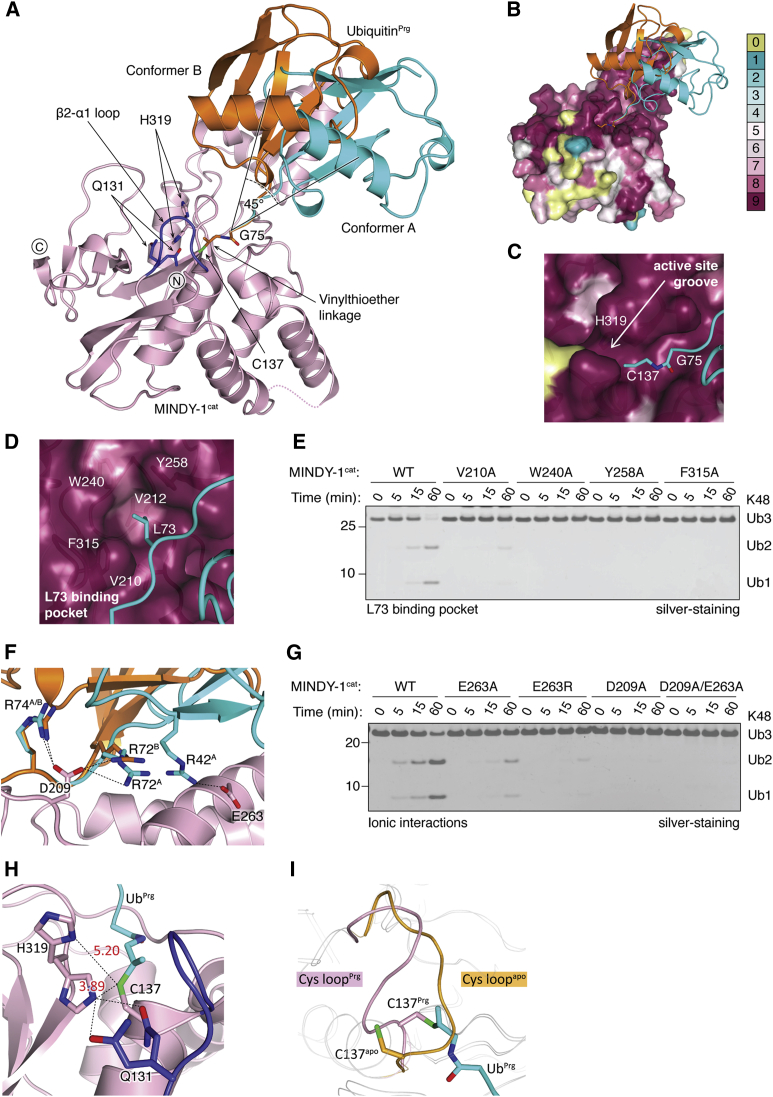
Structure of MINDY-1^cat^∼Ub (A) Overall structure of the catalytic core domain of MINDY-1 (pink) covalently bound to Ub. Ub exists in two alternate conformers in the structure that are rotated by ∼45° (cyan and orange). The vinylthioether linkage connecting Ub^Prg^ with MINDY-1 is shown in sticks. The Cys loop (β2-α1) is shown in blue. (B) Conserved residues on the surface of MINDY-1 based on the sequence alignment in Figure S1C generated with the Consurf server (http://consurf.tau.ac.il) are shown. While the backside of MINDY-1^cat^ is not conserved, surfaces interacting with and around the distal Ub are conserved. (C) Close-up view of the catalytic groove where the C terminus of Ub sits, with coloring scheme as in (B). (D) An aromatic cage formed by V212, W240, Y258, and F315 interacts with L73 of Ub. Close-up view of the conserved hydrophobic pocket accommodating L73 colored as in (B). (E) DUB assays monitoring cleavage of 1.9 μM K48-triUb with 1.6 μM MINDY-1^cat^ performed as in Figure 1C comparing activity of MINDY-1 and point mutants lining the L73 pocket: V210A, W240A, Y258A, and F315A. (F) Close-up view of ionic interactions between Ub and MINDY-1. (G) DUB assays comparing activity of MINDY-1 mutants that disrupt ionic interactions with Ub as performed in (E). (H) Close-up image of the MINDY-1 catalytic triad showing two alternate conformations for H319 and Q131. Distances to C137 are indicated by dotted lines. (I) Superposition of apo and complex states of MINDY-1^cat^ shows movement of the Cys loop (apo in orange and MINDY-1^cat^∼Ub complex in pink). See also Figure S4.

To gain insights into this newly discovered family of DUBs, we focused our efforts on understanding the catalytic mechanism of MINDY-1. C-terminally propargylated Ub (Ub^Prg^) is a potent and selective covalent inhibitor of most thiol DUBs and forms a vinylthioether linkage with the catalytic cysteine (Ekkebus et al., 2013). We purified and crystallized a covalent complex between MINDY-1^cat^ and Ub^Prg^ (MINDY-1^cat^∼Ub) and determined its structure by molecular replacement (Figures S4A–S4D and Table 1). Such monoUb complexes represent a product-intermediate state with the distal Ub bound in the S1 site. There is one MINDY-1^cat^∼Ub complex in the ASU. Although the electron density for MINDY-1^cat^ is well ordered, the electron density for Ub was not easily interpretable. Refining the structure at lower resolution revealed that Ub exists in two alternate conformations (Ub^A^ and Ub^B^), with each at ∼50% occupancy (Figures 3A, S4C, and S4D). The distal Ub rests on the stalk subdomain, and the region of MINDY-1^cat^ that makes contact with Ub is conserved in evolution (Figure 3B). Mapping conserved residues on the surface of MINDY-1^cat^ reveals conserved regions around and opposite to where the distal Ub makes contacts, suggesting where the proximal Ub might bind (Figure 3B). The C terminus of Ub sits in a conserved catalytic groove, and L73 of Ub sits in a highly conserved hydrophobic pocket (Figures 3C and 3D). Mutating residues lining this hydrophobic pocket to disrupt interactions with the C terminus of Ub abolishes catalytic activity (Figure 3E). MINDY-1 therefore is similar to many other DUBs that require an interaction with L73 of the distal Ub for catalysis (Békés et al., 2013). MINDY-1 also mediates ionic interactions with the distal Ub, and mutating the key interacting residues in MINDY-1^cat^ completely abolishes catalytic activity of the DUB (Figures 3F and 3G).

Binding of Ub^Prg^ to MINDY-1^cat^ does not induce global conformational changes in the catalytic domain, and both free and MINDY-1^cat^∼Ub complex superpose well (root-mean-square deviation ∼ 1 Å over 244 aligned Cα atoms). One notable difference between free and complexed states of MINDY-1^cat^ is the movement of the Cys loop (β2-α1) that in the structure of apo MINDY-1^cat^ blocks access of Ub to the catalytic site (Figures 3H, 3I, 2B, S4E, and S4F). Movement of the Cys loop also rotates the catalytic C137 to bring it closer to the catalytic H319. Intriguingly, we observe H319 to exist in two alternate conformations (Figure 3H). In one conformation, H319 is closer to the C137, resembling an active site poised for catalysis. In an alternate conformation, H319 is flipped away from C137 in a conformation in which the DUB is again in an inhibited state. Our analyses point to a substrate-induced conformational change that remodels the Cys loop and realigns the catalytic residues to an active conformation.

**Figure 4:**
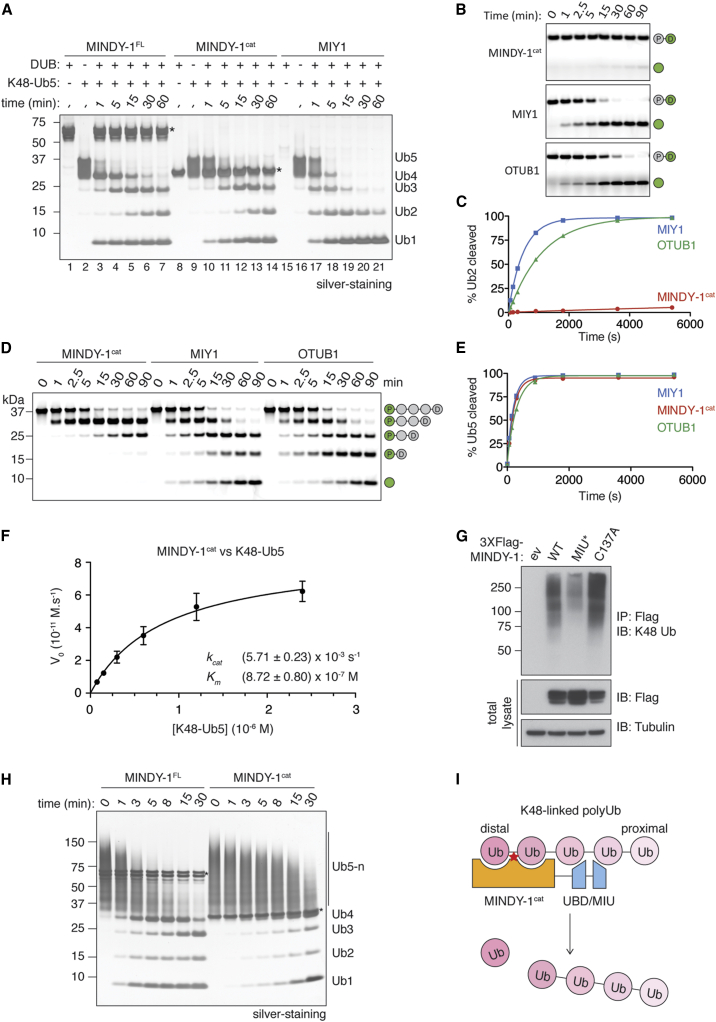
MINDY-1 Cleaves PolyUb Chains in a Stepwise Manner (A) Time course of cleavage of 3.5 μM K48-pentaUb by 1.6 μM of full-length MINDY-1 and MINDY-1^cat^ and 160 nM MIY1. Asterisks indicate MINDY-1. (B) Kinetics of cleavage of fluorescently labeled K48-linked diUb by MINDY-1^cat^, MIY1, and OTUB1. DUBs (1 μM) were incubated with 500 nM of K48-linked diUb that has been labeled with an infrared fluorescent dye at its distal Ub (green circle) for the indicated times. Fluorescent Ub was visualized using Odyssey LI-COR system at 800 nm channel. D, distal Ub, P, proximal Ub. (C) Quantification of K48-Ub2 hydrolysis by MINDY-1^cat^, MIY1, and OTUB1 in (B). Percentage of the formed Ub1 intensity is shown on the y axis (n = 3; mean ± SD). (D) DUB assays monitoring time-dependent cleavage of fluorescently labeled pentaUb by MINDY-1^cat^, MIY1, and OTUB1 as in (B). The proximal Ub of the chain (indicated by green circle) was labeled with an infrared fluorescent dye. (E) Quantification of cleavage of K48-linked pentaUb by MINDY-1^cat^, MIY1, and OTUB1 in (D). The percentage of the total intensities of Ub4, Ub3, Ub2, and Ub1 formed is shown on the y axis (n = 3; mean ± SD). See also Figure S5. (F) Steady-state kinetics of K48-linked pentaUb cleavage by MINDY-1^cat^. MINDY-1^cat^ (15 nM) was incubated with 0.075–2.4 μM fluorescently labeled pentaUb (IR-K48-Ub5). The K48-Ub4 formed at the early time point (less than 10% of the substrate) was quantified to obtain initial velocities (V_0_). V_0_ was plotted against IR-K48-Ub5 concentration, and the data were fitted to the Michaelis-Menten equation to derive *k*_cat_ and *K*_m_ (n = 3; mean ± SD). (G) Flag pull-downs from extracts of HEK293 cells inducibly expressing the indicated full-length MINDY-1 constructs. ev, empty vector; MIU^∗^, MIU mutant, L415A/A416G. Immunoblotting with a K48-linkage specific antibody was performed to monitor captured polyUb material. (H) Time course comparing hydrolysis of K48-polyUb chains containing at least 5 Ub by full-length MINDY-1 and MINDY-1^cat^, which lacks the MIU. (I) Model depicting the synergy between different domains of MINDY-1, where the UBD mediates substrate targeting to result in trimming of the Ub chain from the distal end by the catalytic domain. See also Figure S5.

DUBs can either cleave within chains (endo-DUB) or remove Ubs from one end of the chain (exo-DUB), and the mode of cleavage used by a DUB provides insights into its function (Komander et al., 2009). For instance, CYLD, a negative regulator of NF-κB signaling, cleaves within chains to release Ub chains en bloc from substrates (Komander et al., 2008). In contrast, DUBs such as USP14 trim Ub chains and can edit the degradation signal on substrates to rescue them from the proteasome (Lee et al., 2010). To determine the mode of chain cleavage used by MINDY-1, we carefully monitored the time-dependent cleavage of K48-linked pentaUb chains (Figure 4A). Upon cleavage by MINDY-1^FL^ and MINDY-1^cat^, tetraUb and monoUb are formed at the earliest time points, followed by the appearance of triUb (Figure 4A, lanes 3, 4, 10, and 11). DiUb is detected only at later time points, while there is a steady increase in the intensity of monoUb from the start (Figure 4A, lanes 3–7 and 10–14). This suggests that MINDY-1 cleaves Ub chains in a stepwise manner, releasing one Ub at a time (exo-DUB), and this mode of cleavage is not influenced by the MIU. In contrast, yeast MIY1 does not discriminate between the positions at which it cleaves within a Ub chain (endo-DUB), so all cleavage products (tetra-, tri-, di-, and monoUb) are formed at similar rates (Figure 4A, lanes 17–21). MINDY-1 has poor Ub C-terminal hydrolytic activity (Figure S5A), and two conformations are observed for the distal Ub (Figure 3A), which further suggests that the preferred substrate of MINDY-1 is Ub chains.

To explore the efficiency of MINDY-1 to cleave K48-polyUb chains, we set out to determine the kinetics of MINDY-1 and to compare its activity with OTUB1, a well-studied DUB, which is also highly selective at cleaving K48-linked chains (Wang et al., 2009). Using in-gel-based DUB assays, we monitored the cleavage of K48-linked diUb substrate that carries an infrared fluorescent dye at the distal end. We found that MINDY-1^cat^ cleaves diUb poorly, whereas MIY1 and OTUB1 both efficiently cleave diUb substrates (Figures 4B and 4C). Similarly, MINDY-1 cleaves triUb weakly, suggesting that diUb and triUb are poor substrates for MINDY-1 (Figures S5C and S5D).

Several DUBs have been reported to preferably cleave long polyUb chains (Békés et al., 2015, Todi et al., 2009). To investigate whether long polyUb chains are the preferred substrate of MINDY-1, we assembled K48-linked pentaUb chains with an infrared fluorescent label incorporated at the proximal end. When these fluorescently labeled chains were used as the substrate, MINDY-1^cat^ was as efficient as MIY1 and OTUB1 at cleaving pentaUb (Figures 4D and 4E). Interestingly, even though the rates of K48-Ub5 cleavage of MIY1 and OTUB1 are comparable with MINDY-1^cat^, the cleavage products generated by MIY1 and OTUB1 range from monoUb to tetraUb, further supporting endo-DUB activity for MIY1 and OTUB1 (Figures 4A and 4D). On the other hand, the chain-trimming activity of MINDY-1 is clearly demonstrated by the formation of tetraUb and the subsequent formation of triUb at later time points (Figure 4D). Moreover, because the proximal Ub is fluorescently labeled, these assays also reveal a marked directionality in chain cleavage where MINDY-1 cleaves polyUb chains from the distal end. Next, we used pentaUb for kinetic analysis, because MINDY-1 cleaves pentaUb efficiently, and tetraUb is the only product formed at the early time points (Figure 4D). We determined the *k*_cat_ and *K*_m_ of MINDY-1 for K48-Ub5 to be ∼5.71 × 10^−3^ s^−1^ and ∼872 nM, respectively. The low *K*_m_ values suggest a strong interaction of MINDY-1 with pentaUb chains. Taken together, these results demonstrate that MINDY-1 prefers to trim longer K48-polyUb chains from the distal end.

MINDY-1 is a modular enzyme with tandem UBDs (MIU motifs) that binds selectively to K48-linked polyUb and a catalytic domain that selectively cleaves the same linkage type. In DUBs such as OTUD1 and OTUD2, the associated UBDs regulate chain linkage specificity of the DUBs (Mevissen et al., 2013). However, in MINDY-1, the UBD does not influence linkage specificity of the DUB, as the minimal catalytic domain still maintains selectivity for K48-linked polyUb (Figures 1C and 1D). To investigate the potential roles of MIU in MINDY-1, we expressed wild-type Flag-MINDY-1 or MIU mutant in HEK293 cells. Flag immunoprecipitations from cell extracts reveal high-molecular-weight K48-linked polyubiquitylated proteins associated with wild-type MINDY-1 (Figures 4G and S5I). The amount of ubiquitylated proteins associated with MINDY-1 is reduced when the MIU is mutated, suggesting a role for the MIU in targeting MINDY-1 to K48-polyubiquitylated potential substrates.

If the MIU of MINDY-1 mediates substrate targeting, then we expect it to influence the catalytic activity and cleavage of Ub chains by MINDY-1. We observed that MIU does not affect MINDY-1 activity toward K48-Ub5 chains (Figure 4A, lanes 1–14). Because MINDY-1 prefers to cleave longer polyUb chains (Figure S5H), we repeated the investigations using long K48-linked polyUb chains (pentaUb or longer) as substrates. Surprisingly, we now observe that in the absence of MIU, cleavage of long K48-chains is significantly compromised (Figure 4H). Hence in MINDY-1, the MIU regulates substrate targeting and is crucial for cleavage of long polyUb chains.

Collectively, our analysis of human MINDY-1 reveals that it is a chain-trimming enzyme, which uses the Ub-binding MIU together with the catalytic domain to cleave K48-linked polyUb chains from the distal end in a stepwise manner (Figure 4I). It is fascinating that this DUB, in addition to sensing polyUb chain linkage, also appears to sense chain length and detect the distal end of a chain. A lingering question in the field of Ub research is how long Ub chains have to be in cells in order to elicit a response. A long-held idea has been that K48 chains have to be at least four Ubs in length for efficient recognition and degradation of substrate proteins by the proteasome (Thrower et al., 2000). Recent work suggests that distributed shorter chains are more effective degradation signals (Lu et al., 2015). On the other hand, USP14 and yeast OTU1 trim K48 signals on substrates to rescue them from proteasomal degradation (Lee et al., 2010, Rumpf and Jentsch, 2006). Long K48-linked polyUb chains are poor substrates of several USP DUBs (Schaefer and Morgan, 2011). With its exquisite specificity for trimming K48 chains, MINDY-1 may alter the fate of proteins marked for degradation. Notably, while all MINDY family members analyzed are highly selective at cleaving K48-linked polyUb, we observe differences in their mode of action. With different domain architectures, expression, subcellular localization, and mode of action, we envisage diverse functional roles for this new family of DUBs.

## Experimental Procedures

### cDNA and Antibodies

All the cDNA constructs used in this study were generated by the cloning team of the Division of Signal Transduction Therapy, MRC Protein Phosphorylation and Ubiquitylation Unit, University of Dundee, United Kingdom (Table S1). All constructs are available on request from the MRC Protein Phosphorylation and Ubiquitylation Unit reagents Web page (http://mrcppureagents.dundee.ac.uk).

Protein and epitope tags were detected by western blotting using the following antibodies: anti-K48-linked polyUb chains (05-1307; Millipore), anti-Ub antibody (Z0458; DAKO), anti-Flag M2 antibody (F1804; Sigma-Aldrich), and anti-α-Tubulin antibody (3873; Cell Signaling Technology).

### Bioinformatics

Sequence database searches with generalized profiles (Bucher et al., 1996) or hidden Markov models (HMMs) (Eddy, 1998) created from multiple alignments of the FAM63 catalytic domain failed to find significant relatives outside the FAM63 family, with members of the FAM188 family yielding slightly sub-significant scores. However, the application of the HHSEARCH software (Söding, 2005), which performs HMM-to-HMM comparisons, allowed to establish a significant relationship between the FAM63 and FAM188 families. An HMM made from a FAM63 alignment matched the FAM188 family with a p value of 9.2 × 10^−7^. Conversely, an HMM made from the FAM188 family matched the FAM63 family with a p value of 7.9 × 10^−7^. The resulting HMM-to-HMM alignment revealed a perfect conservation of the active site residue, suggesting a functional similarity between the two distantly related protein families. Multiple sequence alignments were created in Jalview (Waterhouse et al., 2009) by using the L-INS-I algorithm of the MAFFT package (Katoh and Toh, 2010). Phylogenetic trees were generated using PhyML (Dereeper et al., 2008) (http://phylogeny.lirmm.fr) using bootstrapping with alignment of catalytic domains as input and trees rendered using TreeDyn (http://phylogeny.lirmm.fr).

### UBD Linkage Specificity Analysis

TetraUb chains of the different linkage types were assembled and purified as described previously (Kristariyanto et al., 2015a, Kristariyanto et al., 2015b). Briefly, 58.5 nM tetraUb chains in 500 μl pull-down buffer (50 mM Tris-HCl [pH 7.5], 150 mM NaCl, 0.1% NP-40, 1 mM DTT, 0.5 mg/ml BSA) was captured using 10.5 nmol Halo-MINDY-1 (388–426) immobilized on HaloLink resin for 2 hr at 4°C. Captured materials were analyzed on silver-stained SDS-PAGE gel. For details, see Supplemental Experimental Procedures.

### Crystallization and Structure Determination

MINDY-1^cat^ and MINDY-1^cat^-Ub^Prg^ complex were purified and crystallized and their structures determined as detailed in Supplemental Experimental Procedures.

## Author Contributions

S.A.A.R., Y.A.K., and S.-Y.C. designed and performed all experiments. S.A.A.R. performed structure determination and analysis. P.J.N. and K.L. contributed with reagents and analysis. K.H. performed sequence analysis. S.W. cloned all the DNA constructs. Y.K. supervised the study and wrote the manuscript with input from all the authors.

## Figures and Tables

**Table 1:** Data Collection and Refinement Statistics

	MINDY-1^cat^ (Anomalous)	MINDY-1^cat^ (Native)	MINDY-1^cat^∼Ub (Complex)
**Data Collection**			

Beamline	I02, DLS	ID23-1, ESRF	ID29, ESRF
Space group	P4_1_22	P4_1_22	P6_5_22
a, b, c (Å)	100.55, 100.55, 165.64	99.67, 99.67, 165.12	82.33, 82.33, 332.46
α, β, γ (°)	90.00, 90.00, 90.00	90.00, 90.00, 90.00	90.00, 90.00, 120.00
Wavelength (Å)	1.0073	0.93927	0.97623
Resolution (Å)	63.93–3.39 (3.39–3.30)^a^	48.18–3.0 (3.18–3.0)	48.63–2.65 (2.78–2.65)
R-merge	0.35 (4.31)	0.065 (0.883)	0.089 (0.595)
I/σ(I)	9.8 (1.2)	14.4 (2.1)	17.1 (3.3)
Completeness (%)	99.7 (99.2)	99.8 (99.9)	99.9 (100.0)
Multiplicity	13.8 (13.5)	5.0 (5.2)	9.4 (10.0)
CC1/2	0.996 (0.526)	0.998 (0.866)	0.999 (0.925)

**Refinement**			

Resolution (Å)		48.18–3.0	48.63–2.65
No. of reflections		17,325 (2,732)	20,518 (2,621)
R_*work*_*/*R_*free*_		0.197/0.242	0.205/0.231

**No. of Atoms**			

Protein		2,009	3,078
Waters		0	53
Ligand/ion		13	26

**B Factors (Å^2^)**			

Wilson B		97.80	51.5
Protein		120.74	71.55
Ligand/Ion		130.70	78.20
Water			50.64

**RMSDs**			

Bond length (Å)		0.007	0.008
Bond angles (°)		1.204	1.104
Ramachandran statistics (favored/allowed/outliers)		95.0/5.0/0.0	97.0/3.0/0.0

DLS, Diamond Light Source; ESRF, European Synchrotron Radiation Facility; RMSD, root-mean-square deviation.

a Values for the highest-resolution shell are shown in parentheses.

## Accession Numbers

The accession numbers for the structures reported in this paper are PDB: 5JKN and 5JQS.

## Supplemental Information

Document S1. Supplemental Experimental Procedures, Figures S1–S5, and Table S1

Document S2. Article plus Supplemental Information
