# Supplementary material for: MINDY-1 Is a Member of an Evolutionarily Conserved and Structurally Distinct New Family of Deubiquitinating Enzymes
Source: Mol Cell. 2016 Jul 7;63(1):146–55. doi: 10.1016/j.molcel.2016.05.009 (PMC4942677; doi:10.1016/j.molcel.2016.05.009)
Supplement: Document S1. Supplemental Experimental Procedures, Figures S1–S5, and Table S1 [file mmc1.pdf]

Molecular Cell, Volume *63*

## Supplemental Information

# MINDY-1 Is a Member of an Evolutionarily Conserved and Structurally Distinct New Family of Deubiquitinating Enzymes

**Syed Arif Abdul Rehman, Yosua Adi Kristariyanto, Soo-Youn Choi, Pedro Junior Nkosi, Simone Weidlich, Karim Labib, Kay Hofmann, and Yogesh Kulathu**

**Supplemental Materials:**

**Supplemental Figures**

## Figure S1

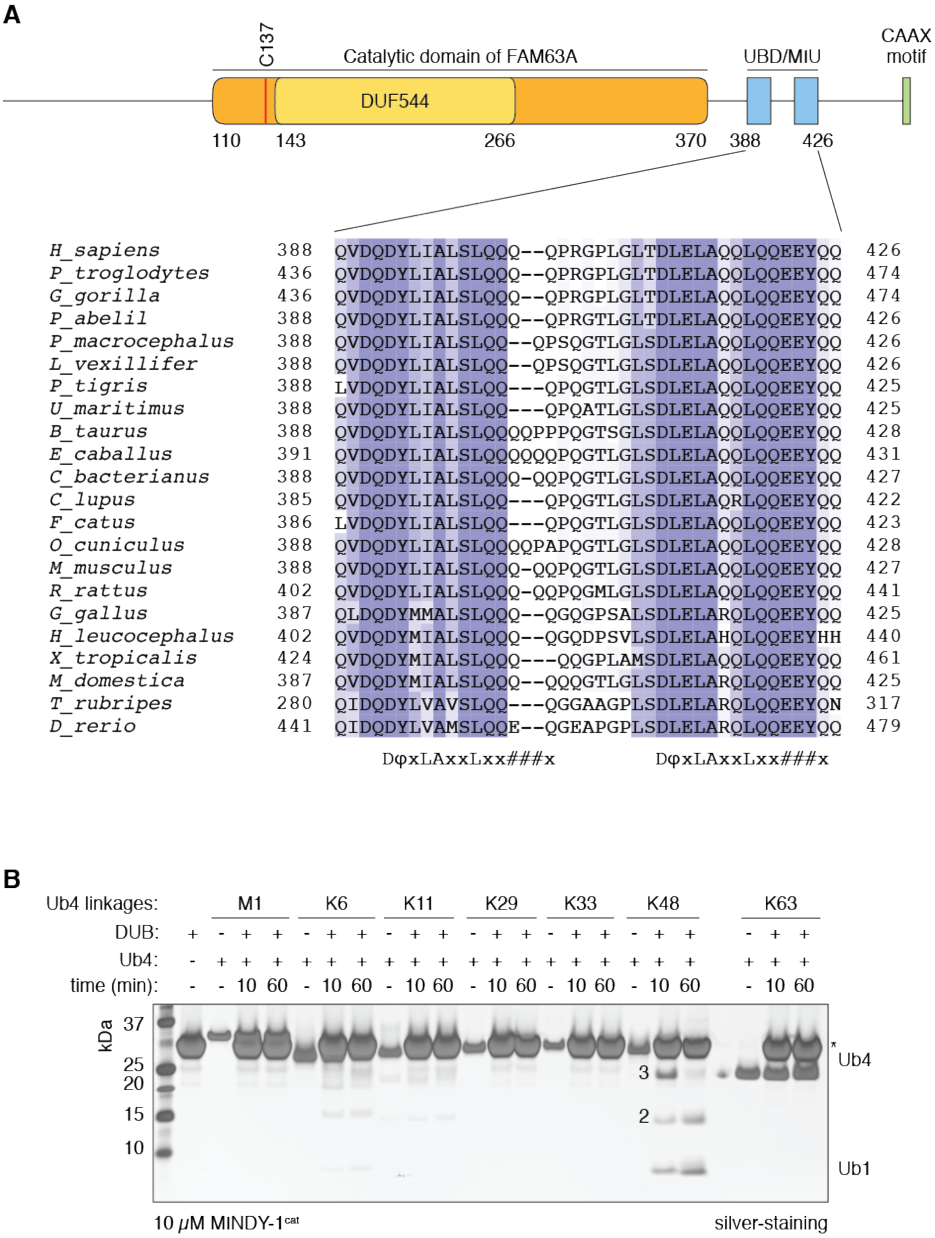

**Figure S1**

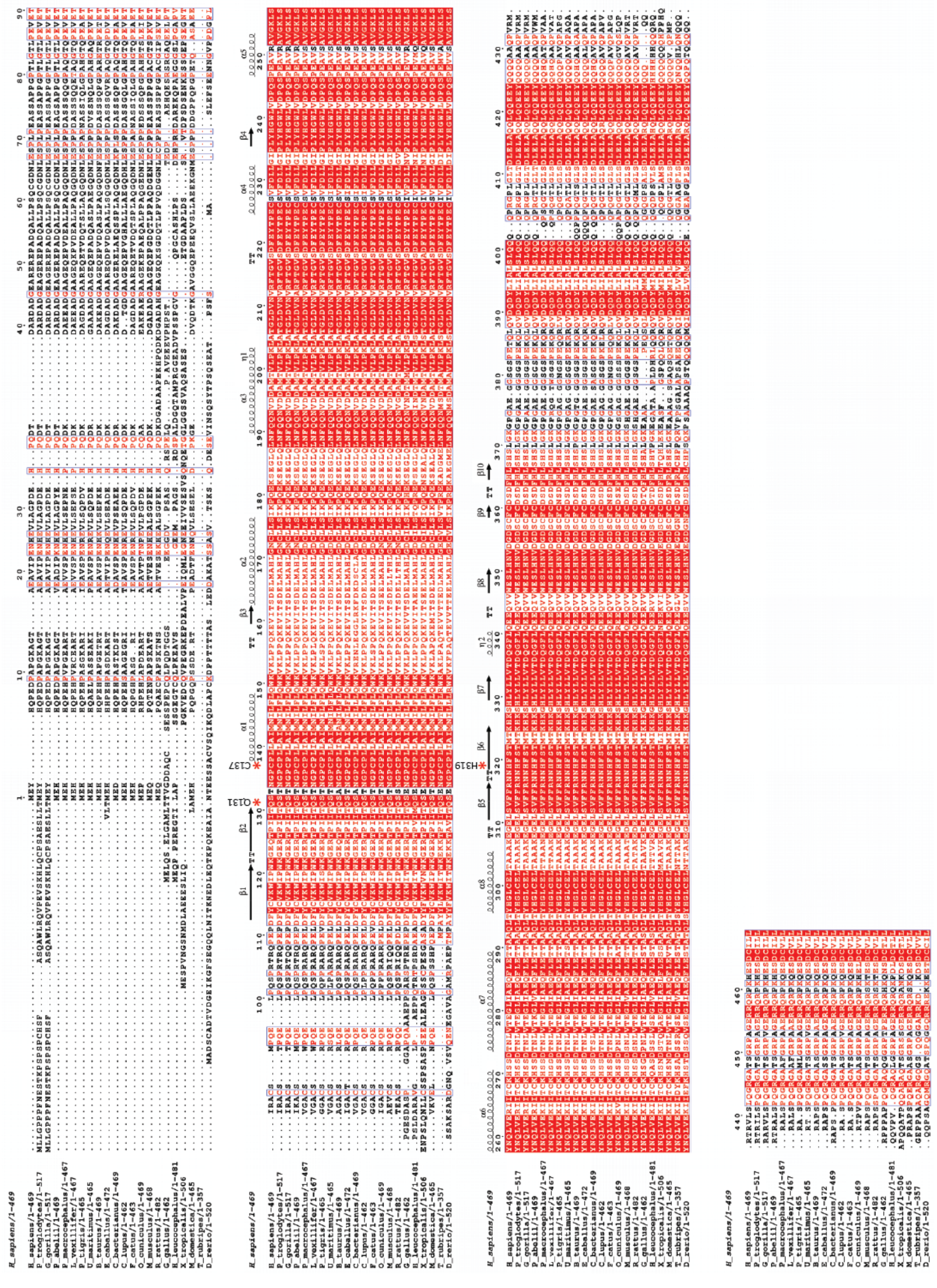

C

## Figure S1

**D**

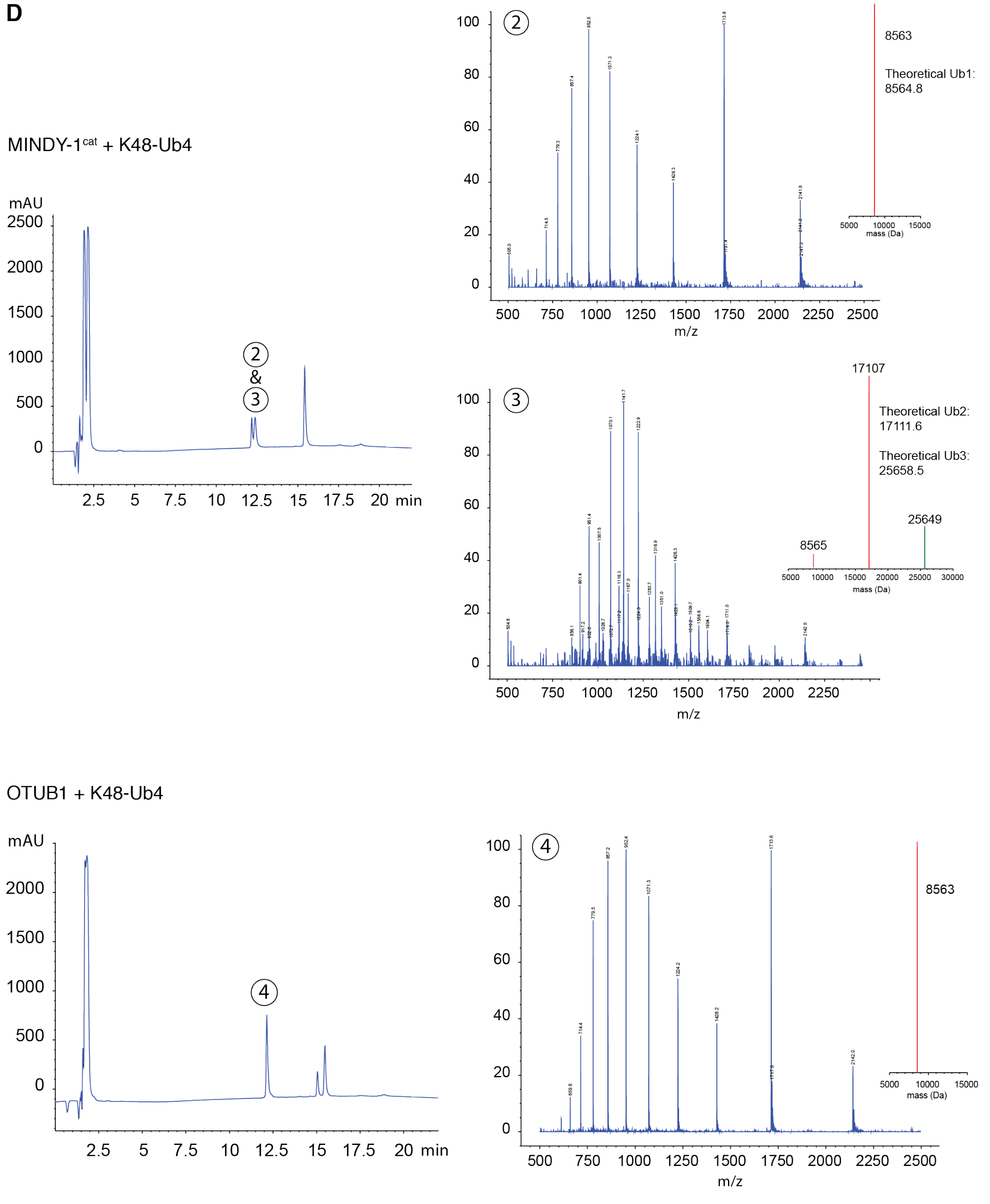
MINDY-1cat + K48-Ub4
mAU
2500
2000
1500
1000
500
0
2.5 5 7.5 10 12.5 15 17.5 20 min
2 & 3
2
100
80
60
40
20
0
500 750 1000 1250 1500 1750 2000 2250 2500
m/z
8563
Theoretical Ub1:
8564.8
5000 10000 15000
mass (Da)
3
17107
Theoretical Ub2:
17111.6
Theoretical Ub3:
25658.5
25649
8565
5000 10000 15000 20000 25000 30000
mass (Da)
500 750 1000 1250 1500 1750 2000 2250
m/z
OTUB1 + K48-Ub4
mAU
2000
1500
1000
500
0
2.5 5 7.5 10 12.5 15 17.5 20 min
4
100
80
60
40
20
0
8563
5000 10000 15000
mass (Da)
500 750 1000 1250 1500 1750 2000 2250 2500
m/z

**Figure S1.** FAM63A/MINDY-1 is a novel DUB containing tandem MIU motifs. Related to Figure 1.

(A) FAM63A contains motifs interacting with ubiquitin (MIU) at the C-terminus. Schematic representation of domain structure of human FAM63A is shown. Sequences of FAM63A from different organisms corresponding to human FAM63A 388-426 were aligned and the conserved residues are highlighted in purple. Consensus motif of MIU (Penengo et al., 2006) is conserved in two clusters within this 388-426 stretch, suggesting a possibility of tandem MIU.

(B) The catalytic domain of MINDY-1 maintains K48-linkage specificity even at high enzyme concentrations. 10 µM MINDY-$1^{cat}$ was incubated with 2.2 µM of tetraUb of different linkage types for the indicated period of time.

(C) The catalytic domain of MINDY-1 is conserved in evolution. Sequence alignment of MINDY-1/FAM63A from different organisms is shown. Secondary structure elements are shown for MINDY-$1^{cat}$. The catalytic residues are highlighted with asterisks. Fully conserved residues are shaded in red.

(D) MINDY-1 cleaves K48-polyUb chains at G76. DUB assay reactions of 2.2 µM K48-Ub4 and 1.6 µM MINDY-$1^{cat}$ or OTUB1 were carried out for 1 h at 30 °C. Left panel, samples at the end point reaction were injected and monitored by HPLC at 214 nm. Right panel, ESI-MS mass spectrum for products of DUB reaction. Inset shows the deconvoluted mass spectrum. The theoretical masses of Ub1, Ub2, and Ub3 are indicated. After 60 min of reaction, MINDY-$1^{cat}$ produced Ub1, Ub2, and Ub3 with observed mass close to the theoretical value, indicating that the MINDY-$1^{cat}$ cleaves K48-polyUb chains at G76. Cleavage by OTUB1 was used as a control (lower panel).

## Figure S2

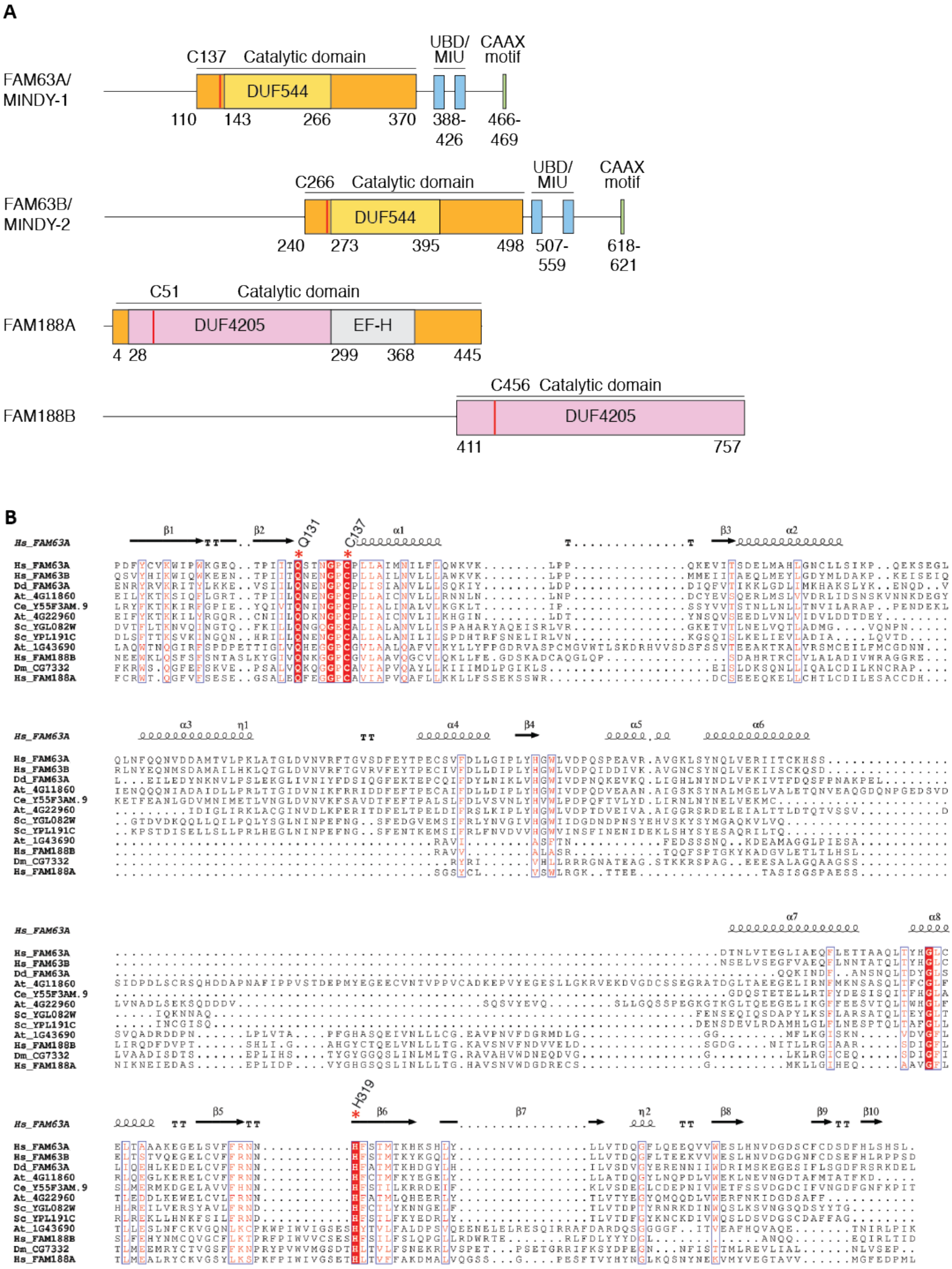
A
FAM63A/
MINDY-1
C137 Catalytic domain
DUF544
UBD/
MIU
CAAX
motif
110 143 266 370 388-426 466-469
FAM63B/
MINDY-2
C266 Catalytic domain
DUF544
UBD/
MIU
CAAX
motif
240 273 395 498 507-559 618-621
FAM188A
C51 Catalytic domain
DUF4205
EF-H
4 28 299 368 445
FAM188B
C456 Catalytic domain
DUF4205
411 757
B
Hs_FAM63A
Hs_FAM63B
Dd_FAM63A
At_4G11860
Ce_Y55F3AM.9
At_4G22960
Sc_YGL082W
Sc_YPL191C
At_1G43690
Hs_FAM188B
Dm_CG7332
Hs_FAM188A
Q131
C137
H319

## Figure S2

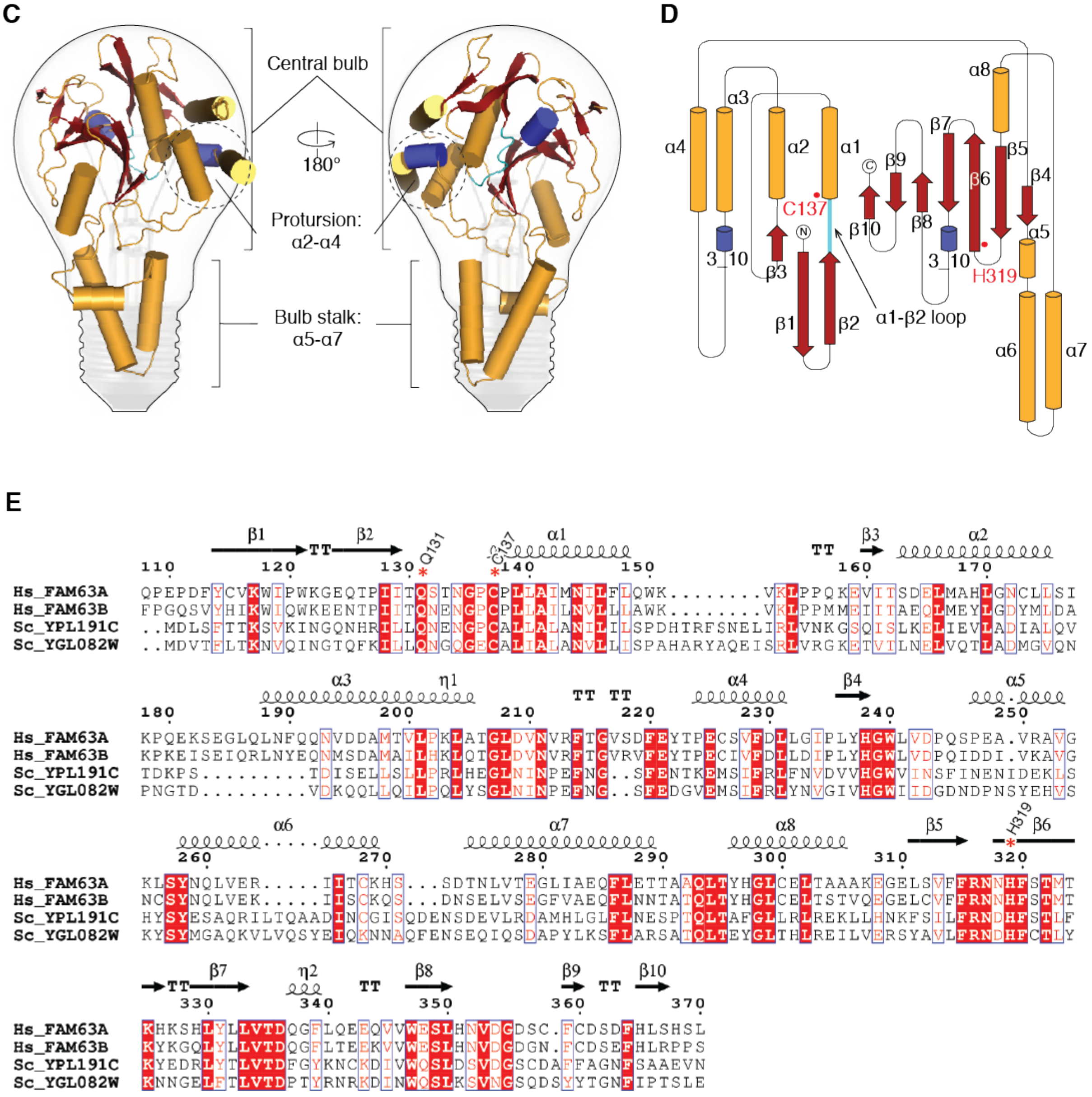


**Figure S2.** Sequence analysis of MINDY family of DUBs. Related to Figure 1.

(A) Schematic representation of domain structure of the human proteins of FAM63A/MINDY-1, FAM63B/MINDY-2, FAM188A, and FAM188B.

(B) Sequence alignment of the catalytic domain residues of MINDY family DUBs. Secondary structure elements are shown for MINDY-1$^{cat}$. The catalytic residues are highlighted with asterisks. Fully conserved residues are shaded in red. For the generation of this alignment, residues spanning the EF Hand motif (300 - 371) in FAM188A were left out. (Hs: *H. sapiens*, Dd: *D. discoideum*, At: *A.* thaliana, Ce: *C. elegans*; Sc: *S. cerevisiae*, Dm: *D. melanogaster*).

(C) The crystal structure of MINDY-1$^{cat}$ resembles a light bulb. The structure is displayed in two different orientations, rotated by 180° around the y-axis.

(D) Topology of MINDY-1$^{cat}$ structure. α-helices (orange), β-strands (red), and 3_10 helices (blue), catalytic residues, and the Cys loop connecting β2 and α1 (cyan) are indicated.

(E) Sequence alignment of human and yeast FAM63 members as in Figure S1C.

## Figure S3

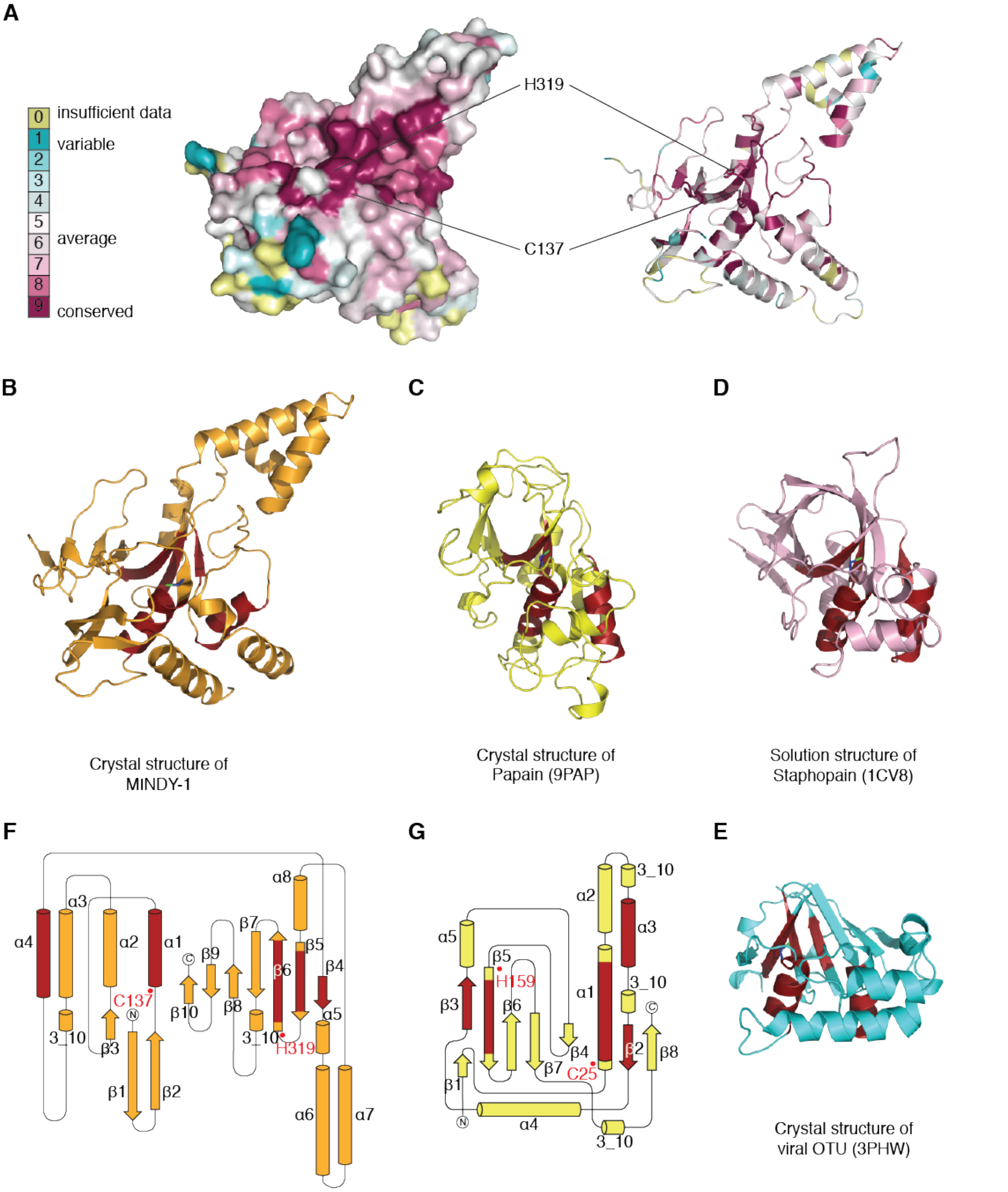


**Figure S3.** The crystal structure of MINDY-1$^{cat}$ reveals a new folding variant. Related to Figure 2.
(A) Conserved residues on the surface of MINDY-1$^{cat}$ based on sequence alignment of FAM63A and FAM63B members from different species generated with the Consurf server (http://consurf.tau.ac.il) are shown in surface and cartoon representation.
(B-E) Crystal structure of MINDY-1 (orange) (B), crystal structure of Papain (yellow) (C), solution structure of Staphopain (pink) (D), and crystal structure of viral OTU (cyan) (E) are shown in a cartoon representation. The conserved regions among these structures are highlighted in red. Multiple comparison and 3D alignment of the protein structures was performed using PDBeFold (http://www.ebi.ac.uk/msd-srv/ssm/). PDB ID: 9PAP, 1CV8, and 3PHW (Akutsu et al., 2011; Kamphuis et al., 1984). The crystal structures of Staphopain from *S. aureus* and Papain share structural identities of 14% and 9%, respectively. The structures superpose to MINDY-1$^{cat}$ structure with rmsd values of 3.5 Å and 4.1 Å over 110 and 116 aligned Cα, respectively.
(F-G) Topology of MINDY-1$^{cat}$ (F) and Papapin (G) structures with regions coloured as in (B).

## Figure S4

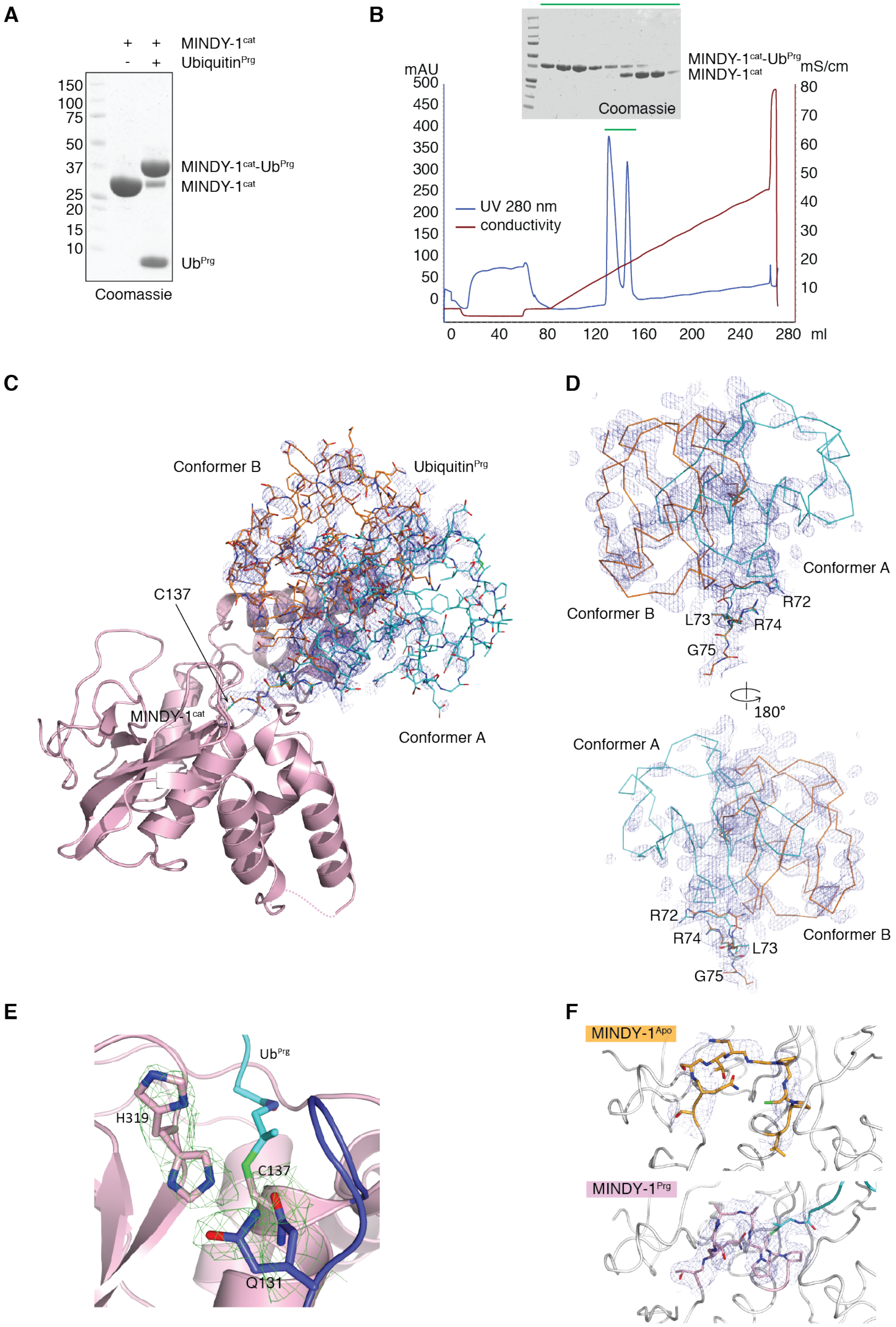
A
+ + MINDY-1cat
- + UbiquitinPrg
150
100
75
50
37
25
20
15
10
MINDY-1cat-UbPrg
MINDY-1cat
UbPrg
Coomassie
B
MINDY-1cat-UbPrg
MINDY-1cat
Coomassie
mAU
mS/cm
UV 280 nm
conductivity
0 40 80 120 160 200 240 280 ml
C
Conformer B
UbiquitinPrg
C137
MINDY-1cat
Conformer A
D
Conformer A
Conformer B
R72
L73
R74
G75
180°
E
UbPrg
H319
C137
Q131
F
MINDY-1Apo
MINDY-1Prg

**Figure S4.** Crystal structure of MINDY-$1^{cat}$~$Ub^{Prg}$. Related to Figure 3.
(A) Coomassie stained gel showing the reaction of MINDY-$1^{cat}$ with propargylated Ub.
(B) Purification of MINDY-$1^{cat}$-$Ub^{Prg}$ complex by ion exchange chromatography. SDS gel of peak fractions is shown.
(C) The crystal structure of MINDY-$1^{cat}$ (pink, cartoon representation) with electron density for the two conformers of $Ub^{Prg}$ (cyan and orange, sticks representation). The simulated annealing omit map is contoured at 0.6 σ.
(D) Electron density for Ub shown in two different orientations. The simulated annealing omit map is contoured at 0.6 σ. The C-terminal residues of the two conformers (R72-G75) align, and are represented as sticks. The electron density fit for these regions and the vinylthioether linkage is found to be relatively perfect.
(E) Close up view of the catalytic site with electron density for His319 and Gln131.
(F) Simulated annealing omit map of the Cys loop in the apo (orange) and complex (pink) structures.

## Figure S5

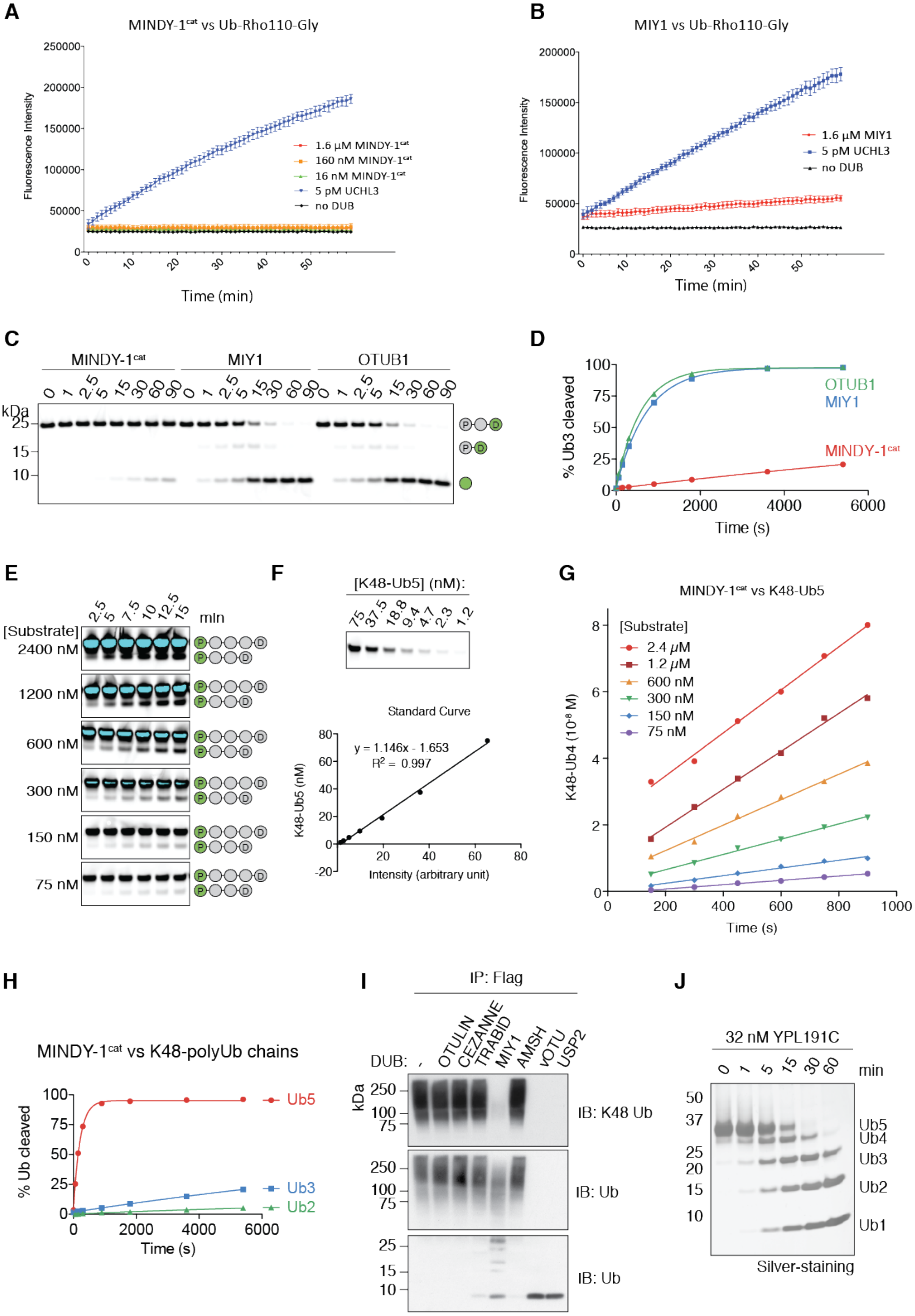
A
MINDY-1cat vs Ub-Rho110-Gly
Fluorescence Intensity
Time (min)
1.6 µM MINDY-1cat
160 nM MINDY-1cat
16 nM MINDY-1cat
5 pM UCHL3
no DUB
B
MIY1 vs Ub-Rho110-Gly
1.6 µM MIY1
5 pM UCHL3
no DUB
C
MINDY-1cat
MIY1
OTUB1
kDa
D
% Ub3 cleaved
Time (s)
OTUB1
MIY1
MINDY-1cat
E
[Substrate]
2400 nM
1200 nM
600 nM
300 nM
150 nM
75 nM
min
F
[K48-Ub5] (nM):
Standard Curve
y = 1.146x - 1.653
R2 = 0.997
K48-Ub5 (nM)
Intensity (arbitrary unit)
G
MINDY-1cat vs K48-Ub5
[Substrate]
2.4 µM
1.2 µM
600 nM
300 nM
150 nM
75 nM
K48-Ub4 (10-8 M)
Time (s)
H
MINDY-1cat vs K48-polyUb chains
% Ub cleaved
Ub5
Ub3
Ub2
Time (s)
I
IP: Flag
DUB:
OTULIN
CEZANNE
TRABID
MIY1
AMSH
vOTU
USP2
kDa
IB: K48 Ub
IB: Ub
IB: Ub
J
32 nM YPL191C
min
Ub5
Ub4
Ub3
Ub2
Ub1
Silver-staining

**Figure S5.** MINDY-1 cleaves polyUb chains in a step-wise manner. Related to Figure 4.
(A-B) MINDY-1$^{cat}$ does not hydrolyze the fluourogenic DUB substrate Ub-Rho110-G. Progress curves for 50 nM Ub-Rho110-G hydrolysis using increasing concentrations of MINDY-1$^{cat}$ (A) or MIY1 (B). UCHL3 was included as positive control.
(C) MINDY-1$^{cat}$ cleaves K48-Ub3 poorly compared to MIY1 and OTUB1. DUBs (1 μM) were incubated with 500 nM of K48-Ub3 that has been labeled with an infrared fluorescent dye at the distal Ub (green circle) for the indicated times. Fluorescence Ub was visualized using Odyssey gel scanner at 800 nm channel.
(D) Quantification of K48-Ub3 hydrolysis by MINDY-1$^{cat}$, MIY1, and OTUB1 in (C). The percentage of the total intensities of Ub4, Ub3, Ub2, and Ub1 formed is shown on the y-axis. ($n$ = 3; means ± s.d.).
(E-G) Steady state kinetics of MINDY-1$^{cat}$ for K48-Ub5. K48-Ub5 chains that have been labeled with an infrared fluorescent dye at the proximal Ub (green circle) at different concentrations were hydrolyzed by a fixed concentration of MINDY-1$^{cat}$ (15 nM) over the indicated times (E). Since the fluorescence label is only on one Ub molecule (proximal), the intensity on K48-Ub5 will be the same number of molecules of K48-Ub4, where distal Ub has been cleaved by MINDY-1$^{cat}$. Therefore, the standard curve of K48-Ub5 can be used to convert the intensity of K48-Ub4 in (E) to a molar concentration (F). The K48-Ub4 produced is plotted against time. Data was fitted to linear regression curve, where the slope is the initial velocity, $V_0$ (G). Figures S5E-S5G are representative data from one of the replicates that were used to derive Michaelis-Menten kinetics in Figure 4F.
(H) MINDY-1$^{cat}$ prefers to cleave long K48-polyUb chains. The cleavage percentage of K48-Ub2 (green), K48-Ub3 (blue), and K48-Ub5 (red) by MINDY-1$^{cat}$ over time was plotted in the same graph using the data from Figure 4C, S5D, and 4E, respectively.
(I) MINDY-1 captures K48-linked polyUb chains that contain other linkages. To analyze polyUb captured by the catalytic domain, Flag pull-downs from extracts of HEK293 cells inducibly-expressing 3xFlag-MINDY-1 C137A were incubated with a panel of linkage specific DUBs. The DUBs used and their linkage preferences are indicated in brackets: OTULIN (M1), CEZANNE (K11), TRABID (K29 and K33), MIY1 (K48), AMSH (K63), vOTU (all but M1, K27, and K29), and USP2 (all). K48 polyubiquitin chains are completely cleaved by MIY1 treatment, but are not affected by any of the other DUBs. However, only partial polyUb chains detected by anti-Ub antibody are reduced with MIY1 treatment. The remaining Ub may be a result of monoUb remnant on proteins or polyUb chains of linkage types other than K48 present as part of heterotypic chains. The release of lower molecular weight chains (Ub2-Ub5) upon MIY1 treatment suggests the presence of heterotypic chains.
(J) MIY1/YPL191C is an endo-DUB. Time course analysis of 3.5 μM pentaUb cleavage by 32 nM MIY1/YPL191C shows formation of cleavage products of all chain lengths (Ub4, Ub3, Ub2 and Ub1) at the earliest time points.

**Supplemental Tables:**

**Table S1:** cDNA constructs used in this study

| Protein | Expressed protein | Tag Cleaved | Vector type | Plasmid | DU number |
|---|---|---|---|---|---|
| MINDY-1 MIU | GST-Halo-MINDY-1 388-426 | Yes | Bacterial | pGEX6P1 | 47443 |
| MINDY-1$^{FL}$/ FAM63A$^{FL}$ | GST-MINDY-1 1-469 | Yes | Bacterial | pGEX6P1 | 49563 |
| FAM63B$^{FL}$ | GST-MINDY-1 1-621 | Yes | Bacterial | pGEX6P1 | 46765 |
| MIY1/YPL191C$^{FL}$ | GST-YPL191C 1-360 | Yes | Bacterial | pGEX6P1 | 47420 |
| YGL082W$^{FL}$ | GST-YPL191C 1-381 | Yes | Bacterial | pGEX6P1 | 47391 |
| FAM188A$^{FL}$ | GST-YPL191C 1-445 | Yes | Bacterial | pGEX6P1 | 47870 |
| MINDY-1$^{cat}$/ FAM63A$^{cat}$ | GST-MINDY-1 110-380 | Yes | Bacterial | pGEX6P1 | 47257 |
| 3xFlag-MINDY-1 | 3xFlag-MINDY-1 | No | Mammalian | pcDNA5 FRT/TO | 49562 |
| 3xFlag-MINDY-1 MIU* | 3xFlag-MINDY-1 L415A/A416G | No | Mammalian | pcDNA5 FRT/TO | 47510 |
| 3xFlag-MINDY-1 C137A | 3xFlag-MINDY-1 C137A | No | Mammalian | pcDNA5 FRT/TO | 47438 |
| Ubiquitin | Ub-Intein-CBD 1-75 | Yes | Bacterial | pTXB1 | 24149 |
| Ubiquitin G75A/G76A | Ubiquitin G75A/G76A | n.a. | Bacterial | pET24 | 49488 |
| Cys-Ubiquitin K48R | GST-Cys-Ubiquitin K48R | Yes | Bacterial | pGEX6P1 | 47729 |
| Cys-Ubiquitin 1-75 | GST-Cys-Ubiquitin 1-75 | Yes | Bacterial | pGEX6P1 | 47779 |
| Ubiquitin G75A/G76A | Ubiquitin G75A/G76A | n.a. | Bacterial | pET24 | 49488 |
| Cys-Ubiquitin K48R | GST-Cys-Ubiquitin K48R | Yes | Bacterial | pGEX6P1 | 47729 |
| Cys-Ubiquitin 1-75 | GST-Cys-Ubiquitin 1-75 | Yes | Bacterial | pGEX6P1 | 47779 |
| MINDY-1$^{cat}$ C137A | GST-MINDY-1$^{cat}$ 110-384 C137A | Yes | Bacterial | pGEX6P1 | 47419 |
| MINDY-1$^{cat}$ H319A | GST-MINDY-1$^{cat}$ 110-384 H319A | Yes | Bacterial | pGEX6P1 | 47669 |
| MINDY-1$^{cat}$ Q131A | GST-MINDY-1$^{cat}$ 10-384 Q131A | Yes | Bacterial | pGEX6P1 | 47783 |
| MINDY-1$^{cat}$ Q131E | GST-MINDY-1$^{cat}$ 110-384 Q131E | Yes | Bacterial | pGEX6P1 | 47832 |
| MINDY-1$^{cat}$ V210A | GST-MINDY-1$^{cat}$ 110-384 V210A | Yes | Bacterial | pGEX6P1 | 55059 |
| MINDY-1 W240A | GST-MINDY-1$^{cat}$ 110-384 W240A | Yes | Bacterial | pGEX6P1 | 47817 |
| MINDY-1$^{cat}$ Y258A | GST-MINDY-1$^{cat}$ 110-384 Y258A | Yes | Bacterial | pGEX6P1 | 47818 |
| MINDY-1$^{cat}$ F315A | GST-MINDY-1$^{cat}$ 110-384 F315A | Yes | Bacterial | pGEX6P1 | 47819 |
| MINDY-1$^{cat}$ E263A | GST-MINDY-1$^{cat}$ 110-384 E263A | Yes | Bacterial | pGEX6P1 | 47663 |
| MINDY-1$^{cat}$ E263R | GST-MINDY-1$^{cat}$ 110-384 E263R | Yes | Bacterial | pGEX6P1 | 47683 |
| MINDY-1$^{cat}$ D209A | GST-MINDY-1$^{cat}$ 110-384 D209A | Yes | Bacterial | pGEX6P1 | 47664 |
| MINDY-1$^{cat}$ D209A E263A | GST-MINDY-1$^{cat}$ 110-384 D209A E263A | Yes | Bacterial | pGEX6P1 | 47697 |

## Supplemental Materials and Methods

### Protein expression and purification

Recombinant GST-fusion proteins were expressed in *E. coli* strain BL21. Cultures were grown in 2xTY media containing 50 µg/ml ampicillin to an $OD_{600}$ of 0.6-0.8. Protein expression was induced with 300 µM IPTG and cultures grown overnight at 18 °C. Harvested cells were resuspended in GST-Lysis Buffer (50 mM Tris-HCl pH 7.5, 300 mM NaCl, 10% glycerol, 0.075% 2-mercaptoethanol, 1 mM benzamidine, 1 mM PMSF, and complete protease inhibitor cocktail (Roche)) and lysed by sonication. Bacterial lysate was clarified by centrifugation at 30,000 x g for 30 min and subsequently incubated with Glutathione Sepharose 4B resin (GE Healthcare) for 2-4 h at 4 °C. The resin bound proteins were washed extensively with high salt buffer (25 mM Tris pH 7.5, 500 mM NaCl, and 10 mM DTT) and low salt buffer (25 mM Tris-HCl pH 7.5, 150 mM NaCl, 10% glycerol, and 1 mM DTT). The GST tag was removed by overnight incubation with 3C protease at 4 °C.

Proteins were further purified by anion exchange chromatography (Resource Q, GE Healthcare Life Sciences) and eluted in a gradient with buffer Q (50 mM Tris-HCl pH 8.5, 1 M NaCl and 2 mM DTT), followed by size exclusion chromatography (Superdex 75 16/60, GE Healthcare Life Sciences) in buffer G (50 mM Tris-HCl, 150 mM NaCl, 10 mM DTT). The purified proteins were concentrated, flash frozen in liquid nitrogen and stored at -80 °C.

### Ubiquitin binding domain (UBD) linkage specificity analysis

Halo-tagged MINDY-1 (388-426) (10.5 nmol) was incubated with 100 µl of the HaloLink resin (Promega) in 500 µl of the coupling buffer (50 mM Tris-HCl pH 7.5, 150 mM NaCl, 0.05% NP-40, 1 mM DTT) for 3 h at 4 °C. The UBD linkage specificity analysis was carried out by incubating 10 µl of the coupled Halo-MINDY-1 resin (388-426) with 58.5 nM of tetraUb of the indicated linkages in 500 µl of pull down buffer (50 mM Tris-HCl pH 7.5, 150 mM NaCl, 0.1% NP-40, 1 mM DTT, 0.5 mg/ml BSA) for 2 h at 4 °C. The resin was washed two times with the wash buffer (50 mM Tris-HCl pH 7.5, 250 mM NaCl, 0.2% NP-40, 1 mM DTT) and once with the coupling buffer. Captured tetraUb chains were eluted by adding LDS buffer, separated on 4-12% SDS-PAGE gel (Life Technology), and visualized by silver staining using Pierce Silver stain kit (ThermoFischer).

### Deubiquitylation assays

DUBs were diluted in 50 mM Tris-HCl pH 7.5, 50 mM NaCl, 10 mM DTT and incubated at 24 °C for 10 min. The DUB assays were subsequently carried out where 2.2 µM of tetraUb of different linkage types were incubated with 1.6 µM DUB in 50 mM Tris-HCl pH 7.5, 50 mM NaCl, 10 mM DTT in a reaction volume of 10 µl. For DUB assays in Fig. 4A, 160 nM YPL191C/MIY1 was used. For DUB assays comparing activity of MINDY-1 at cleaving K48 chains, the following concentrations of Ub chains were used: 1.9 µM K48-Ub3 or 2.2 µM K48-Ub4, 3.5 µM K48-Ub5, or 3.5 µg of K48-Ub5-n. The reactions were incubated at 30 °C and stopped at different time points by adding LDS buffer. The samples were separated on 4-12% SDS-PAGE gel (Life Technology) and silver stained using Pierce Silver stain kit (ThermoFischer).

### Preparation and purification of MINDY-1$^{cat}$~Ub$^{Prg}$ complexes

Ubiquitin thioester was generated following cleavage of Ub-intein as described previously (Borodovsky et al., 2002). The Ub-thioester was dialyzed into HEPES buffer, pH 8.0 and 250 mM Propargylamine (Sigma) was added to the dialyzed Ub-thioester and the reaction incubated for 4 hours at 20 °C in the dark. Unreacted propargylamine was separated from propargylated Ub by size exclusion chromatography. To obtain MINDY-1$^{cat}$~Ub$^{Prg}$ complexes, purified MINDY-1$^{cat}$ was incubated with molar excess of propargylated Ub for 30 min at room temperature followed by overnight incubation at 4 °C (**Figure S4A**). For crystallization, the covalent complex was purified by anion exchange chromatography (RESOURCE Q) (**Figure S4B**) followed by SEC in buffer G. The purified complex was concentrated to 11.6 mg/ml and used for crystallization.

### Crystallization, data collection and determination of MINDY-1$^{cat}$ structure

Initial hits of crystals were obtained by screening using sitting-drop vapor diffusion method. For MINDY-1$^{cat}$, native crystals were grown from hanging drops containing an equal volume of protein (10mg/ml) and mother liquor containing 100 mM HEPES-Na pH 7.5, 200 mM NaCl, and 25% PEG4000. For anomalous phasing, diffraction quality crystals were obtained with macro-seeding. Native crystals were macroseeded into drops containing 1mM ethylmercuric phosphate (EMP). The crystals were flash frozen in cryoprotectant containing 100mM HEPES-Na pH 7.3, 200 mM NaCl, 35% PEG 4000 and 5% PEG 400. Interestingly, the presence of the mercury derivative also resulted in better diffraction of crystals. The data collection for anomalous and native data sets were done at Diamond light source (I02) and ID23-1 beamline, European Synchrotron Radiation Facility (ESRF) respectively.

The structure of MINDY-1$^{cat}$ was determined by SAD phasing using data collected from crystals grown with mercury derivative. The anomalous data were processed using XDS (Kabsch, 2010) and then scaled using AIMLESS (Evans and Murshudov, 2013) within XIA2 (Winter et al., 2013). The structure was solved using the

SAS protocol of Auto-Rickshaw: the EMBL-Hamburg automated crystal structure determination platform (Panjikar et al., 2005). The partially built model obtained was further submitted to the MRSAD pipeline. The complete model for the interpretable density was built manually in Coot (Emsley et al., 2010). The native structure was determined by molecular replacement using PHASER with the partially built structure as a search model (McCoy et al., 2007). Iterative rounds of model building and refinement were performed with coot and Refmac5 (Murshudov et al., 1997). The data collection and refinement statistics for MINDY-1$^{cat}$ structure are shown in Table 1. All the figures were made using PyMOL (http://pymol.org).

**Crystallization, data collection and structure determination of MINDY-1$^{cat}$~Ub**

Crystals for MINDY-1$^{cat}$~Ub (11.6 mg/ml) grew in hanging drops from conditions with 100mM MES pH 6.5, 10% Dioxane and 1.6 M ammonium sulphate. The crystals were directly harvested and vitrified. The MINDY-1$^{cat}$~Ub crystals diffracted to 2.5 Å at ID29 beamline, ESRF, France. The data was processed using XDS and scaled and merged using AIMLESS. The structure of the complex was solved by molecular replacement using MINDY-1$^{cat}$ and Ub (PDB ID: 1UBQ) as search models. In the MINDY-1$^{cat}$~Ub structure, clear electron density is visible for MINDY-1$^{cat}$. In contrast, electron density appears to be fractured or disordered in stretches for Ub. When refined at lower resolution two alternate conformations of Ub could be docked into the electron density. The structure was finally refined at 2.65 Å with occupancy of the two conformers set to ~0.5 each. The final data collection and refinement statistics for the MINDY-1$^{cat}$~Ub structure is shown in Table 1.

**Ubiquitin-Rhodamine110-Glycine hydrolysis assay**

Ub-Rho110-G was expressed and purified as described previously (Hassiepen et al., 2007; Ritorto et al., 2014). In the assay, 50 nM Ub-Rho110-G was incubated with 5 pM – 1.6 μM DUB in a final volume of 20 μl reaction buffer (50 mM Tris-HCl pH 7.5, 50 mM NaCl, 10 mM DTT, 0.1 mg/ml BSA). Samples were prepared in triplicates in 96-well plates and analysed using PHERAstar FS (BMG Labtech) Excitation/Emission 485/520 for 60 cycles with 60 s of interval.

**Generation of fluorescently labeled K48-polyUb chains**

*Assembly of K48-Ub2 that contains Cys residue at the distal Ub (K48-Ub2 [Cys-$Ub_{distal}$])*

K48-Ub2 [Cys-$Ub_{distal}$] was assembled enzymatically using Ub G75A/G76A as the Ub acceptor and Ub K48R with Cys residue upstream of M1 (Cys-Ub K48R) as the Ub donor. The assembly reaction was carried out for 1 h at 30 °C in 1 ml reaction containing 1 mM Ub G75A/G76A, 1 mM Cys-Ub K48R, 0.5 μM UBE1, 15 μM UBE2R1, 10 mM ATP, 50 mM Tris-HCl (pH 7.5), 10 mM MgCl2, and 0.6 mM DTT. K48-Ub2 [Cys-$Ub_{distal}$] was purified as described previously (Kristariyanto et al., 2015).

*Assembly of K48-Ub3 that contains Cys residue at the distal Ub (K48-Ub3 [Cys-$Ub_{distal}$])*

K48-Ub3 [Cys-$Ub_{distal}$] was assembled by capping a pre-assembled K48-Ub2 by Cys-Ub K48R (donor Ub). The assembly reaction was carried out for 2 h at 30 °C in 1 ml reaction containing 250 μM K48-Ub2, 1.25 mM Cys-Ub K48R, 0.5 μM UBE1, 15 μM UBE2R1, 10 mM ATP, 50 mM Tris-HCl (pH 7.5), 10 mM MgCl2, and 0.6 mM DTT. K48-Ub3 [Cys-$Ub_{distal}$] was purified as described previously (Kristariyanto et al., 2015).

*Assembly of K48-Ub5 that contains Cys residue at the proximal Ub (K48-Ub5 [Cys-$Ub_{prox}$])*

K48-Ub5 [Cys-$Ub_{prox}$] was assembled by extending Ub 1-75 (Ub acceptor), which contains Cys residue upstream of M1 (Cys-Ub 1-75), with a pre-assembled K48-Ub2. The assembly reaction was carried out for 1.5 h at 30 °C in 1 ml reaction containing 250 μM K48-Ub2, 1.25 mM Cys-Ub 1-75 (acceptor/proximal Ub), 0.5 μM UBE1, 15 μM UBE2R1, 10 mM ATP, 50 mM Tris-HCl (pH 7.5), 10 mM MgCl2, and 0.6 mM DTT. The reaction produced K48-Ub3 [Cys-$Ub_{prox}$] and smaller quantity of K48-Ub5 [Cys-$Ub_{prox}$]. K48-Ub5 [Cys-$Ub_{prox}$] was purified as described previously (Kristariyanto et al., 2015).

*Fluorescence labeling*

The infrared dye, IRDye® 800CW Maleimide, was purchased from LI-COR. IRDye 800CW was conjugated to Cys residue of the Cys-Ub mutant using standard thiol-maleimide conjugation procedure. Briefly, K48-polyUb chains were diluted in 20 mM Tris-HCl pH 7.5, 500 μM TCEP to a concentration of 50-100 μM. The protein samples were purged with argon and kept at room temperature for 30 min. Four-fold excess of IRDye 800CW was added to the reaction mix. The reaction was purged with argon and incubated at room temperature for 2 h. To stop the reaction, excess amount of 2-mercaptoethanol was added. IRDye 800CW-labeled K48-chains were purified using PD-10 desalting column (GE Healthcare).

**Quantitative fluorescence deubiquitylation assays**

DUB assay reactions were performed at 30 °C by adding 10 µl pre-activated DUB and 10 µl of fluorescence-labeled K48-polyUb chains. The reaction mixtures contained 1 µM DUB, 500 nM fluorescence-labeled K48-polyUb chains, 50 mM Tris-HCl pH 7.5, 50 mM NaCl, 10 mM DTT, and 0.25 mg/ml BSA. At the indicated time points, 2.5 µl of the samples was transferred to 7.5 µl LDS sample buffer to quench the reaction. Samples were separated on 4-12% SDS-gel and visualized using Odyssey imaging system (LI-COR) at 800 nm channel. Intensities of K48-chains were quantified using Image Studio Lite (LI-COR).

**Steady-state deubiquitylation assay**

Steady-state enzyme kinetics of MINDY-$1^{cat}$ in hydrolyzing K48-Ub5 were measured in reactions containing 15 nM MINDY-$1^{cat}$ and 75, 150, 300, 600, 1200, or 2400 nM fluorescence-labeled K48-Ub5 in 50 mM Tris-HCl pH7.5, 50 mM NaCl, 10 mM DTT, and 0.25 mg/ml BSA at 30 °C. Aliquots of 2.5 µl were mixed with 7.5 µl LDS sample buffer at 2.5, 5, 7.5, 10, 12.5, and 15 min. Samples were separated and the formation of K48-Ub4 was analyzed and quantified as described above (**Figure S5E**). To convert the quantified intensity of the formed K48-Ub4 to molar concentration, a standard curve of 1.17 to 75 nM IR-K48-Ub5 was made (**Figure S5F**). Since the fluorescence label is only on one Ub molecule (proximal), the intensity of K48-Ub5 will translate to the same number of molecules of K48-Ub4, or equal molar concentrations. The amount of K48-Ub4 formed was plotted against time and the data was fitted to a linear regression curve, where slope is the initial velocity, $V_0$ ($M.s^{-1}$) (Figure S5G). The initial velocity values were then plotted against substrate concentration and the data was fitted to the Michaelis-Menten equation:

$$V_0 = \frac{V_{max}[\mathrm{S}]}{K_m + [\mathrm{S}]} = \frac{k_{cat}[\mathrm{E}][\mathrm{S}]}{K_m + [\mathrm{S}]}$$

where $V_0$ is the initial velocity, $V_{max}$ is the maximum velocity, [S] is substrate concentration, $K_m$ is the Michaelis constant, and $k_{cat}$ is the turnover rate. Data fitting was carried out using GraphPad Prism 5 software.

**Cell culture and transfection**

Flp-In T-REx HEK293 cells were cultured in Dulbecco's modified Eagle's medium (DMEM) containing 10% fetal bovine serum (FBS), 2 mM L-glutamine, 100 units/ml penicillin, 100 µg/ml streptomycin, 100 µg/ml Zeocin and 15 µg/ml Blasticidin. Tetracycline-inducible stable cell lines were generated by transfecting Flp-In T-REx HEK293 cells with the indicated constructs. Transfection was performed by GeneJuice Transfection Reagent (Novagen). Protein expression was induced by addition of fresh tissue culture medium containing tetracycline at a final concentration of 1 µg/ml for 24 h.

**Flag immunoprecipitations**

Cells were lysed in lysis buffer (1% Triton-X, 50 mM Tris-HCl pH 7.5, 0.1 mM EDTA, 0.1 mM EGTA, 10 mM sodium glycerol 2-phosphate, 50 mM sodium fluoride, 5 mM sodium pyrophosphate, 270 mM sucrose, 1 mM sodium orthovanadate, 25 mM iodoacetamide, 1 mM AEBSF, 0.02% benzonase) supplemented with protease inhibitor cocktail. The cell lysates were incubated with Anti-Flag M2 agarose resins (Sigma Aldrich) for 2 hours at 4 °C. The resins were washed 3 times with lysis buffer and the captured proteins were analyzed by immunoblotting. Where indicated, the Flag immunoprecipitated materials were treated with a panel of linkage specific DUBs as described previously (Kristariyanto et al., 2015).

**Supplemental References:**

Akutsu, M., Ye, Y., Virdee, S., Chin, J.W., and Komander, D. (2011). Molecular basis for ubiquitin and ISG15 cross-reactivity in viral ovarian tumor domains. Proc. Natl. Acad. Sci. USA *108*, 2228–2233.

Borodovsky, A., Ovaa, H., Kolli, N., Gan-Erdene, T., Wilkinson, K.D., Ploegh, H.L., and Kessler, B.M. (2002). Chemistry-based functional proteomics reveals novel members of the deubiquitinating enzyme family. Chem. Biol. *9*, 1149–1159.

Emsley, P., Lohkamp, B., Scott, W.G., and Cowtan, K. (2010). Features and development of Coot. Acta Crystallogr. D Biol. Crystallogr. *66*, 486–501.

Evans, P.R., and Murshudov, G.N. (2013). How good are my data and what is the resolution? Acta Crystallogr. D Biol. Crystallogr. *69*, 1204–1214.

Hassiepen, U., Eidhoff, U., Meder, G., Bulber, J.-F., Hein, A., Bodendorf, U., Lorthiois, E., and Martoglio, B. (2007). A sensitive fluorescence intensity assay for deubiquitinating proteases using ubiquitin-rhodamine110-glycine as substrate. Anal. Biochem. *371*, 201–207.

Kabsch, W. (2010). XDS. Acta Crystallogr. D Biol. Crystallogr. *66*, 125–132.

Kamphuis, I.G., Kalk, K.H., Swarte, M.B., and Drenth, J. (1984). Structure of papain refined at 1.65 A resolution. J. Mol. Biol. *179*, 233–256.

Kristariyanto, Y.A., Rehman, S.A.A., Campbell, D.G., Morrice, N.A., Johnson, C., Toth, R., and Kulathu, Y. (2015). K29-Selective Ubiquitin Binding Domain Reveals Structural Basis of Specificity and Heterotypic Nature of K29 Polyubiquitin. Mol. Cell *58*, 83–94.

McCoy, A.J., Grosse Kunstleve, R.W., Adams, P.D., Winn, M.D., Storoni, L.C., and Read, R.J. (2007). Phaser crystallographic software. J. Appl. Crystallogr. *40*, 658–674.

Murshudov, G.N., Vagin, A.A., and Dodson, E.J. (1997). Refinement of macromolecular structures by the maximum-likelihood method. Acta Crystallogr. D Biol. Crystallogr. *53*, 240–255.

Panjikar, S., Parthasarathy, V., Lamzin, V.S., Weiss, M.S., and Tucker, P.A. (2005). Auto-rickshaw: an automated crystal structure determination platform as an efficient tool for the validation of an X-ray diffraction experiment. Acta Crystallogr. D Biol. Crystallogr. *61*, 449–457.

Penengo, L., Mapelli, M., Murachelli, A.G., Confalonieri, S., Magri, L., Musacchio, A., Di Fiore, P.P., Polo, S., and Schneider, T.R. (2006). Crystal structure of the ubiquitin binding domains of rabex-5 reveals two modes of interaction with ubiquitin. Cell *124*, 1183–1195.

Ritorto, M.S., Ewan, R., Perez-Oliva, A.B., Knebel, A., Buhrlage, S.J., Wightman, M., Kelly, S.M., Wood, N.T., Virdee, S., Gray, N.S., Morrice, N.A., Alessi, D.R., and Trost, M. (2014). Screening of DUB activity and specificity by MALDI-TOF mass spectrometry. Nat. Commun. *5*, 4763.

Winter, G., Lobley, C.M.C., and Prince, S.M. (2013). Decision making in xia2. Acta Crystallogr. D Biol. Crystallogr. *69*, 1260–1273.
