# Supplementary material for: MINDY-1 Is a Member of an Evolutionarily Conserved and Structurally Distinct New Family of Deubiquitinating Enzymes
Source: Mol Cell. 2016 Jul 7;63(1):146–55. doi: 10.1016/j.molcel.2016.05.009 (PMC4942677; doi:10.1016/j.molcel.2016.05.009)
Supplement: Document S2. Article plus Supplemental Information [file mmc2.pdf]

Short Article

Molecular Cell

MINDY-1 Is a Member of an Evolutionarily Conserved and Structurally Distinct New Family of Deubiquitinating Enzymes

Graphical Abstract

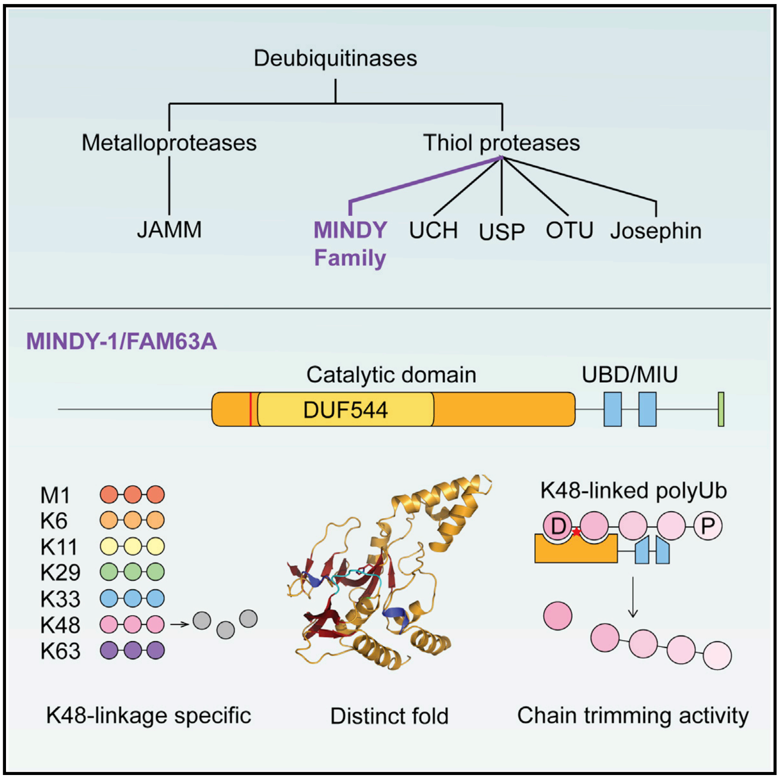

Authors

Syed Arif Abdul Rehman, Yosua Adi Kristariyanto, Soo-Youn Choi, ..., Karim Labib, Kay Hofmann, Yogesh Kulathu

Correspondence

ykulathu@dundee.ac.uk

In Brief

Abdul Rehman et al. discover a new family of deubiquitinating enzymes called MINDY. This structurally distinct family of DUBs is highly selective at cleaving K48-linked polyubiquitin chains.

Highlights

- MINDY is a new family of DUBs consisting of FAM63A, FAM63B, FAM188A, and FAM188B
- MINDY DUBs are highly selective at cleaving K48-linked polyubiquitin
- Catalytic domain of MINDY-1 adopts a distinct fold with no homology to any known DUB
- Human MINDY-1 trims ubiquitin chains from the distal end

Accession Numbers

5JKN
5JQS

Abdul Rehman et al., 2016, Molecular Cell *63*, 146–155
July 7, 2016 © 2016 The Author(s). Published by Elsevier Inc.
http://dx.doi.org/10.1016/j.molcel.2016.05.009

Molecular Cell
Short Article

MINDY-1 Is a Member of an Evolutionarily Conserved and Structurally Distinct New Family of Deubiquitinating Enzymes

Syed Arif Abdul Rehman,[1,3] Yosua Adi Kristariyanto,[1,3] Soo-Youn Choi,[1,3] Pedro Junior Nkosi,[1] Simone Weidlich,[1] Karim Labib,[1] Kay Hofmann,[2] and Yogesh Kulathu[1,*]
[1]MRC Protein Phosphorylation & Ubiquitylation Unit, School of Life Sciences, University of Dundee, Dow Street, Dundee DD1 5EH, UK
[2]Institute for Genetics, University of Cologne, Zülpicher Straße 47a, 50674 Cologne, Germany
[3]Co-first author
*Correspondence: ykulathu@dundee.ac.uk
http://dx.doi.org/10.1016/j.molcel.2016.05.009

SUMMARY

Deubiquitinating enzymes (DUBs) remove ubiquitin (Ub) from Ub-conjugated substrates to regulate the functional outcome of ubiquitylation. Here we report the discovery of a new family of DUBs, which we have named MINDY (motif interacting with Ub-containing novel DUB family). Found in all eukaryotes, MINDY-family DUBs are highly selective at cleaving K48-linked polyUb, a signal that targets proteins for degradation. We identify the catalytic activity to be encoded within a previously unannotated domain, the crystal structure of which reveals a distinct protein fold with no homology to any of the known DUBs. The crystal structure of MINDY-1 (also known as FAM63A) in complex with propargylated Ub reveals conformational changes that realign the active site for catalysis. MINDY-1 prefers cleaving long polyUb chains and works by trimming chains from the distal end. Collectively, our results reveal a new family of DUBs that may have specialized roles in regulating proteostasis.

INTRODUCTION

Ubiquitylation is a post-translational modification (PTM) that regulates almost every facet of eukaryotic biology. The functional outcome of protein ubiquitylation is determined by the type of modification (monoubiquitin or polyubiquitin) and also the linkage type within the ubiquitin (Ub) chain (Kulathu and Komander, 2012). Ub binding domain (UBD)-containing proteins bind to different Ub modifications and link Ub signals to downstream signaling (Di Fiore et al., 2003). The outcome of Ub signals is regulated by deubiquitinating enzymes (DUBs), which are proteases that remove Ub from modified substrates (Reyes-Turcu et al., 2009). DUBs are therefore important regulators of the Ub system and regulate a plethora of cellular processes, including protein turnover, protein sorting, and trafficking (Clague et al., 2012; MacGurn et al., 2012). Indeed, deregulated DUB activity may promote human disease, and hence DUBs are being actively explored as potential drug targets (Heideker and Wertz, 2015; King and Finley, 2014). There are approximately 100 DUBs encoded in the human genome that can be classified into five families on the basis of the mechanism of catalysis (Clague et al., 2013; Nijman et al., 2005). Of the five families, four are thiol proteases, while the fifth includes metalloproteases (Reyes-Turcu et al., 2009).

One major function of ubiquitylation is in protein degradation, a precise proteolytic process essential for the turnover of many proteins and also for removing damaged proteins (Eletr and Wilkinson, 2014). Modification of proteins with K48-linked polyUb chains, the most abundant linkage type detected in cells, is the canonical signal that marks proteins for degradation by the 26S proteasome (Finley, 2009; Kulathu and Komander, 2012). Prior to degradation, proteasome-associated DUBs release Ub from substrates to recycle Ub (Inobe and Matouschek, 2014). Some DUBs can trim the K48 chains on ubiquitylated substrates to rescue them from degradation (Lam et al., 1997; Lee et al., 2010; Rumpf and Jentsch, 2006), highlighting the fine control that can be exerted by DUBs.

Here we make the surprising discovery that an uncharacterized protein, FAM63A, is a deubiquitinase that is highly selective at hydrolyzing K48-linked polyUb. We delineate this deubiquitinating activity to be encoded within a previously unannotated domain. Our structural analyses reveal that the catalytic domain of FAM63A is a distinct folding variant of the superfamily of cysteine proteases. FAM63A has no homology to any of the known DUBs, so we classify this newly discovered Ub protease to be a prototype of a new family of DUBs. Defining FAM63A as a DUB led to the identification of further members that form part of this family.

RESULTS AND DISCUSSION

In order to understand linkage selectivity in Ub signaling, we recently developed methods to assemble tetraUb chains of different linkage types that allow us to profile linkage selectivity of UBDs (Kristariyanto et al., 2015b). The motif interacting with Ub (MIU) is a small UBD consisting of a helical motif that binds to monoUb (Lee et al., 2006; Penengo et al., 2006). When investigating this class of UBDs, we identified FAM63A to contain MIU motifs with high selectivity for binding K48-linked polyUb (Figures 1A, 1B, and S1A). In addition to this K48-binding UBD, FAM63A

 © 2016 The Author(s). Published by Elsevier Inc.
This is an open access article under the CC BY license (http://creativecommons.org/licenses/by/4.0/).

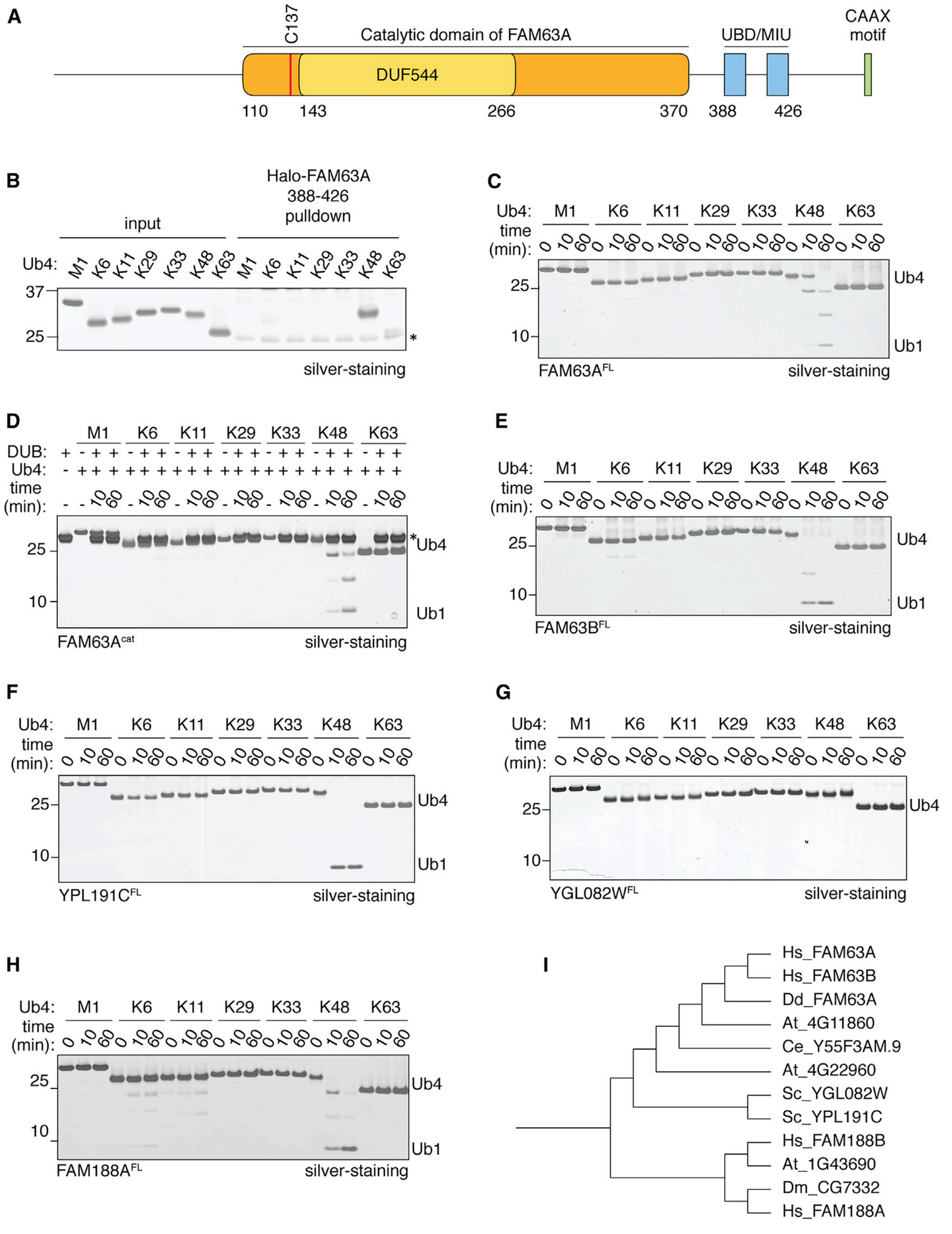

Figure 1. Identification of the MINDY Family of DUBs

(A) Schematic representation of the domain structure of human FAM63A.

(B) Halo-tagged FAM63A (388–426) coupled to HaloLink resin was incubated with tetraUb of the indicated linkage types. The captured materials were separated on 4%–12% SDS-PAGE gel and silver stained.

(C–H) DUB assays testing activity and specificity of polyUb cleavage by FAM63A (C), putative catalytic domain of FAM63A (110–384) (D) (asterisk indicates FAM63A), full-length FAM63B (E), YPL191C/MIY1 (F), YGL082W (G), and full-length human FAM188A (H); 1.6 μM of DUBs were incubated with 2.2 μM of tetraUb chains for the indicated time.

(I) Phylogenetic tree of MINDY family DUBs based on alignment of catalytic domains (Figure S2B).

See also Figure S1.

Table 1. Data Collection and Refinement Statistics

	MINDY-1^{cat} (Anomalous)	MINDY-1^{cat} (Native)	MINDY-1^{cat}~Ub (Complex)
Data Collection			
Beamline	I02, DLS	ID23-1, ESRF	ID29, ESRF
Space group	$P4_122$	$P4_122$	$P6_522$
a, b, c (Å)	100.55, 100.55, 165.64	99.67, 99.67, 165.12	82.33, 82.33, 332.46
α, β, γ (°)	90.00, 90.00, 90.00	90.00, 90.00, 90.00	90.00, 90.00, 120.00
Wavelength (Å)	1.0073	0.93927	0.97623
Resolution (Å)	63.93–3.39 (3.39–3.30)[a]	48.18–3.0 (3.18–3.0)	48.63–2.65 (2.78–2.65)
R-merge	0.35 (4.31)	0.065 (0.883)	0.089 (0.595)
I/σ(I)	9.8 (1.2)	14.4 (2.1)	17.1 (3.3)
Completeness (%)	99.7 (99.2)	99.8 (99.9)	99.9 (100.0)
Multiplicity	13.8 (13.5)	5.0 (5.2)	9.4 (10.0)
CC1/2	0.996 (0.526)	0.998 (0.866)	0.999 (0.925)
Refinement			
Resolution (Å)		48.18–3.0	48.63–2.65
No. of reflections		17,325 (2,732)	20,518 (2,621)
R_{work}/R_{free}		0.197/0.242	0.205/0.231
No. of Atoms			
Protein		2,009	3,078
Waters		0	53
Ligand/ion		13	26
B Factors (Å^2)			
Wilson B		97.80	51.5
Protein		120.74	71.55
Ligand/Ion		130.70	78.20
Water			50.64
RMSDs			
Bond length (Å)		0.007	0.008
Bond angles (°)		1.204	1.104
Ramachandran statistics (favored/allowed/outliers)		95.0/5.0/0.0	97.0/3.0/0.0

DLS, Diamond Light Source; ESRF, European Synchrotron Radiation Facility; RMSD, root-mean-square deviation.
[a]Values for the highest-resolution shell are shown in parentheses.

contains a domain of unknown function (DUF544) (Figure 1A). Given the presence of a UBD, we posited a Ub-dependent function for the associated DUF domain (Marcotte et al., 1999). Sequence conservation within the FAM63 family suggested the presence of a cysteine protease active site, and therefore we hypothesized that it may be a Ub protease. To test this hypothesis, we performed DUB assays in which we monitored in vitro cleavage of different linkages of tetraUb chains, which shows that full-length FAM63A readily cleaves K48 chains (Figure 1C). In contrast, all other chain types are intact. This revealed that FAM63A is a DUB with unique selectivity for cleaving K48-linked polyUb. To identify the catalytic domain in FAM63A, we used multiple sequence alignment and secondary structure prediction (Kelley et al., 2015), on the basis of which we predicted the middle region of FAM63A (residues 110–384), which also includes DUF544, to be the catalytic domain (Figures 1A and S1C). Indeed, this predicted domain encodes catalytic activity that maintains high specificity for K48 linkages (Figures 1D, S1B, and S1D).

Human FAM63B is another DUF544-containing protein that shares sequence similarity and domain organization with FAM63A (Figure S2A). DUB assays reveal that FAM63B also cleaves polyUb chains (Figure 1E). Remarkably, both FAM63A and FAM63B are highly selective at hydrolyzing K48-linked polyUb and do not cleave any of the other linkage types tested. There are several human DUBs that contain additional UBDs, but to our knowledge this is the first instance of DUBs with MIU motifs (Clague et al., 2013). Therefore, FAM63 members are unannotated human DUBs that do not bear sequence similarity to any of the known DUBs. We classify these newly discovered enzymes to be a distinct family of DUBs, which we name MINDY (MIU-containing novel DUB family), with FAM63A as MINDY-1. Orthologs of FAM63 are also present in plants, budding yeast and *Dictyostelium*. Notably, the specificity toward cleaving K48-linked polyUb is conserved in yeast YPL191C (Figure 1F), hereafter named MIY1 (MINDY deubiquitinase in yeast). In contrast, the other yeast homolog, YGL082W, does not show

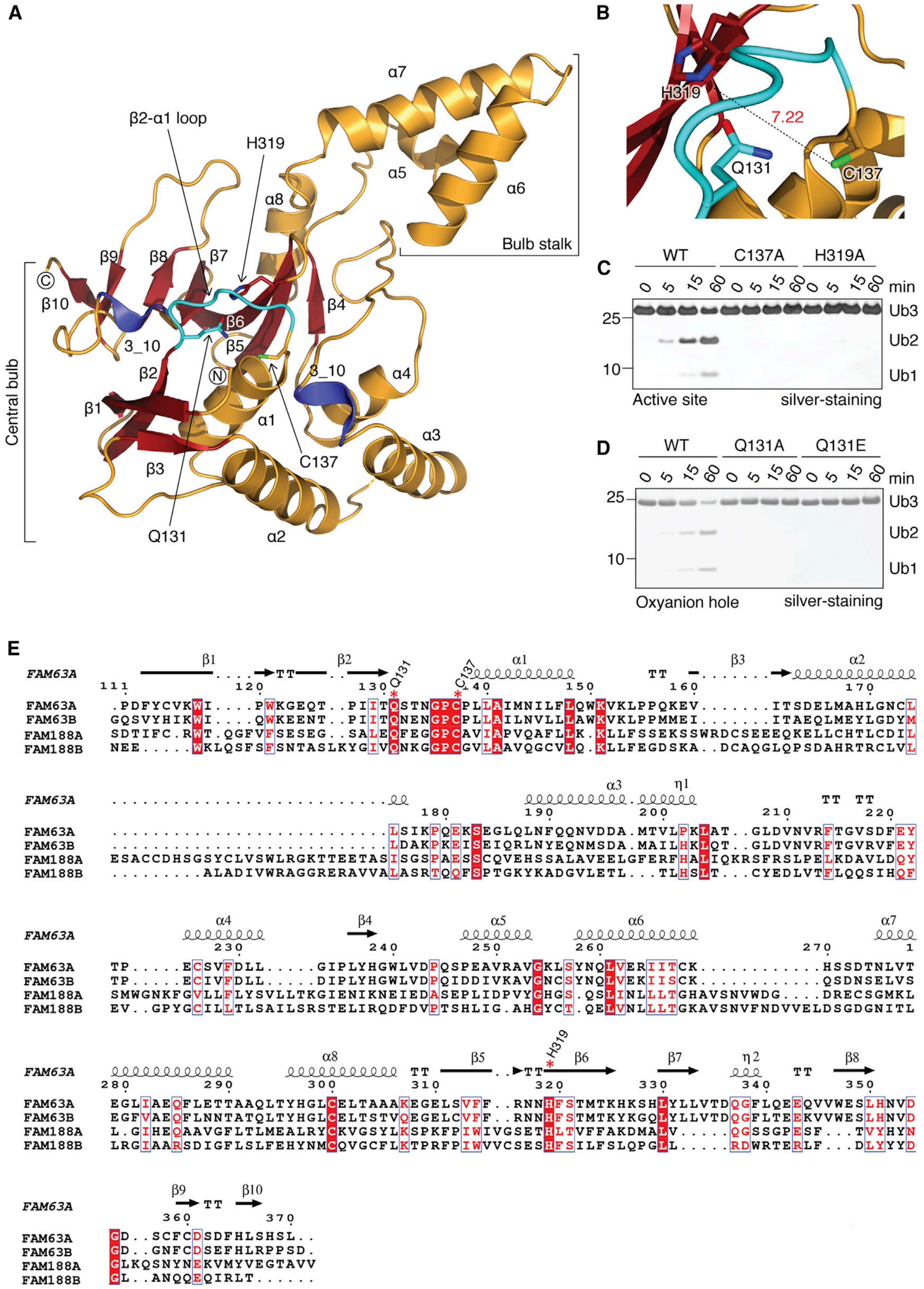

Figure 2. Crystal Structure of MINDY-1^{cat}

(A) Structure of catalytic domain of FAM63A/MINDY-1^{cat} (110–370). The Cys loop (cyan) and the catalytic residues are indicated. β sheets are colored red and 3_10 helices blue.

(B) A close-up image of the MINDY-1^{cat} catalytic site. Q131, C137, and H319 are shown.

(legend continued on next page)

any deubiquitinating activity despite the conserved catalytic residues being present (Figures 1G, S2B, and S2E).

Having identified MINDY-1/FAM63A as a new DUB, we wondered if there were other more distantly related proteins that also form part of the MINDY DUB family. Sequence analysis reveals that FAM188 members (FAM188A and FAM188B in humans) are related to FAM63 (Figure S2B). DUB assays of FAM188A against tetraUb reveal that FAM188A is also a DUB, with predicted catalytic residues C51 and H287 (Figures 1H and S2B). Interestingly, FAM188A is described as a caspase interacting pro-apoptotic protein and tumor suppressor (Liu et al., 2002; Shi et al., 2011) and has an EF hand motif inserted into the catalytic domain (Figure S2A). Despite being divergent from FAM63, FAM188A is also highly selective for the cleavage of K48-linked polyUb. Hence, we include FAM188 members in the highly conserved MINDY family of DUBs (Figure 1I).

In order to determine the mechanism of this newly identified DUB, we crystallized the minimal catalytic domain of FAM63A/MINDY-1 spanning residues 110–384 (MINDY-1^{cat}). The structure of MINDY-1^{cat} domain was determined at 3 Å resolution by X-ray crystallography (Table 1). The asymmetric unit (ASU) contains one molecule of MINDY-1^{cat} with discernible electron density for residues 110–370. The catalytic core domain of MINDY-1 with a dimension of 32 × 64 × 36 Å resembles a light bulb consisting of two subdomains, a central "bulb" subdomain that sits on a "stalk" subdomain, which resembles the base of the bulb (Figures 2A and S2C). The core of the central bulb subdomain contains a seven-stranded β sheet (β4–β10), where β7 is connected to β8 by a short 3_{10}-helix (Figures S2D and S2E). The stalk subdomain, made up of three α helices (α5–α7), forms a long helical arm that protrudes away from the central domain. A Dali search against structures in the Protein Data Bank (PDB) did not identify any matches with significant *Z* scores or sequence identity (Holm and Rosenström, 2010). Given the low homology to known protein structures, we classify MINDY-1^{cat} as a new folding variant of the diverse superfamily of cysteine proteases (Figure S3). The structure of MINDY-1^{cat} does not bear close homology to any of the known DUBs, further supporting our classification of MINDY as a new family of DUBs.

Cysteine-based DUBs usually have a catalytic triad with a catalytic Cys, a nearby His that lowers the pK_a of the catalytic Cys for nucleophilic attack, and a third residue, usually an Asp or Asn, that stabilizes the catalytic His (Reyes-Turcu et al., 2009). We predict the conserved C137 at the N terminus of helix α1 and H319 on the adjacent β6 to be the catalytic residues and these are present in a C-H architecture typical of papain-like peptidases (Figures 2B and 2E). The third catalytic residue, which serves to polarize the catalytic His, is not obvious in the observed crystal structure. Mutation of either C137 or H319 to Ala completely abolishes catalytic activity of FAM63A toward K48-linked polyUb (Figure 2C). A conserved Gln (Q131) residue N-terminal of the catalytic Cys residue may form the oxyanion hole to stabilize the negative potential formed on the carbonyl oxygen atom of the scissile bond in the transition state. Indeed, mutation of Q131 to Ala or Glu completely abolishes catalytic activity (Figure 2D). However, in the structure of isolated MINDY-1^{cat}, the active site is in an unproductive conformation and C137 is rotated out of hydrogen bond distance from the other catalytic residues (Figure 2B). Therefore, the observed structure is MINDY-1 in an inhibited state.

To gain insights into this newly discovered family of DUBs, we focused our efforts on understanding the catalytic mechanism of MINDY-1. C-terminally propargylated Ub (Ub^{Prg}) is a potent and selective covalent inhibitor of most thiol DUBs and forms a vinylthioether linkage with the catalytic cysteine (Ekkebus et al., 2013). We purified and crystallized a covalent complex between MINDY-1^{cat} and Ub^{Prg} (MINDY-1^{cat}~Ub) and determined its structure by molecular replacement (Figures S4A–S4D and Table 1). Such monoUb complexes represent a product-intermediate state with the distal Ub bound in the S1 site. There is one MINDY-1^{cat}~Ub complex in the ASU. Although the electron density for MINDY-1^{cat} is well ordered, the electron density for Ub was not easily interpretable. Refining the structure at lower resolution revealed that Ub exists in two alternate conformations (Ub^{A} and Ub^{B}), with each at ~50% occupancy (Figures 3A, S4C, and S4D). The distal Ub rests on the stalk subdomain, and the region of MINDY-1^{cat} that makes contact with Ub is conserved in evolution (Figure 3B). Mapping conserved residues on the surface of MINDY-1^{cat} reveals conserved regions around and opposite to where the distal Ub makes contacts, suggesting where the proximal Ub might bind (Figure 3B). The C terminus of Ub sits in a conserved catalytic groove, and L73 of Ub sits in a highly conserved hydrophobic pocket (Figures 3C and 3D). Mutating residues lining this hydrophobic pocket to disrupt interactions with the C terminus of Ub abolishes catalytic activity (Figure 3E). MINDY-1 therefore is similar to many other DUBs that require an interaction with L73 of the distal Ub for catalysis (Békés et al., 2013). MINDY-1 also mediates ionic interactions with the distal Ub, and mutating the key interacting residues in MINDY-1^{cat} completely abolishes catalytic activity of the DUB (Figures 3F and 3G).

Binding of Ub^{Prg} to MINDY-1^{cat} does not induce global conformational changes in the catalytic domain, and both free and MINDY-1^{cat}~Ub complex superpose well (root-mean-square deviation ~ 1 Å over 244 aligned Cα atoms). One notable difference between free and complexed states of MINDY-1^{cat} is the movement of the Cys loop (β2-α1) that in the structure of apo MINDY-1^{cat} blocks access of Ub to the catalytic site (Figures 3H, 3I, 2B, S4E, and S4F). Movement of the Cys loop also rotates the catalytic C137 to bring it closer to the catalytic H319. Intriguingly, we observe H319 to exist in two alternate conformations (Figure 3H). In one conformation, H319 is closer to the C137,

(C and D) Hydrolysis of 1.9 μM K48-linked triUb by 1.6 μM MINDY-1 wild-type (WT) and the indicated mutants of the active site residues (C) or Q131 that forms the oxyanion hole residue (D).

(E) Sequence alignment of human FAM63A, FAM63B, FAM188A, and FAM188B. Secondary structure elements are shown for MINDY-1^{cat}. The catalytic residues are highlighted with red asterisks. Residues 300–371 of FAM188A that form the EF hand domain have been omitted from the alignment. Fully conserved residues are shaded in red.

See also Figures S2 and S3.

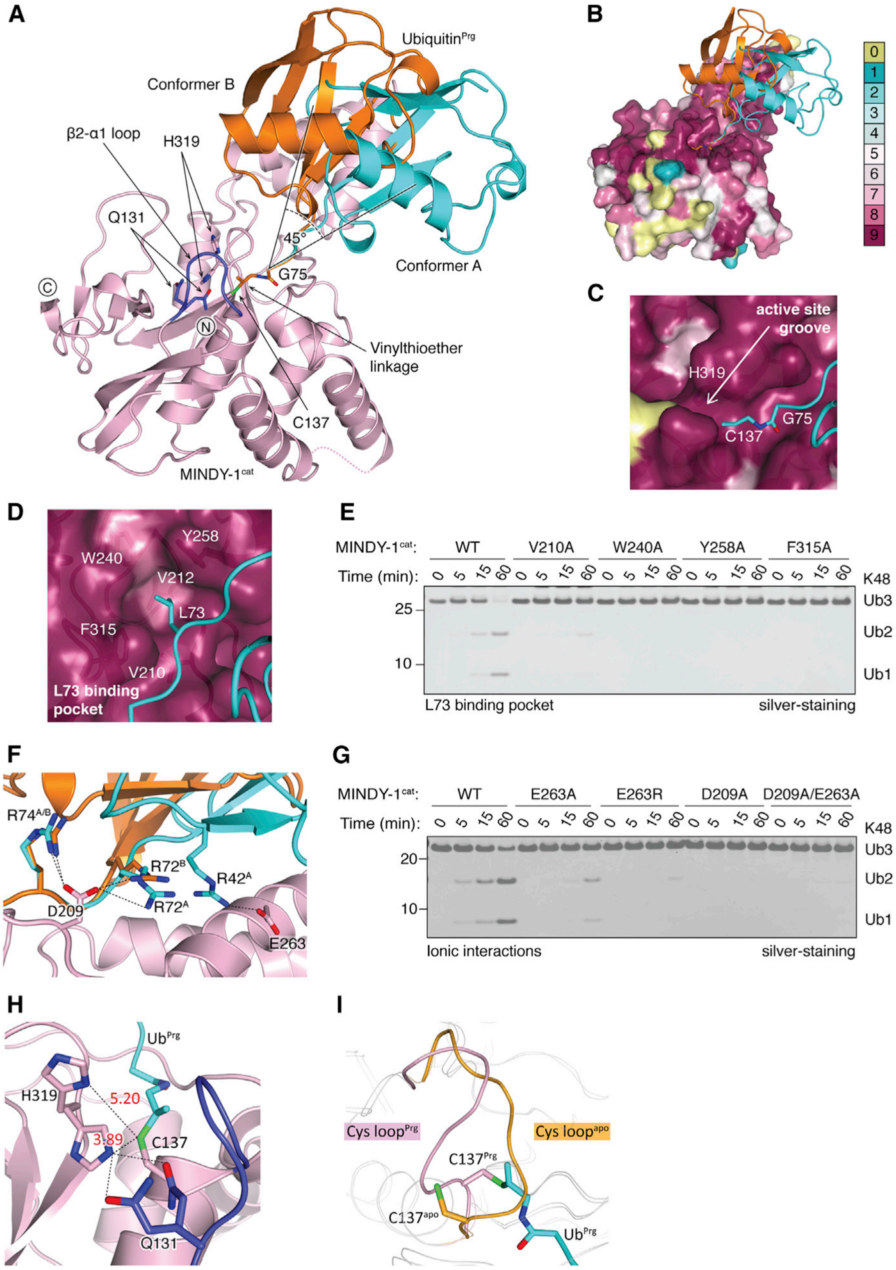

Figure 3. Structure of MINDY-1^{cat}~Ub

(A) Overall structure of the catalytic core domain of MINDY-1 (pink) covalently bound to Ub. Ub exists in two alternate conformers in the structure that are rotated by ~45° (cyan and orange). The vinylthioether linkage connecting Ub^{Prg} with MINDY-1 is shown in sticks. The Cys loop (β2-α1) is shown in blue.

(B) Conserved residues on the surface of MINDY-1 based on the sequence alignment in Figure S1C generated with the Consurf server (http://consurf.tau.ac.il) are shown. While the backside of MINDY-1^{cat} is not conserved, surfaces interacting with and around the distal Ub are conserved.

(C) Close-up view of the catalytic groove where the C terminus of Ub sits, with coloring scheme as in (B).

(D) An aromatic cage formed by V212, W240, Y258, and F315 interacts with L73 of Ub. Close-up view of the conserved hydrophobic pocket accommodating L73 colored as in (B).

(E) DUB assays monitoring cleavage of 1.9 μM K48-triUb with 1.6 μM MINDY-1^{cat} performed as in Figure 1C comparing activity of MINDY-1 and point mutants lining the L73 pocket: V210A, W240A, Y258A, and F315A.

(F) Close-up view of ionic interactions between Ub and MINDY-1.

(G) DUB assays comparing activity of MINDY-1 mutants that disrupt ionic interactions with Ub as performed in (E).

(H) Close-up image of the MINDY-1 catalytic triad showing two alternate conformations for H319 and Q131. Distances to C137 are indicated by dotted lines.

(I) Superposition of apo and complex states of MINDY-1^{cat} shows movement of the Cys loop (apo in orange and MINDY-1^{cat}~Ub complex in pink).

See also Figure S4.

resembling an active site poised for catalysis. In an alternate conformation, H319 is flipped away from C137 in a conformation in which the DUB is again in an inhibited state. Our analyses point to a substrate-induced conformational change that remodels the Cys loop and realigns the catalytic residues to an active conformation.

DUBs can either cleave within chains (endo-DUB) or remove Ubs from one end of the chain (exo-DUB), and the mode of cleavage used by a DUB provides insights into its function (Komander et al., 2009). For instance, CYLD, a negative regulator of NF-κB signaling, cleaves within chains to release Ub chains en bloc from substrates (Komander et al., 2008). In contrast, DUBs such as USP14 trim Ub chains and can edit the degradation signal on substrates to rescue them from the proteasome (Lee et al., 2010). To determine the mode of chain cleavage used by MINDY-1, we carefully monitored the time-dependent cleavage of K48-linked pentaUb chains (Figure 4A). Upon cleavage by MINDY-1^{FL} and MINDY-1^{cat}, tetraUb and monoUb are formed at the earliest time points, followed by the appearance of triUb (Figure 4A, lanes 3, 4, 10, and 11). DiUb is detected only at later time points, while there is a steady increase in the intensity of monoUb from the start (Figure 4A, lanes 3–7 and 10–14). This suggests that MINDY-1 cleaves Ub chains in a stepwise manner, releasing one Ub at a time (exo-DUB), and this mode of cleavage is not influenced by the MIU. In contrast, yeast MIY1 does not discriminate between the positions at which it cleaves within a Ub chain (endo-DUB), so all cleavage products (tetra-, tri-, di-, and monoUb) are formed at similar rates (Figure 4A, lanes 17–21). MINDY-1 has poor Ub C-terminal hydrolytic activity (Figure S5A), and two conformations are observed for the distal Ub (Figure 3A), which further suggests that the preferred substrate of MINDY-1 is Ub chains.

To explore the efficiency of MINDY-1 to cleave K48-polyUb chains, we set out to determine the kinetics of MINDY-1 and to compare its activity with OTUB1, a well-studied DUB, which is also highly selective at cleaving K48-linked chains (Wang et al., 2009). Using in-gel-based DUB assays, we monitored the cleavage of K48-linked diUb substrate that carries an infrared fluorescent dye at the distal end. We found that MINDY-1^{cat} cleaves diUb poorly, whereas MIY1 and OTUB1 both efficiently cleave diUb substrates (Figures 4B and 4C). Similarly, MINDY-1 cleaves triUb weakly, suggesting that diUb and triUb are poor substrates for MINDY-1 (Figures S5C and S5D).

Several DUBs have been reported to preferably cleave long polyUb chains (Békés et al., 2015; Todi et al., 2009). To investigate whether long polyUb chains are the preferred substrate of MINDY-1, we assembled K48-linked pentaUb chains with an infrared fluorescent label incorporated at the proximal end. When these fluorescently labeled chains were used as the substrate, MINDY-1^{cat} was as efficient as MIY1 and OTUB1 at cleaving pentaUb (Figures 4D and 4E). Interestingly, even though the rates of K48-Ub5 cleavage of MIY1 and OTUB1 are comparable with MINDY-1^{cat}, the cleavage products generated by MIY1 and OTUB1 range from monoUb to tetraUb, further supporting endo-DUB activity for MIY1 and OTUB1 (Figures 4A and 4D). On the other hand, the chain-trimming activity of MINDY-1 is clearly demonstrated by the formation of tetraUb and the subsequent formation of triUb at later time points (Figure 4D). Moreover, because the proximal Ub is fluorescently labeled, these assays also reveal a marked directionality in chain cleavage where MINDY-1 cleaves polyUb chains from the distal end. Next, we used pentaUb for kinetic analysis, because MINDY-1 cleaves pentaUb efficiently, and tetraUb is the only product formed at the early time points (Figure 4D). We determined the k_{cat} and K_m of MINDY-1 for K48-Ub5 to be $\sim$5.71 $\times$ 10^{-3} s^{-1} and $\sim$872 nM, respectively. The low K_m values suggest a strong interaction of MINDY-1 with pentaUb chains. Taken together, these results demonstrate that MINDY-1 prefers to trim longer K48-polyUb chains from the distal end.

MINDY-1 is a modular enzyme with tandem UBDs (MIU motifs) that binds selectively to K48-linked polyUb and a catalytic domain that selectively cleaves the same linkage type. In DUBs such as OTUD1 and OTUD2, the associated UBDs regulate chain linkage specificity of the DUBs (Mevissen et al., 2013). However, in MINDY-1, the UBD does not influence linkage specificity of the DUB, as the minimal catalytic domain still maintains selectivity for K48-linked polyUb (Figures 1C and 1D). To investigate the potential roles of MIU in MINDY-1, we expressed wild-type Flag-MINDY-1 or MIU mutant in HEK293 cells. Flag immunoprecipitations from cell extracts reveal high-molecular-weight K48-linked polyubiquitylated proteins associated with wild-type MINDY-1 (Figures 4G and S5I). The amount of ubiquitylated proteins associated with MINDY-1 is reduced when the MIU is mutated, suggesting a role for the MIU in targeting MINDY-1 to K48-polyubiquitylated potential substrates.

If the MIU of MINDY-1 mediates substrate targeting, then we expect it to influence the catalytic activity and cleavage of Ub chains by MINDY-1. We observed that MIU does not affect MINDY-1 activity toward K48-Ub5 chains (Figure 4A, lanes 1–14). Because MINDY-1 prefers to cleave longer polyUb chains (Figure S5H), we repeated the investigations using long K48-linked polyUb chains (pentaUb or longer) as substrates. Surprisingly, we now observe that in the absence of MIU, cleavage of long K48-chains is significantly compromised (Figure 4H). Hence in MINDY-1, the MIU regulates substrate targeting and is crucial for cleavage of long polyUb chains.

Collectively, our analysis of human MINDY-1 reveals that it is a chain-trimming enzyme, which uses the Ub-binding MIU together with the catalytic domain to cleave K48-linked polyUb chains from the distal end in a stepwise manner (Figure 4I). It is fascinating that this DUB, in addition to sensing polyUb chain linkage, also appears to sense chain length and detect the distal end of a chain. A lingering question in the field of Ub research is how long Ub chains have to be in cells in order to elicit a response. A long-held idea has been that K48 chains have to be at least four Ubs in length for efficient recognition and degradation of substrate proteins by the proteasome (Thrower et al., 2000). Recent work suggests that distributed shorter chains are more effective degradation signals (Lu et al., 2015). On the other hand, USP14 and yeast OTU1 trim K48 signals on substrates to rescue them from proteasomal degradation (Lee et al., 2010; Rumpf and Jentsch, 2006). Long K48-linked polyUb chains are poor substrates of several USP DUBs (Schaefer and Morgan, 2011). With its exquisite specificity for trimming K48 chains, MINDY-1 may alter the fate of proteins marked for degradation. Notably, while all MINDY family members analyzed are highly selective at cleaving K48-linked polyUb, we observe differences in their

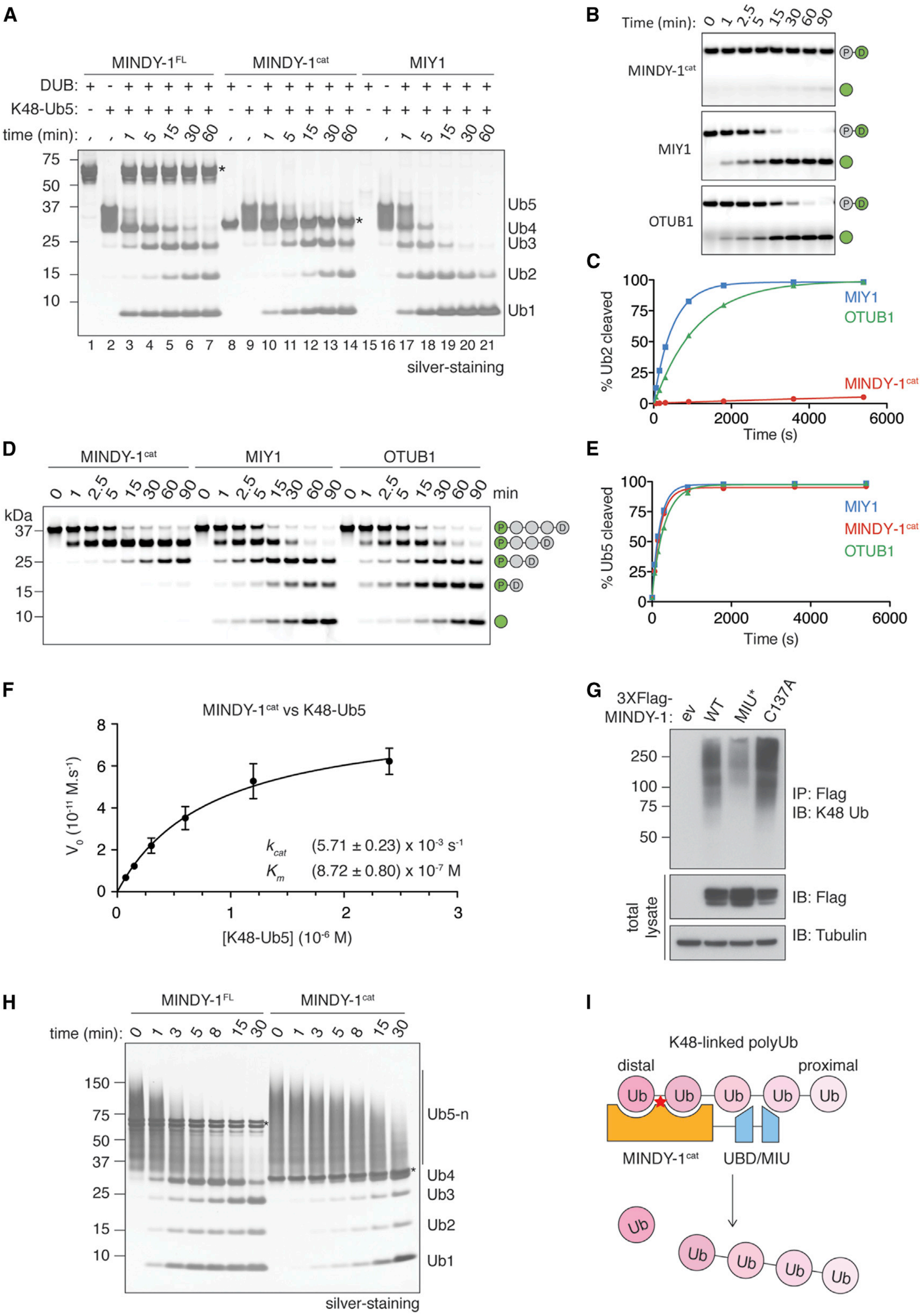

(legend on next page)

mode of action. With different domain architectures, expression, subcellular localization, and mode of action, we envisage diverse functional roles for this new family of DUBs.

EXPERIMENTAL PROCEDURES

cDNA and Antibodies

All the cDNA constructs used in this study were generated by the cloning team of the Division of Signal Transduction Therapy, MRC Protein Phosphorylation and Ubiquitylation Unit, University of Dundee, United Kingdom (Table S1). All constructs are available on request from the MRC Protein Phosphorylation and Ubiquitylation Unit reagents Web page (http://mrcppureagents.dundee.ac.uk).

Protein and epitope tags were detected by western blotting using the following antibodies: anti-K48-linked polyUb chains (05-1307; Millipore), anti-Ub antibody (Z0458; DAKO), anti-Flag M2 antibody (F1804; Sigma-Aldrich), and anti-α-Tubulin antibody (3873; Cell Signaling Technology).

Bioinformatics

Sequence database searches with generalized profiles (Bucher et al., 1996) or hidden Markov models (HMMs) (Eddy, 1998) created from multiple alignments of the FAM63 catalytic domain failed to find significant relatives outside the FAM63 family, with members of the FAM188 family yielding slightly sub-significant scores. However, the application of the HHSEARCH software (Söding, 2005), which performs HMM-to-HMM comparisons, allowed to establish a significant relationship between the FAM63 and FAM188 families. An HMM made from a FAM63 alignment matched the FAM188 family with a p value of 9.2×10^{-7}. Conversely, an HMM made from the FAM188 family matched the FAM63 family with a p value of 7.9×10^{-7}. The resulting HMM-to-HMM alignment revealed a perfect conservation of the active site residue, suggesting a functional similarity between the two distantly related protein families. Multiple sequence alignments were created in Jalview (Waterhouse et al., 2009) by using the L-INS-I algorithm of the MAFFT package (Katoh and Toh, 2010). Phylogenetic trees were generated using PhyML (Dereeper et al., 2008) (http://phylogeny.lirmm.fr) using bootstrapping with alignment of catalytic domains as input and trees rendered using TreeDyn (http://phylogeny.lirmm.fr).

UBD Linkage Specificity Analysis

TetraUb chains of the different linkage types were assembled and purified as described previously (Kristariyanto et al., 2015a, 2015b). Briefly, 58.5 nM tetraUb chains in 500 μl pull-down buffer (50 mM Tris-HCl [pH 7.5], 150 mM NaCl, 0.1% NP-40, 1 mM DTT, 0.5 mg/ml BSA) was captured using 10.5 nmol Halo-MINDY-1 (388–426) immobilized on HaloLink resin for 2 hr at 4°C. Captured materials were analyzed on silver-stained SDS-PAGE gel. For details, see Supplemental Experimental Procedures.

Crystallization and Structure Determination

MINDY-1^{cat} and MINDY-1^{cat}-Ub^{Prg} complex were purified and crystallized and their structures determined as detailed in Supplemental Experimental Procedures.

ACCESSION NUMBERS

The accession numbers for the structures reported in this paper are PDB: 5JKN and 5JQS.

SUPPLEMENTAL INFORMATION

Supplemental Information includes Supplemental Experimental Procedures, five figures, and one table and can be found with this article online at http://dx.doi.org/10.1016/j.molcel.2016.05.009.

AUTHOR CONTRIBUTIONS

S.A.A.R., Y.A.K., and S.-Y.C. designed and performed all experiments. S.A.A.R. performed structure determination and analysis. P.J.N. and K.L. contributed with reagents and analysis. K.H. performed sequence analysis. S.W. cloned all the DNA constructs. Y.K. supervised the study and wrote the manuscript with input from all the authors.

ACKNOWLEDGMENTS

We thank Drs. H. Walden, R.T. Hay, S. Virdee, R. Enchev, and M. Swamy, members of the Kulathu, Walden, and Hofmann labs, for discussions and critical comments on the manuscript. We thank Dr. A. Knebel for reagents and Dr. A Murzin (MRC Laboratory of Molecular Biology) for his input on the structural classification. We are also indebted to reviewer 2 for his or her advice. Crystallographic data were collected at the European Synchrotron Radiation Facility (ID29 and ID23-1) and at Diamond Light Source (I02). This work was supported by the Medical Research Council UK (MC_UU_12016/6, Ub signaling mechanisms), Tenovus Scotland, the EMBO Young Investigator Programme, and the pharmaceutical companies supporting the Division of Signal Transduction Therapy (AstraZeneca, Boehringer-Ingelheim, GlaxoSmithKline, Merck KGaA, Janssen Pharmaceutica, and Pfizer).

Received: February 9, 2016
Revised: April 15, 2016
Accepted: May 5, 2016
Published: June 9, 2016

Figure 4. MINDY-1 Cleaves PolyUb Chains in a Stepwise Manner

(A) Time course of cleavage of 3.5 μM K48-pentaUb by 1.6 μM of full-length MINDY-1 and MINDY-1^{cat} and 160 nM MIY1. Asterisks indicate MINDY-1.

(B) Kinetics of cleavage of fluorescently labeled K48-linked diUb by MINDY-1^{cat}, MIY1, and OTUB1. DUBs (1 μM) were incubated with 500 nM of K48-linked diUb that has been labeled with an infrared fluorescent dye at its distal Ub (green circle) for the indicated times. Fluorescent Ub was visualized using Odyssey LI-COR system at 800 nm channel. D, distal Ub, P, proximal Ub.

(C) Quantification of K48-Ub2 hydrolysis by MINDY-1^{cat}, MIY1, and OTUB1 in (B). Percentage of the formed Ub1 intensity is shown on the y axis (n = 3; mean ± SD).

(D) DUB assays monitoring time-dependent cleavage of fluorescently labeled pentaUb by MINDY-1^{cat}, MIY1, and OTUB1 as in (B). The proximal Ub of the chain (indicated by green circle) was labeled with an infrared fluorescent dye.

(E) Quantification of cleavage of K48-linked pentaUb by MINDY-1^{cat}, MIY1, and OTUB1 in (D). The percentage of the total intensities of Ub4, Ub3, Ub2, and Ub1 formed is shown on the y axis (n = 3; mean ± SD). See also Figure S5.

(F) Steady-state kinetics of K48-linked pentaUb cleavage by MINDY-1^{cat}. MINDY-1^{cat} (15 nM) was incubated with 0.075–2.4 μM fluorescently labeled pentaUb (IR-K48-Ub5). The K48-Ub4 formed at the early time point (less than 10% of the substrate) was quantified to obtain initial velocities (V_0). V_0 was plotted against IR-K48-Ub5 concentration, and the data were fitted to the Michaelis-Menten equation to derive k_{cat} and K_m (n = 3; mean ± SD).

(G) Flag pull-downs from extracts of HEK293 cells inducibly expressing the indicated full-length MINDY-1 constructs. ev, empty vector; MIU*, MIU mutant, L415A/A416G. Immunoblotting with a K48-linkage specific antibody was performed to monitor captured polyUb material.

(H) Time course comparing hydrolysis of K48-polyUb chains containing at least 5 Ub by full-length MINDY-1 and MINDY-1^{cat}, which lacks the MIU.

(I) Model depicting the synergy between different domains of MINDY-1, where the UBD mediates substrate targeting to result in trimming of the Ub chain from the distal end by the catalytic domain.

See also Figure S5.

REFERENCES

Békés, M., Okamoto, K., Crist, S.B., Jones, M.J., Chapman, J.R., Brasher, B.B., Melandri, F.D., Ueberheide, B.M., Denchi, E.L., and Huang, T.T. (2013). DUB-resistant ubiquitin to survey ubiquitination switches in mammalian cells. Cell Rep. *5*, 826–838.

Békés, M., Rut, W., Kasperkiewicz, P., Mulder, M.P.C., Ovaa, H., Drag, M., Lima, C.D., and Huang, T.T. (2015). SARS hCoV papain-like protease is a unique Lys48 linkage-specific di-distributive deubiquitinating enzyme. Biochem. J. *468*, 215–226.

Bucher, P., Karplus, K., Moeri, N., and Hofmann, K. (1996). A flexible motif search technique based on generalized profiles. Comput. Chem. *20*, 3–23.

Clague, M.J., Coulson, J.M., and Urbé, S. (2012). Cellular functions of the DUBs. J. Cell Sci. *125*, 277–286.

Clague, M.J., Barsukov, I., Coulson, J.M., Liu, H., Rigden, D.J., and Urbé, S. (2013). Deubiquitylases from genes to organism. Physiol. Rev. *93*, 1289–1315.

Dereeper, A., Guignon, V., Blanc, G., Audic, S., Buffet, S., Chevenet, F., Dufayard, J.F., Guindon, S., Lefort, V., Lescot, M., et al. (2008). Phylogeny.fr: robust phylogenetic analysis for the non-specialist. Nucleic Acids Res. *36*, W465–W469.

Di Fiore, P.P., Polo, S., and Hofmann, K. (2003). When ubiquitin meets ubiquitin receptors: a signalling connection. Nat. Rev. Mol. Cell Biol. *4*, 491–497.

Eddy, S.R. (1998). Profile hidden Markov models. Bioinformatics *14*, 755–763.

Ekkebus, R., van Kasteren, S.I., Kulathu, Y., Scholten, A., Berlin, I., Geurink, P.P., de Jong, A., Goerdayal, S., Neefjes, J., Heck, A.J.R., et al. (2013). On terminal alkynes that can react with active-site cysteine nucleophiles in proteases. J. Am. Chem. Soc. *135*, 2867–2870.

Eletr, Z.M., and Wilkinson, K.D. (2014). Regulation of proteolysis by human deubiquitinating enzymes. Biochim. Biophys. Acta *1843*, 114–128.

Finley, D. (2009). Recognition and processing of ubiquitin-protein conjugates by the proteasome. Annu. Rev. Biochem. *78*, 477–513.

Heideker, J., and Wertz, I.E. (2015). DUBs, the regulation of cell identity and disease. Biochem. J. *465*, 1–26.

Holm, L., and Rosenström, P. (2010). Dali server: conservation mapping in 3D. Nucleic Acids Res. *38*, W545–W549.

Inobe, T., and Matouschek, A. (2014). Paradigms of protein degradation by the proteasome. Curr. Opin. Struct. Biol. *24*, 156–164.

Katoh, K., and Toh, H. (2010). Parallelization of the MAFFT multiple sequence alignment program. Bioinformatics *26*, 1899–1900.

Kelley, L.A., Mezulis, S., Yates, C.M., Wass, M.N., and Sternberg, M.J.E. (2015). The Phyre2 web portal for protein modeling, prediction and analysis. Nat. Protoc. *10*, 845–858.

King, R.W., and Finley, D. (2014). Sculpting the proteome with small molecules. Nat. Chem. Biol. *10*, 870–874.

Komander, D., Lord, C.J., Scheel, H., Swift, S., Hofmann, K., Ashworth, A., and Barford, D. (2008). The structure of the CYLD USP domain explains its specificity for Lys63-linked polyubiquitin and reveals a B box module. Mol. Cell *29*, 451–464.

Komander, D., Clague, M.J., and Urbé, S. (2009). Breaking the chains: structure and function of the deubiquitinases. Nat. Rev. Mol. Cell Biol. *10*, 550–563.

Kristariyanto, Y.A., Choi, S.-Y., Rehman, S.A.A., Ritorto, M.S., Campbell, D.G., Morrice, N.A., Toth, R., and Kulathu, Y. (2015a). Assembly and structure of Lys33-linked polyubiquitin reveals distinct conformations. Biochem. J. *467*, 345–352.

Kristariyanto, Y.A., Abdul Rehman, S.A., Campbell, D.G., Morrice, N.A., Johnson, C., Toth, R., and Kulathu, Y. (2015b). K29-selective ubiquitin binding domain reveals structural basis of specificity and heterotypic nature of k29 polyubiquitin. Mol. Cell *58*, 83–94.

Kulathu, Y., and Komander, D. (2012). Atypical ubiquitylation - the unexplored world of polyubiquitin beyond Lys48 and Lys63 linkages. Nat. Rev. Mol. Cell Biol. *13*, 508–523.

Lam, Y.A., Xu, W., DeMartino, G.N., and Cohen, R.E. (1997). Editing of ubiquitin conjugates by an isopeptidase in the 26S proteasome. Nature *385*, 737–740.

Lee, S., Tsai, Y.C., Mattera, R., Smith, W.J., Kostelansky, M.S., Weissman, A.M., Bonifacino, J.S., and Hurley, J.H. (2006). Structural basis for ubiquitin recognition and autoubiquitination by Rabex-5. Nat. Struct. Mol. Biol. *13*, 264–271.

Lee, B.-H., Lee, M.J., Park, S., Oh, D.-C., Elsasser, S., Chen, P.-C., Gartner, C., Dimova, N., Hanna, J., Gygi, S.P., et al. (2010). Enhancement of proteasome activity by a small-molecule inhibitor of USP14. Nature *467*, 179–184.

Liu, B., Liu, Y., Chen, J., Wei, Z., Yu, H., Zhen, Y., Lu, L., and Hui, R. (2002). CARP is a novel caspase recruitment domain containing pro-apoptotic protein. Biochem. Biophys. Res. Commun. *293*, 1396–1404.

Lu, Y., Lee, B.-H., King, R.W., Finley, D., and Kirschner, M.W. (2015). Substrate degradation by the proteasome: a single-molecule kinetic analysis. Science *348*, 1250834.

MacGurn, J.A., Hsu, P.-C., and Emr, S.D. (2012). Ubiquitin and membrane protein turnover: from cradle to grave. Annu. Rev. Biochem. *81*, 231–259.

Marcotte, E.M., Pellegrini, M., Ng, H.L., Rice, D.W., Yeates, T.O., and Eisenberg, D. (1999). Detecting protein function and protein-protein interactions from genome sequences. Science *285*, 751–753.

Mevissen, T.E.T., Hospenthal, M.K., Geurink, P.P., Elliott, P.R., Akutsu, M., Arnaudo, N., Ekkebus, R., Kulathu, Y., Wauer, T., El Oualid, F., et al. (2013). OTU deubiquitinases reveal mechanisms of linkage specificity and enable ubiquitin chain restriction analysis. Cell *154*, 169–184.

Nijman, S.M.B., Luna-Vargas, M.P.A., Velds, A., Brummelkamp, T.R., Dirac, A.M.G., Sixma, T.K., and Bernards, R. (2005). A genomic and functional inventory of deubiquitinating enzymes. Cell *123*, 773–786.

Penengo, L., Mapelli, M., Murachelli, A.G., Confalonieri, S., Magri, L., Musacchio, A., Di Fiore, P.P., Polo, S., and Schneider, T.R. (2006). Crystal structure of the ubiquitin binding domains of rabex-5 reveals two modes of interaction with ubiquitin. Cell *124*, 1183–1195.

Reyes-Turcu, F.E., Ventii, K.H., and Wilkinson, K.D. (2009). Regulation and cellular roles of ubiquitin-specific deubiquitinating enzymes. Annu. Rev. Biochem. *78*, 363–397.

Rumpf, S., and Jentsch, S. (2006). Functional division of substrate processing cofactors of the ubiquitin-selective Cdc48 chaperone. Mol. Cell *21*, 261–269.

Schaefer, J.B., and Morgan, D.O. (2011). Protein-linked ubiquitin chain structure restricts activity of deubiquitinating enzymes. J. Biol. Chem. *286*, 45186–45196.

Shi, Y., Chen, J., Li, Z., Zhang, Z., Yu, H., Sun, K., Wang, X., Song, X., Wang, Y., Zhen, Y., et al. (2011). C10ORF97 is a novel tumor-suppressor gene of non-small-cell lung cancer and a functional variant of this gene increases the risk of non-small-cell lung cancer. Oncogene *30*, 4107–4117.

Söding, J. (2005). Protein homology detection by HMM-HMM comparison. Bioinformatics *21*, 951–960.

Thrower, J.S., Hoffman, L., Rechsteiner, M., and Pickart, C.M. (2000). Recognition of the polyubiquitin proteolytic signal. EMBO J. *19*, 94–102.

Todi, S.V., Winborn, B.J., Scaglione, K.M., Blount, J.R., Travis, S.M., and Paulson, H.L. (2009). Ubiquitination directly enhances activity of the deubiquitinating enzyme ataxin-3. EMBO J. *28*, 372–382.

Wang, T., Yin, L., Cooper, E.M., Lai, M.-Y., Dickey, S., Pickart, C.M., Fushman, D., Wilkinson, K.D., Cohen, R.E., and Wolberger, C. (2009). Evidence for bidentate substrate binding as the basis for the K48 linkage specificity of otubain 1. J. Mol. Biol. *386*, 1011–1023.

Waterhouse, A.M., Procter, J.B., Martin, D.M.A., Clamp, M., and Barton, G.J. (2009). Jalview Version 2–a multiple sequence alignment editor and analysis workbench. Bioinformatics *25*, 1189–1191.

Molecular Cell, Volume *63*

Supplemental Information

MINDY-1 Is a Member of an Evolutionarily Conserved and Structurally Distinct New Family of Deubiquitinating Enzymes

Syed Arif Abdul Rehman, Yosua Adi Kristariyanto, Soo-Youn Choi, Pedro Junior Nkosi, Simone Weidlich, Karim Labib, Kay Hofmann, and Yogesh Kulathu

Supplemental Materials:

Supplemental Figures

Figure S1

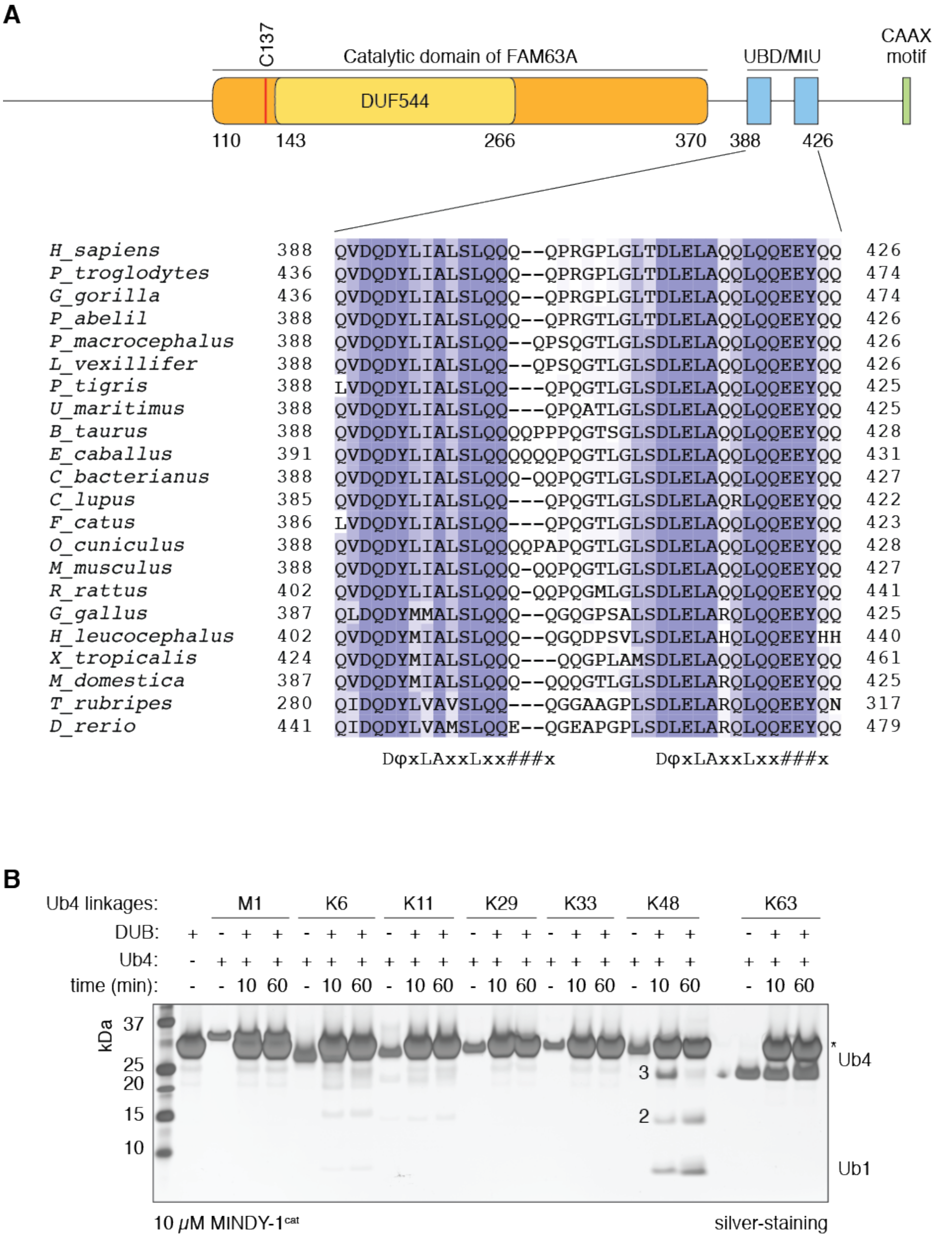

Figure S1

C

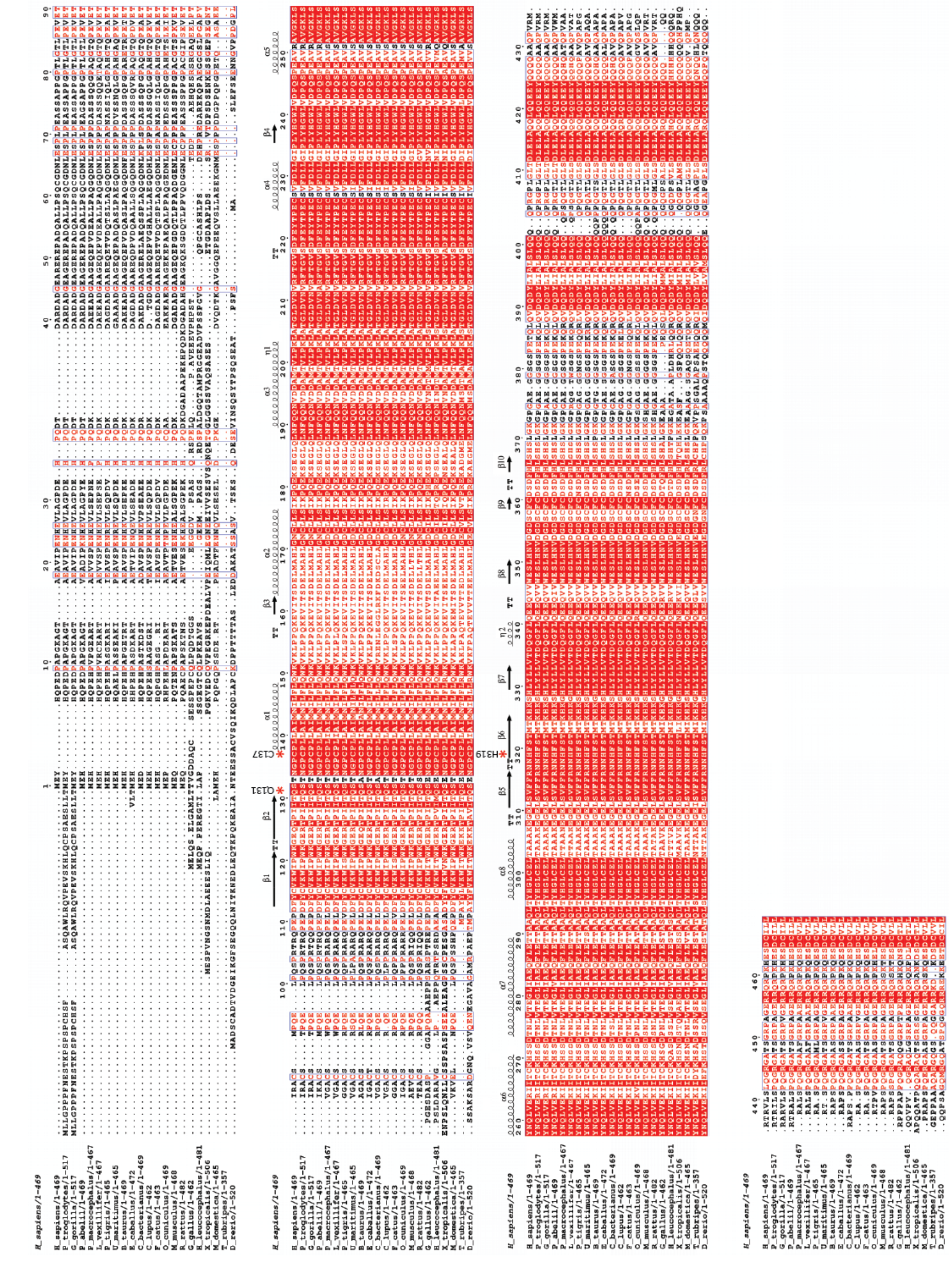

Figure S1

D

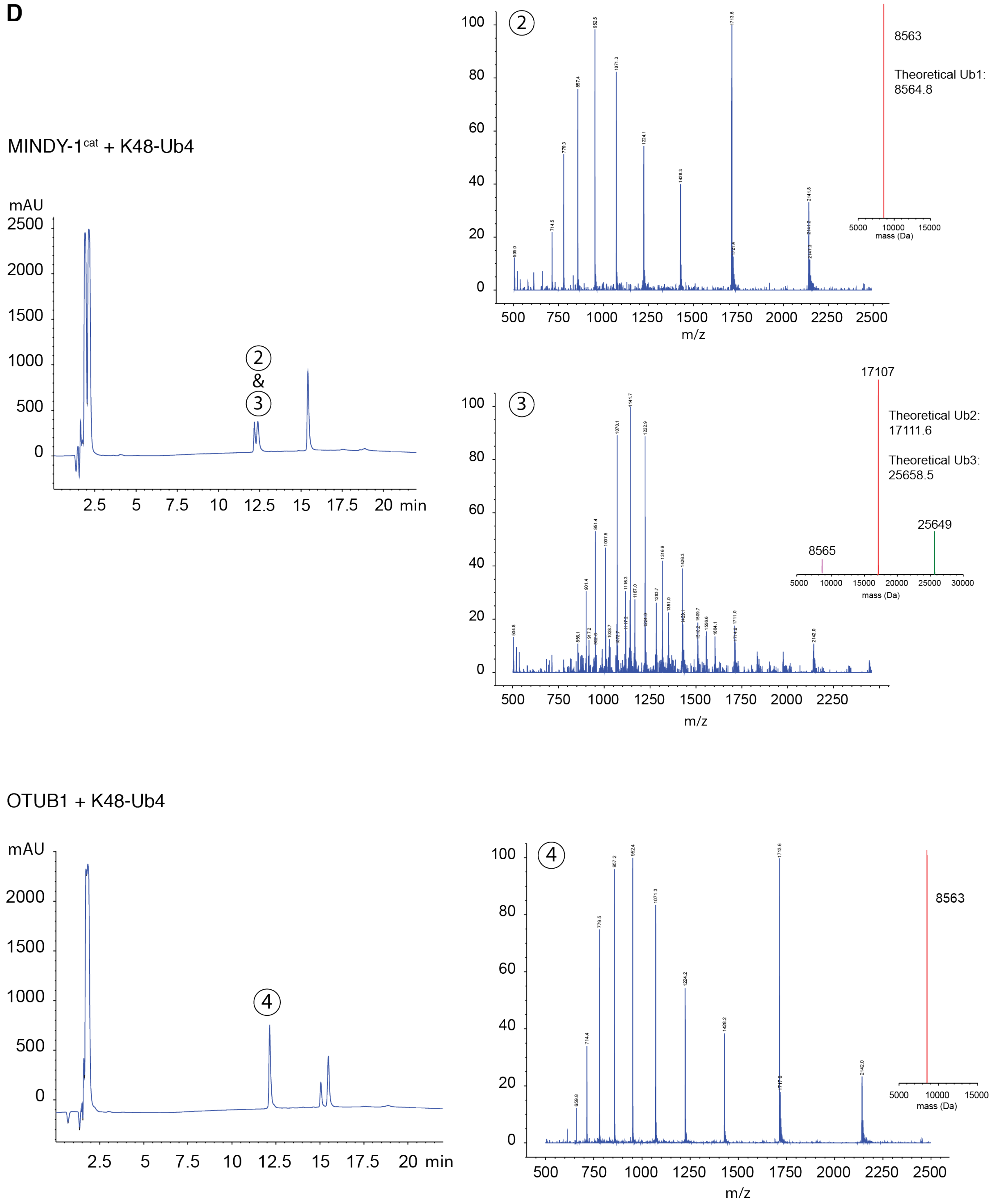

MINDY-1^{cat} + K48-Ub4
mAU
2500
2000
1500
1000
500
0
2.5 5 7.5 10 12.5 15 17.5 20 min
2 & 3
2
100 80 60 40 20 0
500 750 1000 1250 1500 1750 2000 2250 2500
m/z
8563
Theoretical Ub1:
8564.8
5000 10000 15000
mass (Da)
3
17107
Theoretical Ub2:
17111.6
Theoretical Ub3:
25658.5
25649
8565
5000 10000 15000 20000 25000 30000
mass (Da)
500 750 1000 1250 1500 1750 2000 2250
m/z
OTUB1 + K48-Ub4
mAU
2000
1500
1000
500
0
2.5 5 7.5 10 12.5 15 17.5 20 min
4
4
100 80 60 40 20 0
500 750 1000 1250 1500 1750 2000 2250 2500
m/z
8563
5000 10000 15000
mass (Da)

Figure S1. FAM63A/MINDY-1 is a novel DUB containing tandem MIU motifs. Related to Figure 1.
(A) FAM63A contains motifs interacting with ubiquitin (MIU) at the C-terminus. Schematic representation of domain structure of human FAM63A is shown. Sequences of FAM63A from different organisms corresponding to human FAM63A 388-426 were aligned and the conserved residues are highlighted in purple. Consensus motif of MIU (Penengo et al., 2006) is conserved in two clusters within this 388-426 stretch, suggesting a possibility of tandem MIU.
(B) The catalytic domain of MINDY-1 maintains K48-linkage specificity even at high enzyme concentrations. 10 μM MINDY-1^{cat} was incubated with 2.2 μM of tetraUb of different linkage types for the indicated period of time.
(C) The catalytic domain of MINDY-1 is conserved in evolution. Sequence alignment of MINDY-1/FAM63A from different organisms is shown. Secondary structure elements are shown for MINDY-1^{cat}. The catalytic residues are highlighted with asterisks. Fully conserved residues are shaded in red.
(D) MINDY-1 cleaves K48-polyUb chains at G76. DUB assay reactions of 2.2 μM K48-Ub4 and 1.6 μM MINDY-1^{cat} or OTUB1 were carried out for 1 h at 30 °C. Left panel, samples at the end point reaction were injected and monitored by HPLC at 214 nm. Right panel, ESI-MS mass spectrum for products of DUB reaction. Inset shows the deconvoluted mass spectrum. The theoretical masses of Ub1, Ub2, and Ub3 are indicated. After 60 min of reaction, MINDY-1^{cat} produced Ub1, Ub2, and Ub3 with observed mass close to the theoretical value, indicating that the MINDY-1^{cat} cleaves K48-polyUb chains at G76. Cleavage by OTUB1 was used as a control (lower panel).

Figure S2

A

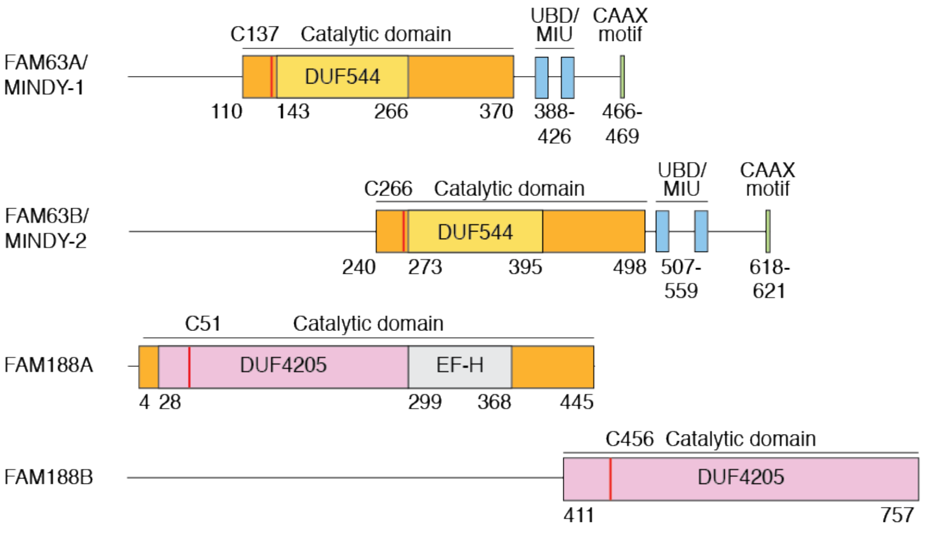
FAM63A/
MINDY-1
C137 Catalytic domain
UBD/
MIU
CAAX
motif
DUF544
110
143
266
370
388-
426
466-
469
FAM63B/
MINDY-2
C266 Catalytic domain
UBD/
MIU
CAAX
motif
DUF544
240
273
395
498
507-
559
618-
621
FAM188A
C51 Catalytic domain
DUF4205
EF-H
4
28
299
368
445
FAM188B
C456 Catalytic domain
DUF4205
411
757

B

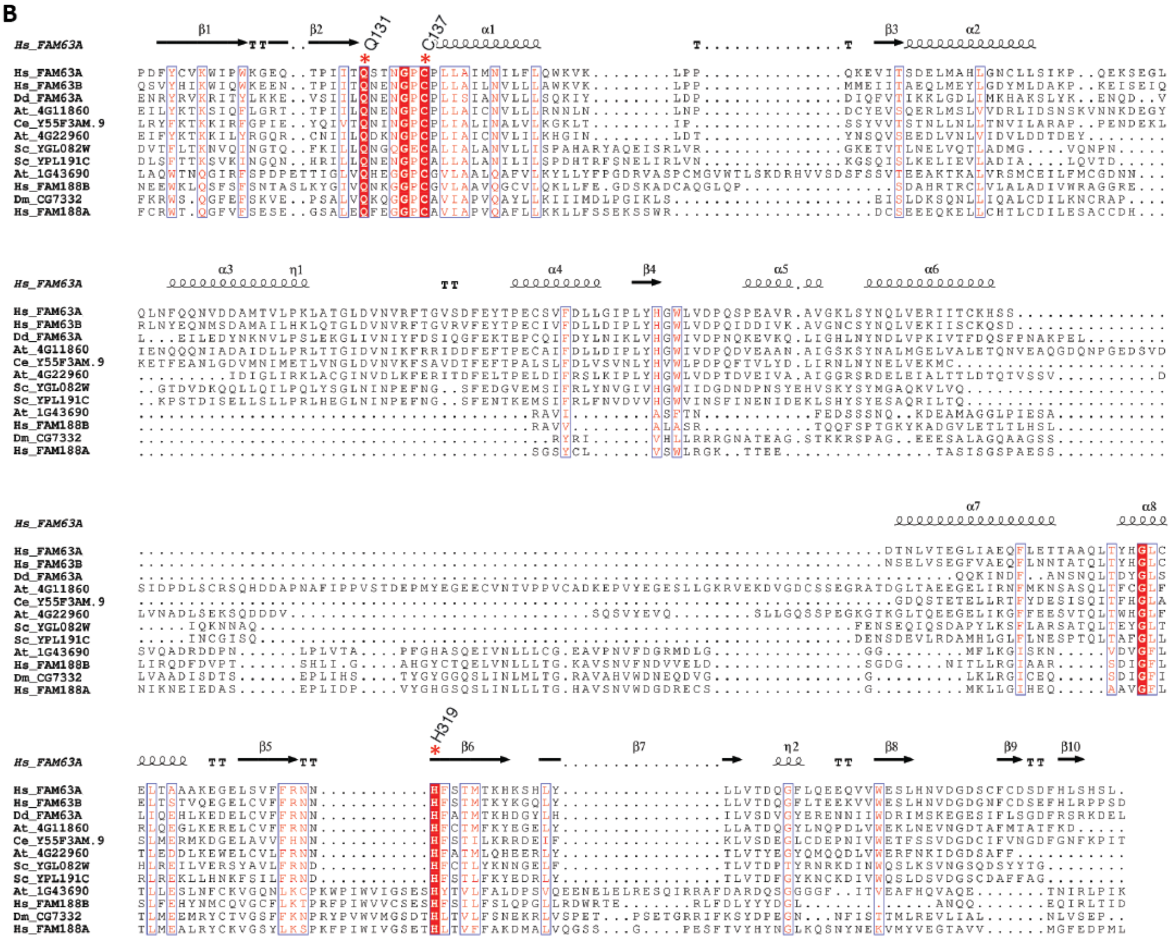
Hs_FAM63A
Q131
C137
H319
Hs_FAM63A
Hs_FAM63B
Dd_FAM63A
At_4G11860
Ce_Y55F3AM.9
At_4G22960
Sc_YGL082W
Sc_YPL191C
At_1G43690
Hs_FAM188B
Dm_CG7332
Hs_FAM188A

Figure S2

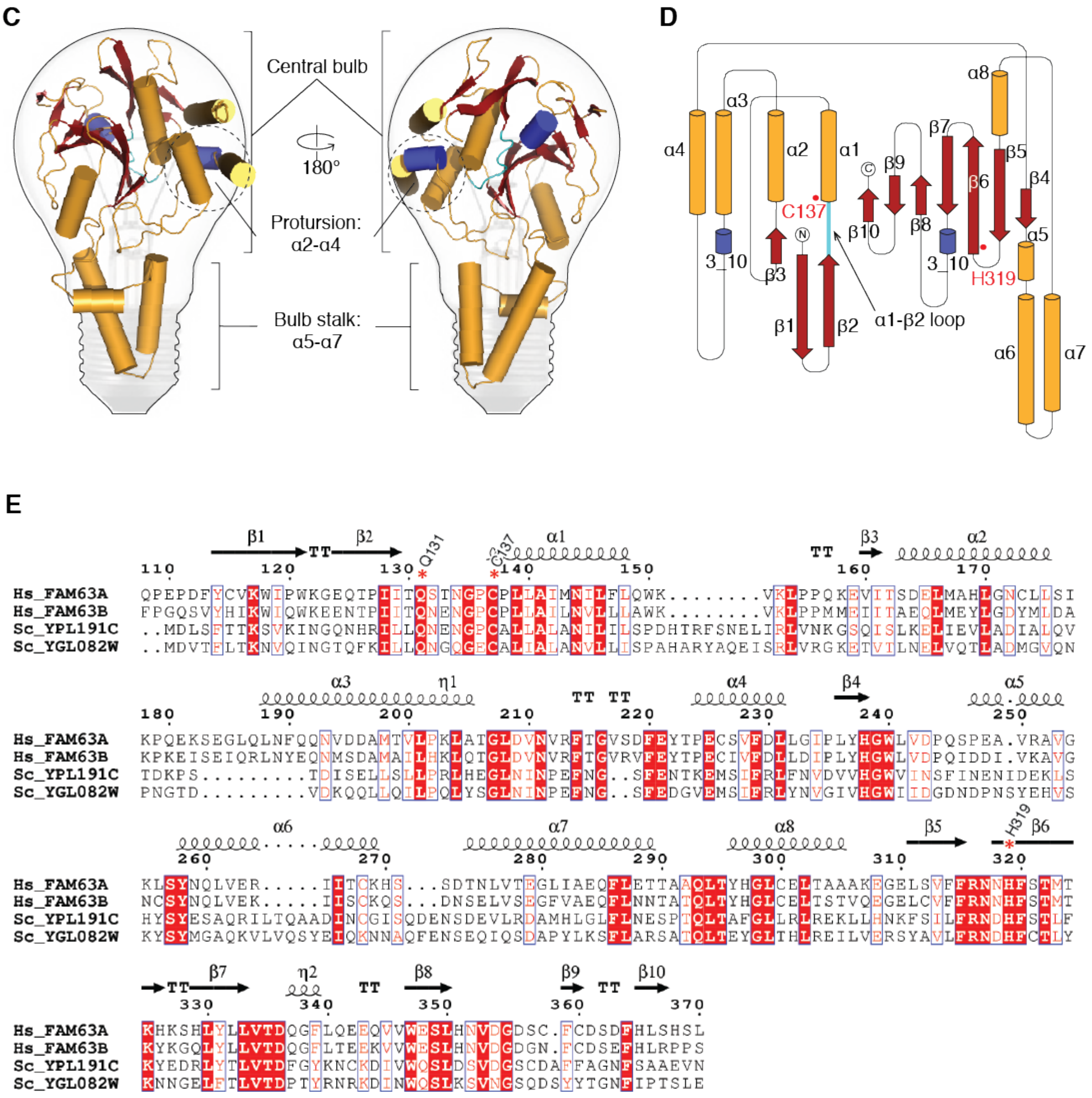

Figure S2. Sequence analysis of MINDY family of DUBs. Related to Figure 1.

(A) Schematic representation of domain structure of the human proteins of FAM63A/MINDY-1, FAM63B/MINDY-2, FAM188A, and FAM188B.

(B) Sequence alignment of the catalytic domain residues of MINDY family DUBs. Secondary structure elements are shown for MINDY-1^{cat}. The catalytic residues are highlighted with asterisks. Fully conserved residues are shaded in red. For the generation of this alignment, residues spanning the EF Hand motif (300 - 371) in FAM188A were left out. (Hs: *H. sapiens*, Dd: *D. discoideum*, At: *A.* thaliana, Ce: *C. elegans*; Sc: *S. cerevisiae*, Dm: *D. melanogaster*).

(C) The crystal structure of MINDY-1^{cat} resembles a light bulb. The structure is displayed in two different orientations, rotated by 180° around the y-axis.

(D) Topology of MINDY-1^{cat} structure. α-helices (orange), β-strands (red), and 3_10 helices (blue), catalytic residues, and the Cys loop connecting β2 and α1 (cyan) are indicated.

(E) Sequence alignment of human and yeast FAM63 members as in Figure S1C.

Figure S3

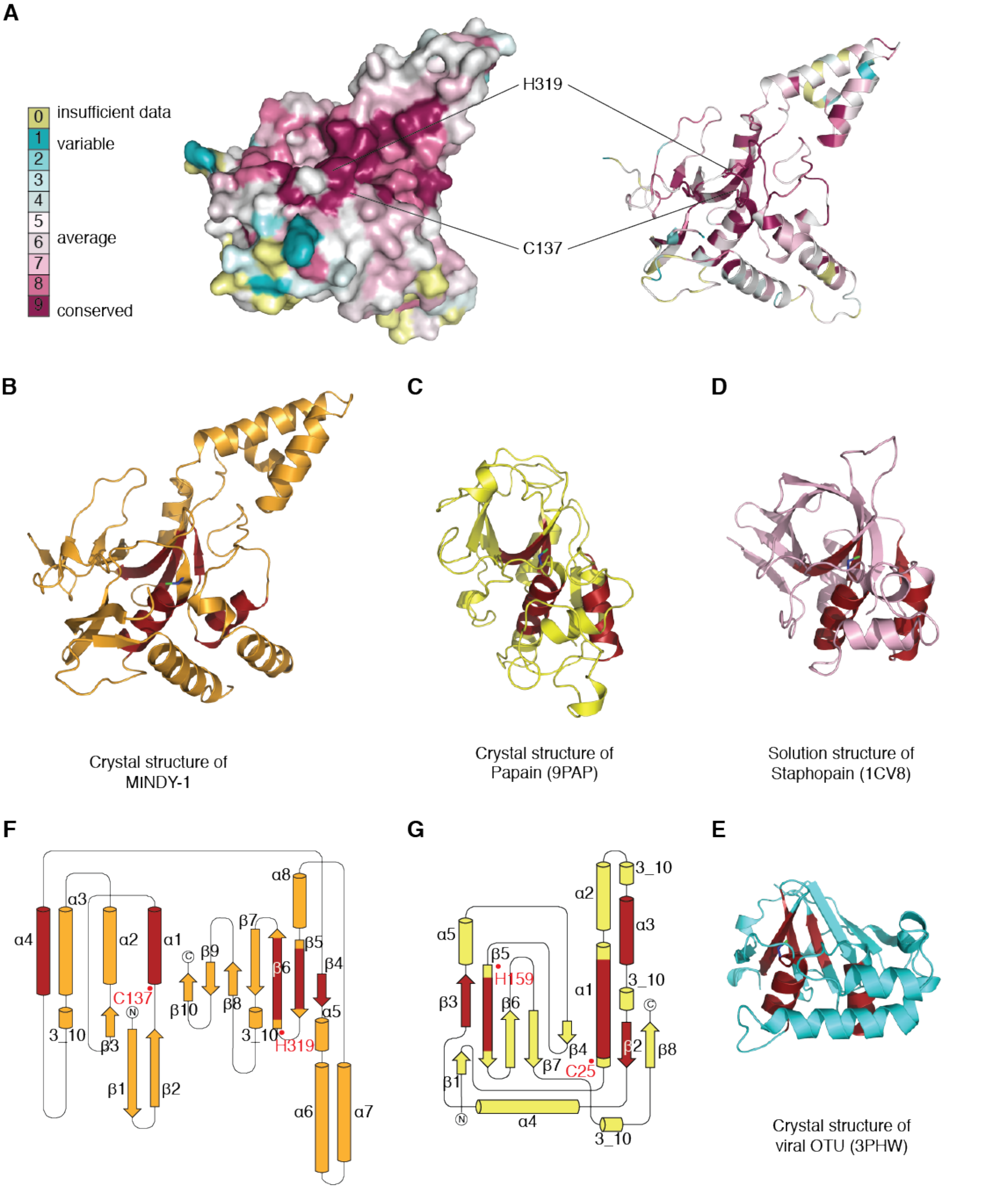

Figure S3. The crystal structure of MINDY-1^{cat} reveals a new folding variant. Related to Figure 2.
(A) Conserved residues on the surface of MINDY-1^{cat} based on sequence alignment of FAM63A and FAM63B members from different species generated with the Consurf server (http://consurf.tau.ac.il) are shown in surface and cartoon representation.
(B-E) Crystal structure of MINDY-1 (orange) (B), crystal structure of Papain (yellow) (C), solution structure of Staphopain (pink) (D), and crystal structure of viral OTU (cyan) (E) are shown in a cartoon representation. The conserved regions among these structures are highlighted in red. Multiple comparison and 3D alignment of the protein structures was performed using PDBeFold (http://www.ebi.ac.uk/msd-srv/ssm/). PDB ID: 9PAP, 1CV8, and 3PHW (Akutsu et al., 2011; Kamphuis et al., 1984). The crystal structures of Staphopain from *S. aureus* and Papain share structural identities of 14% and 9%, respectively. The structures superpose to MINDY-1^{cat} structure with rmsd values of 3.5 Å and 4.1 Å over 110 and 116 aligned Cα, respectively.
(F-G) Topology of MINDY-1^{cat} (F) and Papapin (G) structures with regions coloured as in (B).

Figure S4

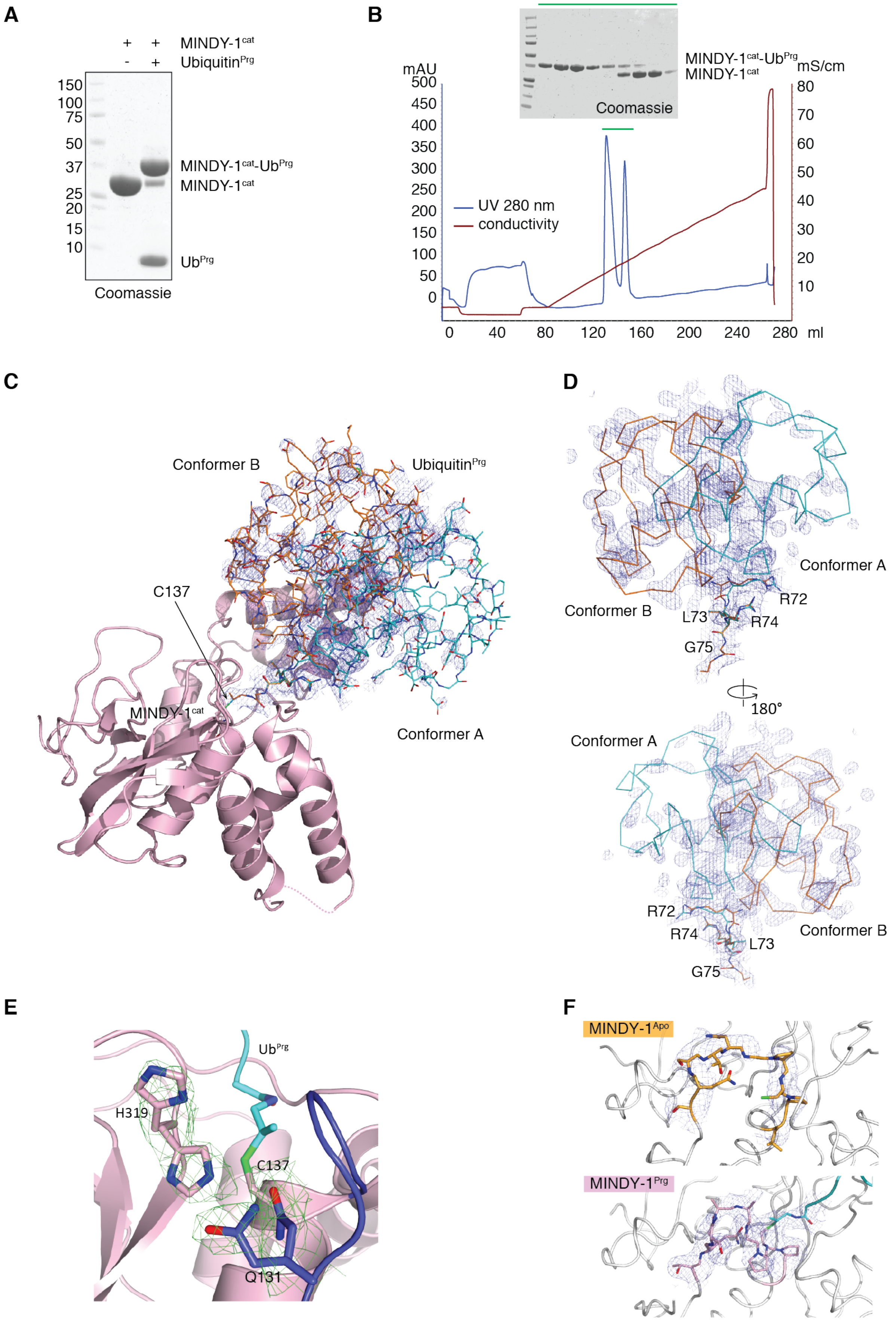
A
+ + MINDY-1cat
- + UbiquitinPrg
150
100
75
50
37
25
20
15
10
MINDY-1cat-UbPrg
MINDY-1cat
UbPrg
Coomassie
B
MINDY-1cat-UbPrg
MINDY-1cat
Coomassie
mAU
mS/cm
UV 280 nm
conductivity
ml
C
Conformer B
UbiquitinPrg
C137
MINDY-1cat
Conformer A
D
Conformer A
Conformer B
R72
L73
R74
G75
180°
E
UbPrg
H319
C137
Q131
F
MINDY-1Apo
MINDY-1Prg

Figure S4. Crystal structure of MINDY-1^{cat}~Ub^{Prg}. Related to Figure 3.
(A) Coomassie stained gel showing the reaction of MINDY-1^{cat} with propargylated Ub.
(B) Purification of MINDY-1^{cat}-Ub^{Prg} complex by ion exchange chromatography. SDS gel of peak fractions is shown.
(C) The crystal structure of MINDY-1^{cat} (pink, cartoon representation) with electron density for the two conformers of Ub^{Prg} (cyan and orange, sticks representation). The simulated annealing omit map is contoured at 0.6 σ.
(D) Electron density for Ub shown in two different orientations. The simulated annealing omit map is contoured at 0.6 σ. The C-terminal residues of the two conformers (R72-G75) align, and are represented as sticks. The electron density fit for these regions and the vinylthioether linkage is found to be relatively perfect.
(E) Close up view of the catalytic site with electron density for His319 and Gln131.
(F) Simulated annealing omit map of the Cys loop in the apo (orange) and complex (pink) structures.

Figure S5

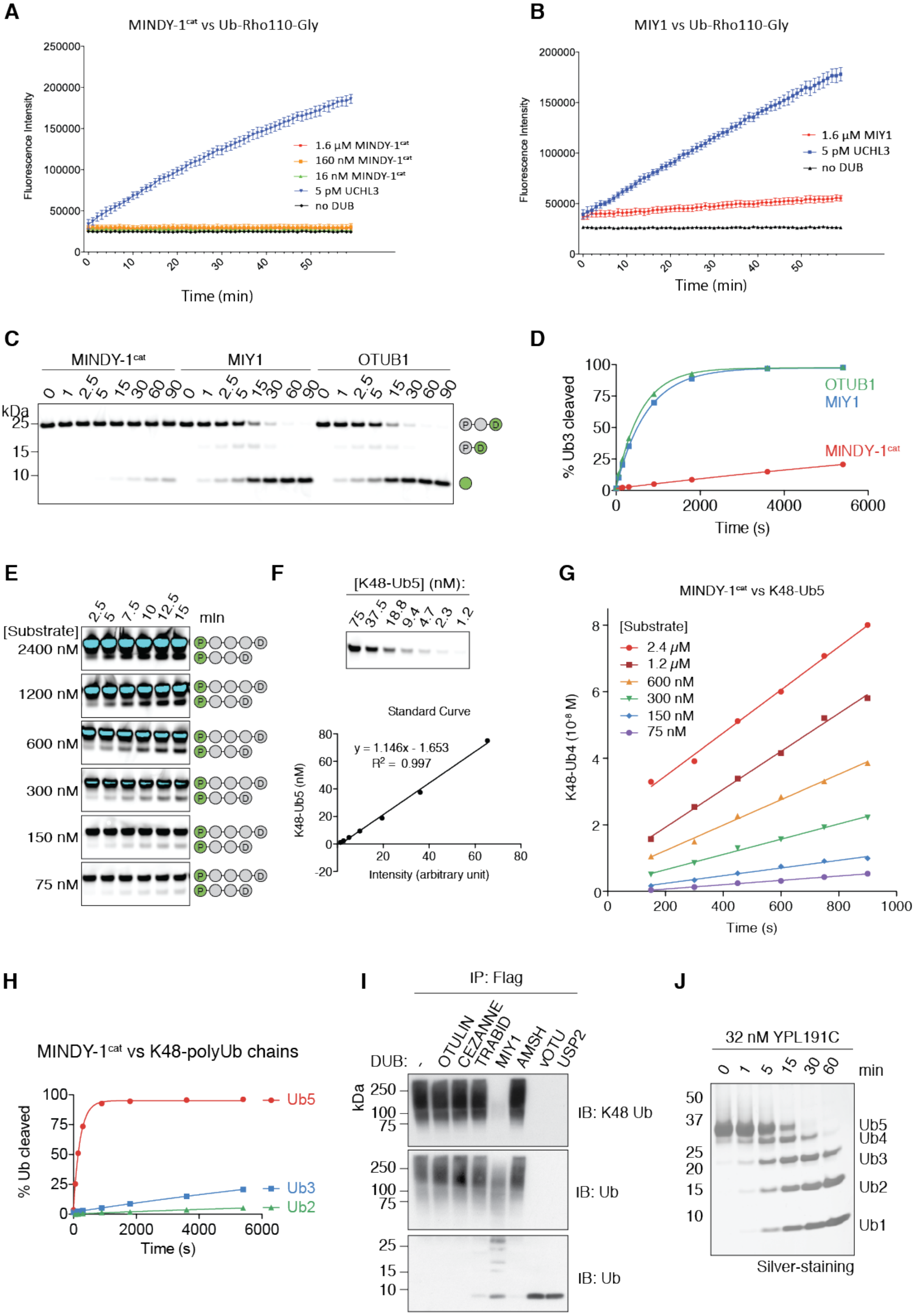

A
MINDY-1cat vs Ub-Rho110-Gly
Fluorescence Intensity
Time (min)
1.6 μM MINDY-1cat
160 nM MINDY-1cat
16 nM MINDY-1cat
5 pM UCHL3
no DUB
B
MIY1 vs Ub-Rho110-Gly
Fluorescence Intensity
Time (min)
1.6 μM MIY1
5 pM UCHL3
no DUB
C
MINDY-1cat
MIY1
OTUB1
kDa
D
% Ub3 cleaved
Time (s)
OTUB1
MIY1
MINDY-1cat
E
[Substrate]
2400 nM
1200 nM
600 nM
300 nM
150 nM
75 nM
min
F
[K48-Ub5] (nM):
Standard Curve
y = 1.146x - 1.653
R² = 0.997
K48-Ub5 (nM)
Intensity (arbitrary unit)
G
MINDY-1cat vs K48-Ub5
[Substrate]
2.4 μM
1.2 μM
600 nM
300 nM
150 nM
75 nM
K48-Ub4 (10⁻⁸ M)
Time (s)
H
MINDY-1cat vs K48-polyUb chains
% Ub cleaved
Time (s)
Ub5
Ub3
Ub2
I
IP: Flag
DUB:
OTULIN
CEZANNE
TRABID
MIY1
AMSH
vOTU
USP2
kDa
IB: K48 Ub
IB: Ub
IB: Ub
J
32 nM YPL191C
min
Ub5
Ub4
Ub3
Ub2
Ub1
Silver-staining

Figure S5. MINDY-1 cleaves polyUb chains in a step-wise manner. Related to Figure 4.
(A-B) MINDY-1^{cat} does not hydrolyze the fluourogenic DUB substrate Ub-Rho110-G. Progress curves for 50 nM Ub-Rho110-G hydrolysis using increasing concentrations of MINDY-1^{cat} (A) or MIY1 (B). UCHL3 was included as positive control.
(C) MINDY-1^{cat} cleaves K48-Ub3 poorly compared to MIY1 and OTUB1. DUBs (1 μM) were incubated with 500 nM of K48-Ub3 that has been labeled with an infrared fluorescent dye at the distal Ub (green circle) for the indicated times. Fluorescence Ub was visualized using Odyssey gel scanner at 800 nm channel.
(D) Quantification of K48-Ub3 hydrolysis by MINDY-1^{cat}, MIY1, and OTUB1 in (C). The percentage of the total intensities of Ub4, Ub3, Ub2, and Ub1 formed is shown on the y-axis. (n = 3; means ± s.d.).
(E-G) Steady state kinetics of MINDY-1^{cat} for K48-Ub5. K48-Ub5 chains that have been labeled with an infrared fluorescent dye at the proximal Ub (green circle) at different concentrations were hydrolyzed by a fixed concentration of MINDY-1^{cat} (15 nM) over the indicated times (E). Since the fluorescence label is only on one Ub molecule (proximal), the intensity on K48-Ub5 will be the same number of molecules of K48-Ub4, where distal Ub has been cleaved by MINDY-1^{cat}. Therefore, the standard curve of K48-Ub5 can be used to convert the intensity of K48-Ub4 in (E) to a molar concentration (F). The K48-Ub4 produced is plotted against time. Data was fitted to linear regression curve, where the slope is the initial velocity, V_0 (G). Figures S5E-S5G are representative data from one of the replicates that were used to derive Michaelis-Menten kinetics in Figure 4F.
(H) MINDY-1^{cat} prefers to cleave long K48-polyUb chains. The cleavage percentage of K48-Ub2 (green), K48-Ub3 (blue), and K48-Ub5 (red) by MINDY-1^{cat} over time was plotted in the same graph using the data from Figure 4C, S5D, and 4E, respectively.
(I) MINDY-1 captures K48-linked polyUb chains that contain other linkages. To analyze polyUb captured by the catalytic domain, Flag pull-downs from extracts of HEK293 cells inducibly-expressing 3xFlag-MINDY-1 C137A were incubated with a panel of linkage specific DUBs. The DUBs used and their linkage preferences are indicated in brackets: OTULIN (M1), CEZANNE (K11), TRABID (K29 and K33), MIY1 (K48), AMSH (K63), vOTU (all but M1, K27, and K29), and USP2 (all). K48 polyubiquitin chains are completely cleaved by MIY1 treatment, but are not affected by any of the other DUBs. However, only partial polyUb chains detected by anti-Ub antibody are reduced with MIY1 treatment. The remaining Ub may be a result of monoUb remnant on proteins or polyUb chains of linkage types other than K48 present as part of heterotypic chains. The release of lower molecular weight chains (Ub2-Ub5) upon MIY1 treatment suggests the presence of heterotypic chains.
(J) MIY1/YPL191C is an endo-DUB. Time course analysis of 3.5 μM pentaUb cleavage by 32 nM MIY1/YPL191C shows formation of cleavage products of all chain lengths (Ub4, Ub3, Ub2 and Ub1) at the earliest time points.

Supplemental Tables:

Table S1: cDNA constructs used in this study

Protein	Expressed protein	Tag Cleaved	Vector type	Plasmid	DU number
MINDY-1 MIU	GST-Halo-MINDY-1 388-426	Yes	Bacterial	pGEX6P1	47443
MINDY-1^{FL}/ FAM63A^{FL}	GST-MINDY-1 1-469	Yes	Bacterial	pGEX6P1	49563
FAM63B^{FL}	GST-MINDY-1 1-621	Yes	Bacterial	pGEX6P1	46765
MIY1/YPL191C^{FL}	GST-YPL191C 1-360	Yes	Bacterial	pGEX6P1	47420
YGL082W^{FL}	GST-YPL191C 1-381	Yes	Bacterial	pGEX6P1	47391
FAM188A^{FL}	GST-YPL191C 1-445	Yes	Bacterial	pGEX6P1	47870
MINDY-1^{cat}/ FAM63A^{cat}	GST-MINDY-1 110-380	Yes	Bacterial	pGEX6P1	47257
3xFlag-MINDY-1	3xFlag-MINDY-1	No	Mammalian	pcDNA5 FRT/TO	49562
3xFlag-MINDY-1 MIU*	3xFlag-MINDY-1 L415A/A416G	No	Mammalian	pcDNA5 FRT/TO	47510
3xFlag-MINDY-1 C137A	3xFlag-MINDY-1 C137A	No	Mammalian	pcDNA5 FRT/TO	47438
Ubiquitin	Ub-Intein-CBD 1-75	Yes	Bacterial	pTXB1	24149
Ubiquitin G75A/G76A	Ubiquitin G75A/G76A	n.a.	Bacterial	pET24	49488
Cys-Ubiquitin K48R	GST-Cys-Ubiquitin K48R	Yes	Bacterial	pGEX6P1	47729
Cys-Ubiquitin 1-75	GST-Cys-Ubiquitin 1-75	Yes	Bacterial	pGEX6P1	47779
Ubiquitin G75A/G76A	Ubiquitin G75A/G76A	n.a.	Bacterial	pET24	49488
Cys-Ubiquitin K48R	GST-Cys-Ubiquitin K48R	Yes	Bacterial	pGEX6P1	47729
Cys-Ubiquitin 1-75	GST-Cys-Ubiquitin 1-75	Yes	Bacterial	pGEX6P1	47779
MINDY-1^{cat} C137A	GST-MINDY-1^{cat} 110-384 C137A	Yes	Bacterial	pGEX6P1	47419
MINDY-1^{cat} H319A	GST-MINDY-1^{cat} 110-384 H319A	Yes	Bacterial	pGEX6P1	47669
MINDY-1^{cat} Q131A	GST-MINDY-1^{cat} 10-384 Q131A	Yes	Bacterial	pGEX6P1	47783
MINDY-1^{cat} Q131E	GST-MINDY-1^{cat} 110-384 Q131E	Yes	Bacterial	pGEX6P1	47832
MINDY-1^{cat} V210A	GST-MINDY-1^{cat} 110-384 V210A	Yes	Bacterial	pGEX6P1	55059
MINDY-1 W240A	GST-MINDY-1^{cat} 110-384 W240A	Yes	Bacterial	pGEX6P1	47817
MINDY-1^{cat} Y258A	GST-MINDY-1^{cat} 110-384 Y258A	Yes	Bacterial	pGEX6P1	47818
MINDY-1^{cat} F315A	GST-MINDY-1^{cat} 110-384 F315A	Yes	Bacterial	pGEX6P1	47819
MINDY-1^{cat} E263A	GST-MINDY-1^{cat} 110-384 E263A	Yes	Bacterial	pGEX6P1	47663
MINDY-1^{cat} E263R	GST-MINDY-1^{cat} 110-384 E263R	Yes	Bacterial	pGEX6P1	47683
MINDY-1^{cat} D209A	GST-MINDY-1^{cat} 110-384 D209A	Yes	Bacterial	pGEX6P1	47664
MINDY-1^{cat} D209A E263A	GST-MINDY-1^{cat} 110-384 D209A E263A	Yes	Bacterial	pGEX6P1	47697

Supplemental Materials and Methods

Protein expression and purification

Recombinant GST-fusion proteins were expressed in *E. coli* strain BL21. Cultures were grown in 2xTY media containing 50 μg/ml ampicillin to an OD_{600} of 0.6-0.8. Protein expression was induced with 300 μM IPTG and cultures grown overnight at 18 °C. Harvested cells were resuspended in GST-Lysis Buffer (50 mM Tris-HCl pH 7.5, 300 mM NaCl, 10% glycerol, 0.075% 2-mercaptoethanol, 1 mM benzamidine, 1 mM PMSF, and complete protease inhibitor cocktail (Roche)) and lysed by sonication. Bacterial lysate was clarified by centrifugation at 30,000 x g for 30 min and subsequently incubated with Glutathione Sepharose 4B resin (GE Healthcare) for 2-4 h at 4 °C. The resin bound proteins were washed extensively with high salt buffer (25 mM Tris pH 7.5, 500 mM NaCl, and 10 mM DTT) and low salt buffer (25 mM Tris-HCl pH 7.5, 150 mM NaCl, 10% glycerol, and 1 mM DTT). The GST tag was removed by overnight incubation with 3C protease at 4 °C.

Proteins were further purified by anion exchange chromatography (Resource Q, GE Healthcare Life Sciences) and eluted in a gradient with buffer Q (50 mM Tris-HCl pH 8.5, 1 M NaCl and 2 mM DTT), followed by size exclusion chromatography (Superdex 75 16/60, GE Healthcare Life Sciences) in buffer G (50 mM Tris-HCl, 150 mM NaCl, 10 mM DTT). The purified proteins were concentrated, flash frozen in liquid nitrogen and stored at -80 °C.

Ubiquitin binding domain (UBD) linkage specificity analysis

Halo-tagged MINDY-1 (388-426) (10.5 nmol) was incubated with 100 μl of the HaloLink resin (Promega) in 500 μl of the coupling buffer (50 mM Tris-HCl pH 7.5, 150 mM NaCl, 0.05% NP-40, 1 mM DTT) for 3 h at 4 °C. The UBD linkage specificity analysis was carried out by incubating 10 μl of the coupled Halo-MINDY-1 resin (388-426) with 58.5 nM of tetraUb of the indicated linkages in 500 μl of pull down buffer (50 mM Tris-HCl pH 7.5, 150 mM NaCl, 0.1% NP-40, 1 mM DTT, 0.5 mg/ml BSA) for 2 h at 4 °C. The resin was washed two times with the wash buffer (50 mM Tris-HCl pH 7.5, 250 mM NaCl, 0.2% NP-40, 1 mM DTT) and once with the coupling buffer. Captured tetraUb chains were eluted by adding LDS buffer, separated on 4-12% SDS-PAGE gel (Life Technology), and visualized by silver staining using Pierce Silver stain kit (ThermoFischer).

Deubiquitylation assays

DUBs were diluted in 50 mM Tris-HCl pH 7.5, 50 mM NaCl, 10 mM DTT and incubated at 24 °C for 10 min. The DUB assays were subsequently carried out where 2.2 μM of tetraUb of different linkage types were incubated with 1.6 μM DUB in 50 mM Tris-HCl pH 7.5, 50 mM NaCl, 10 mM DTT in a reaction volume of 10 μl. For DUB assays in Fig. 4A, 160 nM YPL191C/MIY1 was used. For DUB assays comparing activity of MINDY-1 at cleaving K48 chains, the following concentrations of Ub chains were used: 1.9 μM K48-Ub3 or 2.2 μM K48-Ub4, 3.5 μM K48-Ub5, or 3.5 μg of K48-Ub5-n. The reactions were incubated at 30 °C and stopped at different time points by adding LDS buffer. The samples were separated on 4-12% SDS-PAGE gel (Life Technology) and silver stained using Pierce Silver stain kit (ThermoFischer).

Preparation and purification of MINDY-1^{cat}~Ub^{Prg} complexes

Ubiquitin thioester was generated following cleavage of Ub-intein as described previously (Borodovsky et al., 2002). The Ub-thioester was dialyzed into HEPES buffer, pH 8.0 and 250 mM Propargylamine (Sigma) was added to the dialyzed Ub-thioester and the reaction incubated for 4 hours at 20 °C in the dark. Unreacted propargylamine was separated from propargylated Ub by size exclusion chromatography. To obtain MINDY-1^{cat}~Ub^{Prg} complexes, purified MINDY-1^{cat} was incubated with molar excess of propargylated Ub for 30 min at room temperature followed by overnight incubation at 4 °C (**Figure S4A**). For crystallization, the covalent complex was purified by anion exchange chromatography (RESOURCE Q) (**Figure S4B**) followed by SEC in buffer G. The purified complex was concentrated to 11.6 mg/ml and used for crystallization.

Crystallization, data collection and determination of MINDY-1^{cat} structure

Initial hits of crystals were obtained by screening using sitting-drop vapor diffusion method. For MINDY-1^{cat}, native crystals were grown from hanging drops containing an equal volume of protein (10mg/ml) and mother liquor containing 100 mM HEPES-Na pH 7.5, 200 mM NaCl, and 25% PEG4000. For anomalous phasing, diffraction quality crystals were obtained with macro-seeding. Native crystals were macroseeded into drops containing 1mM ethylmercuric phosphate (EMP). The crystals were flash frozen in cryoprotectant containing 100mM HEPES-Na pH 7.3, 200 mM NaCl, 35% PEG 4000 and 5% PEG 400. Interestingly, the presence of the mercury derivative also resulted in better diffraction of crystals. The data collection for anomalous and native data sets were done at Diamond light source (I02) and ID23-1 beamline, European Synchrotron Radiation Facility (ESRF) respectively.

The structure of MINDY-1^{cat} was determined by SAD phasing using data collected from crystals grown with mercury derivative. The anomalous data were processed using XDS (Kabsch, 2010) and then scaled using AIMLESS (Evans and Murshudov, 2013) within XIA2 (Winter et al., 2013). The structure was solved using the

SAS protocol of Auto-Rickshaw: the EMBL-Hamburg automated crystal structure determination platform (Panjikar et al., 2005). The partially built model obtained was further submitted to the MRSAD pipeline. The complete model for the interpretable density was built manually in Coot (Emsley et al., 2010). The native structure was determined by molecular replacement using PHASER with the partially built structure as a search model (McCoy et al., 2007). Iterative rounds of model building and refinement were performed with coot and Refmac5 (Murshudov et al., 1997). The data collection and refinement statistics for MINDY-1^{cat} structure are shown in Table 1. All the figures were made using PyMOL (http://pymol.org).

Crystallization, data collection and structure determination of MINDY-1^{cat}~Ub

Crystals for MINDY-1^{cat}~Ub (11.6 mg/ml) grew in hanging drops from conditions with 100mM MES pH 6.5, 10% Dioxane and 1.6 M ammonium sulphate. The crystals were directly harvested and vitrified. The MINDY-1^{cat}~Ub crystals diffracted to 2.5 Å at ID29 beamline, ESRF, France. The data was processed using XDS and scaled and merged using AIMLESS. The structure of the complex was solved by molecular replacement using MINDY-1^{cat} and Ub (PDB ID: 1UBQ) as search models. In the MINDY-1^{cat}~Ub structure, clear electron density is visible for MINDY-1^{cat}. In contrast, electron density appears to be fractured or disordered in stretches for Ub. When refined at lower resolution two alternate conformations of Ub could be docked into the electron density. The structure was finally refined at 2.65 Å with occupancy of the two conformers set to ~0.5 each. The final data collection and refinement statistics for the MINDY-1^{cat}~Ub structure is shown in Table 1.

Ubiquitin-Rhodamine110-Glycine hydrolysis assay

Ub-Rho110-G was expressed and purified as described previously (Hassiepen et al., 2007; Ritorto et al., 2014). In the assay, 50 nM Ub-Rho110-G was incubated with 5 pM – 1.6 µM DUB in a final volume of 20 µl reaction buffer (50 mM Tris-HCl pH 7.5, 50 mM NaCl, 10 mM DTT, 0.1 mg/ml BSA). Samples were prepared in triplicates in 96-well plates and analysed using PHERAstar FS (BMG Labtech) Excitation/Emission 485/520 for 60 cycles with 60 s of interval.

Generation of fluorescently labeled K48-polyUb chains

Assembly of K48-Ub2 that contains Cys residue at the distal Ub (K48-Ub2 [Cys-Ub_{distal}])

K48-Ub2 [Cys-Ub_{distal}] was assembled enzymatically using Ub G75A/G76A as the Ub acceptor and Ub K48R with Cys residue upstream of M1 (Cys-Ub K48R) as the Ub donor. The assembly reaction was carried out for 1 h at 30 °C in 1 ml reaction containing 1 mM Ub G75A/G76A, 1 mM Cys-Ub K48R, 0.5 µM UBE1, 15 µM UBE2R1, 10 mM ATP, 50 mM Tris-HCl (pH 7.5), 10 mM MgCl2, and 0.6 mM DTT. K48-Ub2 [Cys-Ub_{distal}] was purified as described previously (Kristariyanto et al., 2015).

Assembly of K48-Ub3 that contains Cys residue at the distal Ub (K48-Ub3 [Cys-Ub_{distal}])

K48-Ub3 [Cys-Ub_{distal}] was assembled by capping a pre-assembled K48-Ub2 by Cys-Ub K48R (donor Ub). The assembly reaction was carried out for 2 h at 30 °C in 1 ml reaction containing 250 µM K48-Ub2, 1.25 mM Cys-Ub K48R, 0.5 µM UBE1, 15 µM UBE2R1, 10 mM ATP, 50 mM Tris-HCl (pH 7.5), 10 mM MgCl2, and 0.6 mM DTT. K48-Ub3 [Cys-Ub_{distal}] was purified as described previously (Kristariyanto et al., 2015).

Assembly of K48-Ub5 that contains Cys residue at the proximal Ub (K48-Ub5 [Cys-Ub_{prox}])

K48-Ub5 [Cys-Ub_{prox}] was assembled by extending Ub 1-75 (Ub acceptor), which contains Cys residue upstream of M1 (Cys-Ub 1-75), with a pre-assembled K48-Ub2. The assembly reaction was carried out for 1.5 h at 30 °C in 1 ml reaction containing 250 µM K48-Ub2, 1.25 mM Cys-Ub 1-75 (acceptor/proximal Ub), 0.5 µM UBE1, 15 µM UBE2R1, 10 mM ATP, 50 mM Tris-HCl (pH 7.5), 10 mM MgCl2, and 0.6 mM DTT. The reaction produced K48-Ub3 [Cys-Ub_{prox}] and smaller quantity of K48-Ub5 [Cys-Ub_{prox}]. K48-Ub5 [Cys-Ub_{prox}] was purified as described previously (Kristariyanto et al., 2015).

Fluorescence labeling

The infrared dye, IRDye® 800CW Maleimide, was purchased from LI-COR. IRDye 800CW was conjugated to Cys residue of the Cys-Ub mutant using standard thiol-maleimide conjugation procedure. Briefly, K48-polyUb chains were diluted in 20 mM Tris-HCl pH 7.5, 500 µM TCEP to a concentration of 50-100 µM. The protein samples were purged with argon and kept at room temperature for 30 min. Four-fold excess of IRDye 800CW was added to the reaction mix. The reaction was purged with argon and incubated at room temperature for 2 h. To stop the reaction, excess amount of 2-mercaptoethanol was added. IRDye 800CW-labeled K48-chains were purified using PD-10 desalting column (GE Healthcare).

Quantitative fluorescence deubiquitylation assays

DUB assay reactions were performed at 30 °C by adding 10 µl pre-activated DUB and 10 µl of fluorescence-labeled K48-polyUb chains. The reaction mixtures contained 1 µM DUB, 500 nM fluorescence-labeled K48-polyUb chains, 50 mM Tris-HCl pH 7.5, 50 mM NaCl, 10 mM DTT, and 0.25 mg/ml BSA. At the indicated time points, 2.5 µl of the samples was transferred to 7.5 µl LDS sample buffer to quench the reaction. Samples were separated on 4-12% SDS-gel and visualized using Odyssey imaging system (LI-COR) at 800 nm channel. Intensities of K48-chains were quantified using Image Studio Lite (LI-COR).

Steady-state deubiquitylation assay

Steady-state enzyme kinetics of MINDY-1^{cat} in hydrolyzing K48-Ub5 were measured in reactions containing 15 nM MINDY-1^{cat} and 75, 150, 300, 600, 1200, or 2400 nM fluorescence-labeled K48-Ub5 in 50 mM Tris-HCl pH7.5, 50 mM NaCl, 10 mM DTT, and 0.25 mg/ml BSA at 30 °C. Aliquots of 2.5 µl were mixed with 7.5 µl LDS sample buffer at 2.5, 5, 7.5, 10, 12.5, and 15 min. Samples were separated and the formation of K48-Ub4 was analyzed and quantified as described above (**Figure S5E**). To convert the quantified intensity of the formed K48-Ub4 to molar concentration, a standard curve of 1.17 to 75 nM IR-K48-Ub5 was made (**Figure S5F**). Since the fluorescence label is only on one Ub molecule (proximal), the intensity of K48-Ub5 will translate to the same number of molecules of K48-Ub4, or equal molar concentrations. The amount of K48-Ub4 formed was plotted against time and the data was fitted to a linear regression curve, where slope is the initial velocity, V_0 ($M.s^{-1}$) (Figure S5G). The initial velocity values were then plotted against substrate concentration and the data was fitted to the Michaelis-Menten equation:

$$V_0 = \frac{V_{max}[\mathrm{S}]}{K_m + [\mathrm{S}]} = \frac{k_{cat}[\mathrm{E}][\mathrm{S}]}{K_m + [\mathrm{S}]}$$

where V_0 is the initial velocity, V_{max} is the maximum velocity, [S] is substrate concentration, K_m is the Michaelis constant, and k_{cat} is the turnover rate. Data fitting was carried out using GraphPad Prism 5 software.

Cell culture and transfection

Flp-In T-REx HEK293 cells were cultured in Dulbecco's modified Eagle's medium (DMEM) containing 10% fetal bovine serum (FBS), 2 mM L-glutamine, 100 units/ml penicillin, 100 µg/ml streptomycin, 100 µg/ml Zeocin and 15 µg/ml Blasticidin. Tetracycline-inducible stable cell lines were generated by transfecting Flp-In T-REx HEK293 cells with the indicated constructs. Transfection was performed by GeneJuice Transfection Reagent (Novagen). Protein expression was induced by addition of fresh tissue culture medium containing tetracycline at a final concentration of 1 µg/ml for 24 h.

Flag immunoprecipitations

Cells were lysed in lysis buffer (1% Triton-X, 50 mM Tris-HCl pH 7.5, 0.1 mM EDTA, 0.1 mM EGTA, 10 mM sodium glycerol 2-phosphate, 50 mM sodium fluoride, 5 mM sodium pyrophosphate, 270 mM sucrose, 1 mM sodium orthovanadate, 25 mM iodoacetamide, 1 mM AEBSF, 0.02% benzonase) supplemented with protease inhibitor cocktail. The cell lysates were incubated with Anti-Flag M2 agarose resins (Sigma Aldrich) for 2 hours at 4 °C. The resins were washed 3 times with lysis buffer and the captured proteins were analyzed by immunoblotting. Where indicated, the Flag immunoprecipitated materials were treated with a panel of linkage specific DUBs as described previously (Kristariyanto et al., 2015).

Supplemental References:

Akutsu, M., Ye, Y., Virdee, S., Chin, J.W., and Komander, D. (2011). Molecular basis for ubiquitin and ISG15 cross-reactivity in viral ovarian tumor domains. Proc. Natl. Acad. Sci. USA *108*, 2228–2233.

Borodovsky, A., Ovaa, H., Kolli, N., Gan-Erdene, T., Wilkinson, K.D., Ploegh, H.L., and Kessler, B.M. (2002). Chemistry-based functional proteomics reveals novel members of the deubiquitinating enzyme family. Chem. Biol. *9*, 1149–1159.

Emsley, P., Lohkamp, B., Scott, W.G., and Cowtan, K. (2010). Features and development of Coot. Acta Crystallogr. D Biol. Crystallogr. *66*, 486–501.

Evans, P.R., and Murshudov, G.N. (2013). How good are my data and what is the resolution? Acta Crystallogr. D Biol. Crystallogr. *69*, 1204–1214.

Hassiepen, U., Eidhoff, U., Meder, G., Bulber, J.-F., Hein, A., Bodendorf, U., Lorthiois, E., and Martoglio, B. (2007). A sensitive fluorescence intensity assay for deubiquitinating proteases using ubiquitin-rhodamine110-glycine as substrate. Anal. Biochem. *371*, 201–207.

Kabsch, W. (2010). XDS. Acta Crystallogr. D Biol. Crystallogr. *66*, 125–132.

Kamphuis, I.G., Kalk, K.H., Swarte, M.B., and Drenth, J. (1984). Structure of papain refined at 1.65 A resolution. J. Mol. Biol. *179*, 233–256.

Kristariyanto, Y.A., Rehman, S.A.A., Campbell, D.G., Morrice, N.A., Johnson, C., Toth, R., and Kulathu, Y. (2015). K29-Selective Ubiquitin Binding Domain Reveals Structural Basis of Specificity and Heterotypic Nature of K29 Polyubiquitin. Mol. Cell *58*, 83–94.

McCoy, A.J., Grosse Kunstleve, R.W., Adams, P.D., Winn, M.D., Storoni, L.C., and Read, R.J. (2007). Phaser crystallographic software. J. Appl. Crystallogr. *40*, 658–674.

Murshudov, G.N., Vagin, A.A., and Dodson, E.J. (1997). Refinement of macromolecular structures by the maximum-likelihood method. Acta Crystallogr. D Biol. Crystallogr. *53*, 240–255.

Panjikar, S., Parthasarathy, V., Lamzin, V.S., Weiss, M.S., and Tucker, P.A. (2005). Auto-rickshaw: an automated crystal structure determination platform as an efficient tool for the validation of an X-ray diffraction experiment. Acta Crystallogr. D Biol. Crystallogr. *61*, 449–457.

Penengo, L., Mapelli, M., Murachelli, A.G., Confalonieri, S., Magri, L., Musacchio, A., Di Fiore, P.P., Polo, S., and Schneider, T.R. (2006). Crystal structure of the ubiquitin binding domains of rabex-5 reveals two modes of interaction with ubiquitin. Cell *124*, 1183–1195.

Ritorto, M.S., Ewan, R., Perez-Oliva, A.B., Knebel, A., Buhrlage, S.J., Wightman, M., Kelly, S.M., Wood, N.T., Virdee, S., Gray, N.S., Morrice, N.A., Alessi, D.R., and Trost, M. (2014). Screening of DUB activity and specificity by MALDI-TOF mass spectrometry. Nat. Commun. *5*, 4763.

Winter, G., Lobley, C.M.C., and Prince, S.M. (2013). Decision making in xia2. Acta Crystallogr. D Biol. Crystallogr. *69*, 1260–1273.
